# Synthesis and Antimicrobial Evaluation of Side-Chain Derivatives based on Eurotiumide A

**DOI:** 10.3390/md18020092

**Published:** 2020-01-30

**Authors:** Atsushi Nakayama, Hideo Sato, Tenta Nakamura, Mai Hamada, Shuji Nagano, Shuhei Kameyama, Yui Furue, Naoki Hayashi, Go Kamoshida, Sangita Karanjit, Masataka Oda, Kosuke Namba

**Affiliations:** 1 Graduate School of Pharmaceutical Sciences and Research Cluster on “Innovative Chemical Sensing”, Tokushima University, 1-78-1 Shomachi, Tokushima 770-8505, Japan; hideo1995214@gmail.com (H.S.); c401603069@tokushima-u.ac.jp (T.N.); c401403070@tokushima-u.ac.jp (M.H.); c401941004@tokushima-u.ac.jp (S.N.); c401931030@tokushima-u.ac.jp (S.K.); karanjit@tokushima-u.ac.jp (S.K.); 2 Department of Microbiology and Infection Control Sciences, Kyoto Pharmaceutical University, Misasaginakauchi-cho, Yamashita-Ku, Kyoto 607-8414, Japan; mametty1214@gmail.com (Y.F.); nhayashi@mb.kyoto-phu.ac.jp (N.H.); kamoshida@mb.kyoto-phu.ac.jp (G.K.);

**Keywords:** antibiotics, natural product, *P. gingivalis*, methicillin-resistant *S. aureus*

## Abstract

Side-chain derivatives of eurotiumide A, a dihydroisochroman-type natural product, have been synthesized and their antimicrobial activities described. Sixteen derivatives were synthesized from a key intermediate of the total synthesis of eurotiumide A, and their antimicrobial activities against two Gram-positive bacteria, methicillin-susceptible and methicillin-resistant *Staphylococcus aureus* (MSSA and MRSA), and a Gram-negative bacterium, *Porphyromonas gingivalis*, were evaluated. The results showed that derivatives having an iodine atom on their aromatic ring instead of the prenyl moiety displayed better antimicrobial activity than eurotiumide A against MSSA and *P. gingivalis*. Moreover, we discovered that a derivative with an isopentyl side chain, which is a hydrogenated product of eurotiumide A, is the strongest antimicrobial agent against all three strains, including MRSA.

## 1. Introduction

**Figure 1:**
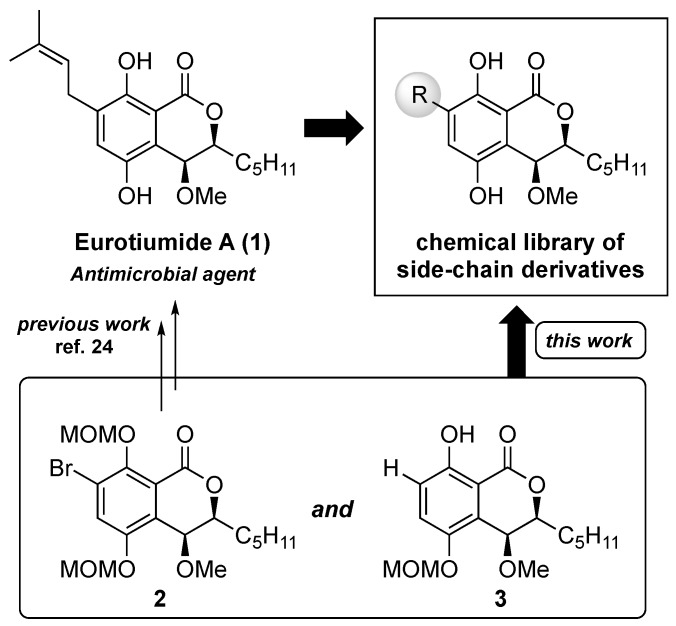
Concept of construction of the chemical library of the side chain-derivatives of eurotiumide A (**1**).

Humans have always struggled against infectious diseases [1,2,3,4,5] and in relatively recent times have developed various antimicrobial therapies [6,7,8]. Since the discovery of penicillin [9], various natural products having antimicrobial activity have been discovered [10,11,12,13,14,15,16], and the majority of clinically used antibiotics are either natural products, semisynthetic derivatives, or compounds derived from them [17,18,19]. Despite the presence of many excellent antibiotics, multidrug-resistant bacterial pathogens have emerged all over the world [20,21,22], and the development of novel and effective antimicrobial agents against many kinds of pathogenic bacteria, including methicillin-resistant *Staphylococcus aureus* (MRSA), should remain a continuous mission for medicinal chemists. In 2014, Wang and co-workers discovered eurotiumides, which are novel dihydroisocoumarin-type natural products, from a gorgonian-derived fungus, *Eurotium* sp. XS-200900E6 [23]. Among the series of eurotiumides, eurotiumide A (**1**), having *cis* configurations at H3/H4, exhibited potent antimicrobial activities against *Staphylococcus epidermidis*, *Bacillus cereus*, *Vibrio anguillarum*, and *Escherichia coli*. Based on that report, although **1** seems to be an attractive seed compound for antibiotics, further antimicrobial investigation and a structure–activity relationship study of **1** are needed. In particular, because there is a chance that modification of the side chain of the aromatic ring could improve antimicrobial activity and the spectrum, a structure–activity relationship study of the substituent effect of the aromatic ring is essential for discovering promising candidates for antimicrobial agents. Recently, we reported the first asymmetric total syntheses of (−)-eurotiumide A (**1**) and (+)-eurotiumide B and revised their reported structures [24]. In our synthetic route, the prenyl side chain of the aromatic ring was introduced in the late stage by the Stille coupling reaction with the key intermediate **2**. Based on our previous results, we considered that a number of derivatives of **1**, which have a variety of kinds of side-chain moiety, could be obtained from the common intermediate **2** and non-substituted compound **3** in the late stage of synthesis (Figure 1).

In this work, as part of our continuing research [24,25], we constructed a chemical library of the side-chain derivatives of eurotiumide A (**1**) to elucidate the effects of the side chains of the aromatic rings and to develop antimicrobial agents against methicillin-susceptible *S. aureus* (MSSA) and methicillin-resistant *S. aureus* (both Gram-positive bacteria), as well as *Porphyromonas gingivalis* (a Gram-negative bacterium).

## 2. Results and Discussion

### 2.1. Synthesis of the Side-Chain Derivatives of Eurotiumide A

**Figure 2:**
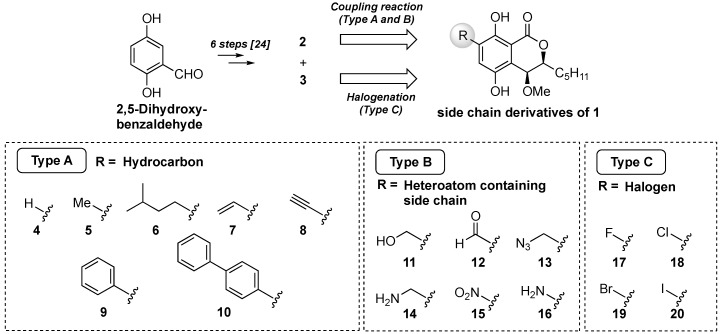
Synthetic plan of the side-chain derivatives of eurotiumide A (**1**).

Our synthetic plan is shown in Figure 2. We planned to introduce three types of functional groups: a hydrocarbon group, including hydrogen, alkyl, and aromatic rings (Type A); a heteroatom and heteroatom-containing alkyl group (Type B); and halogen atoms group (Type C). The derivatives of groups A and B could be derived from **2** by the cross-coupling reaction and functional group transformation. The halogenated derivatives (Type C) would be obtained from **3** by direct introduction of the halogen atoms. Although Wang et al. isolated the natural eurotiumide A (**1**) as a racemic form, they evaluated the antimicrobial activities of its enantiomers after separation by chiral HPLC and revealed that there was no significant difference between the enantiomers [23]. From the viewpoint of the efficiency of compound supply, we decided to make racemic compounds.

**Scheme 1:**
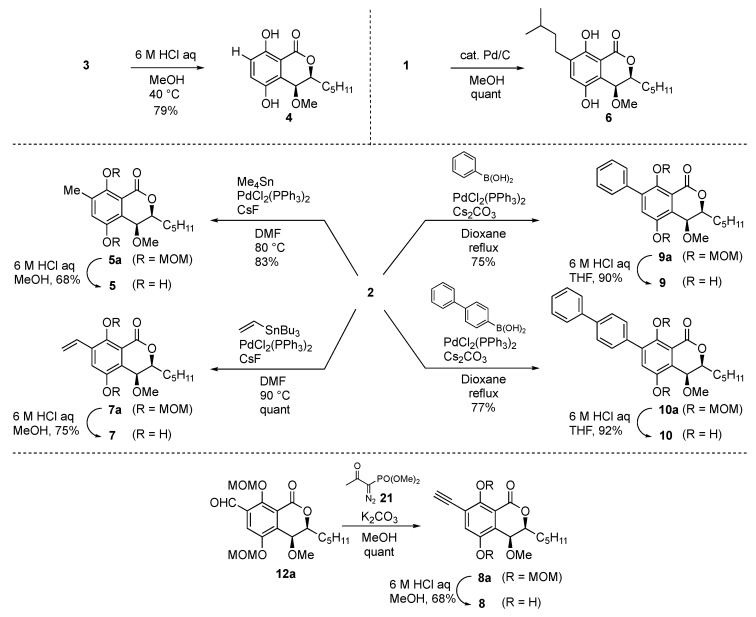
Synthesis of the hydrocarbon derivatives (type A).

First, we initiated the syntheses of the derivatives of group A (Scheme 1). The non-substituted derivative **4** was obtained from **3** by deprotection of the diMOM group with aqueous 6 M HCl in methanol at 40 °C in 79% yield. Catalytic hydrogenation of eurotiumide A (**1**) gave the isopentyl derivative **6** in quantitative yield. Methyl and vinyl groups were introduced by the Stille coupling reaction with **2** to afford methyl derivative **5a** and styrene derivative **7a** in 83% and quantitative yields, respectively. Phenyl derivative **9a** and biphenyl derivative **10a** were obtained from **2** by the Suzuki–Miyaura cross coupling reaction with the corresponding boronic acids in 75% and 77% yields, respectively. Deprotection of the diMOM group of derivatives **5a**, **7a**, **9a**, and **10a** then gave the corresponding desired products (**5**, **7**, **9**, and **10**). We tried to introduce the alkyne group by the Sonogashira coupling reaction; however, the desired alkyne product was obtained in only 12% yield. To improve the reaction yield, the Seyferth–Gilbert homologation using the Ohira–Bestmann reagent **21** was applied to the aldehyde derivative **12a** (vide infra) and afforded the desired alkyne **8a** in quantitative yield. After acidic treatment of **8a**, the alkyne derivative **8** was obtained in 68% yield.

**Scheme 2:**
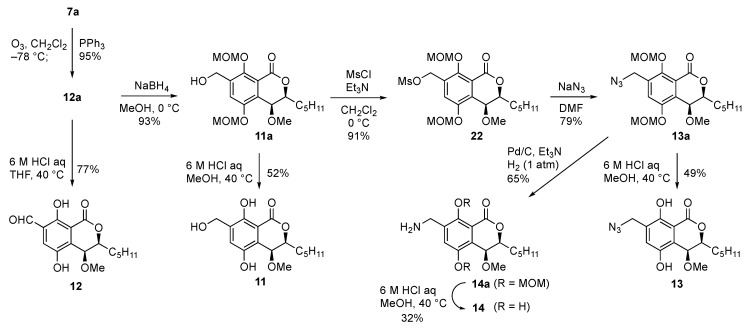
Synthesis of the derivatives having heteroatom-containing side chains (type B).

With type A derivatives in hand, we turned our attention to preparing type B derivatives having heteroatom-containing side chains (Scheme 2). For the introduction of an alkyl group containing heteroatoms, we chose the styrene derivative **7a** as a starting point. Ozonolysis of the alkene moiety of **7a** afforded the diMOM-protected benzaldehyde **12a** in excellent yield. Acidic treatment of **12a** gave the desired deprotected benzaldehyde derivative **12** in 77%. On the other hand, reduction of the aldehyde moiety of **12a** with sodium borohydride to give the benzyl alcohol **11a** and the deprotection furnished the hydroxymethyl derivative **11** in moderate yield. To introduce a nitrogen group at the benzyl position of **11a**, the primary alcohol moiety was converted to a mesyl group (**22**) and a nucleophilic substitution reaction with sodium azide afforded diMOM-protected azide **13a** in good yield. Derivative **13a** was treated with aqueous 6 M HCl in MeOH to furnish the desired dihydroxy azide derivative **13**. We then tried to convert the azide into an amine functionality. After several attempts, we found that addition of triethylamine was crucial to keep the reaction clean and we succeeded to get **14a**. Then, deprotection of the diMOM group gave the desired aminomethyl derivative **14**.

**Scheme 3:**
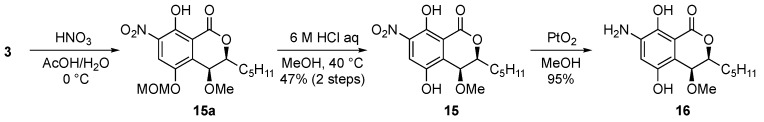
Synthesis of nitro and aniline derivatives.

Next, a nitration reaction was conducted with non-substituted derivative **3** by adding HNO_3_ in AcOH to afford monoMOM-protected nitro derivative **15a** as a crude product; then it was deprotected under acidic condition to give the nitro derivative **15** (Scheme 3). After that, hydrogenation with Adam’s catalyst produced the aniline derivative **16** from **15**.

**Scheme 4:**
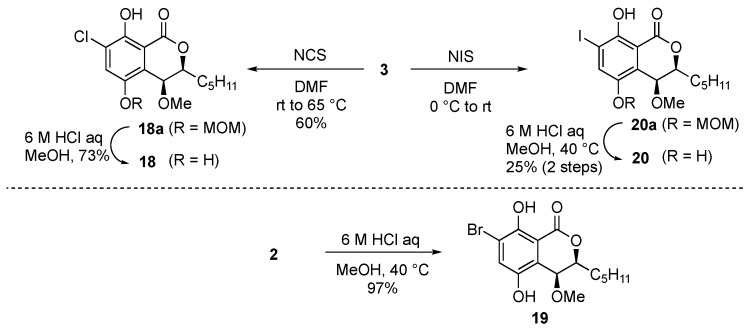
Synthesis of halogenated derivatives (type C).

Finally, we tried to synthesize the halogenated derivatives (Scheme 4). Chloro and iodo groups were introduced to treat **3** with *N*-chlorosuccinimide and *N*-iodosuccinimide in DMF to afford the chloro derivative **18a** and the iodo derivative **20a**, respectively. The diMOM groups of **18a** and **20a** were then deprotected under acidic conditions to afford the desired **18** and **20**. Bromo derivative **19** was obtained from **2** in 97% yield by acid treatment to cleave the diMOM group. However, despite several efforts to introduce fluorine to the aromatic ring from **3**, we could not get the desired fluoro derivative **17**. We also tried the Sandmeyer reaction with **16** but did not obtain the desired **17**.

### 2.2. Antimicrobial Evaluation of Synthesized Derivatives

**Figure 3:**
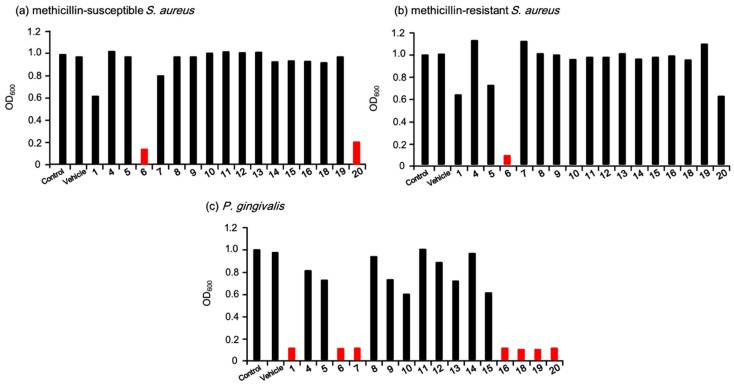
Initial screening of antimicrobial activity against (**a**) methicillin-susceptible *S. aureus*, (**b**) methicillin-resistant *S. aureus*, and (**c**) *P. gingivalis*. The terminal concentration was 10 µM.

After the initially set derivatives of eurotiumide A were synthesized, the first antimicrobial activity screening was conducted against the Gram-positive MSSA and MRSA as well as the Gram-negative *P. gingivalis* in 10 µM solutions of the synthesized derivatives to narrow down the promising antimicrobial candidates. The results are depicted in Figure 3. (+/−)-Eurotiumide A (**1**) exhibited mild antimicrobial activity against MSSA at this concentration (Figure 3a). While most of the derivatives did not show antimicrobial activity against this strain, the isopentyl derivative **6** and the iodo derivative **20** exhibited more potent antimicrobial activity than **1**. Next, we tested the same screening against MRSA (Figure 3b). Most of the derivatives that displayed good activity against MSSA showed no antimicrobial activity against MRSA. Even natural product **1** and the iodo derivative **20** also did not show good antimicrobial activity against MRSA. Surprisingly, only the isopentyl derivative **6**, which was a reduced derivative of **1**, was found to have good antimicrobial activity against MRSA. We also conducted antimicrobial screening against *P. gingivalis* (Figure 3c). Unlike the case with *S. aureus*, many derivatives, specifically eurotiumide A (**1**), isopentyl derivative **6**, vinyl derivative **7**, aniline derivative **16**, and three halogenated derivatives (**18**, **19**, **20**), were effective against *P. gingivalis*.

Since we acquired promising agents against all three strains, we determined the IC_50_ values of these candidates (Table 1). The IC_50_ values of the isopentyl derivative **6** and the iodo derivative **20** against MSSA were 5.6 µM (2.0 µg/mL) and 9.0 µM (3.7 µg/mL), respectively. Moreover, the IC_50_ value of **6** against MRSA was 4.3 µM (1.5 µg/mL), which is the same level of activity against MSSA. The IC_50_ values of these seven candidates (**1**, **6**, **7**, **16**, **18**, **19**, and **20**) against *P. gingivalis* ranged from 2.0 to 7.0 µM. We also checked the cytotoxicity of three compounds (**1**, **6**, and **20**) against the A549 cell line, and these three compounds were non-toxic in 10 µM.

In this study, we discovered that the isopentyl derivative **6**, which is a one-point modified compound of natural product **1**, and the iodo derivative **20** have superior antimicrobial activity to **1** against MSSA and *P. gingivalis*. Although **20** did not exhibit good efficacy against MRSA, **6** was found to maintain antimicrobial activity against these three strains, including MRSA. These results indicate that *S. aureus* is sensitive to changes in the side chain of the aromatic ring and that MRSA can distinguish the subtle difference between prenyl and isopentyl moieties. Moreover, the weak antimicrobial activity of **1** against MRSA suggests a binding affinity between **1** and the penicillin binding protein 2’ [26], which is the main resistance mechanism of MRSA against antibiotics. The inhibition of cell wall synthesis seems to be the mode of action of **1**, although a more detailed study is needed to clarify the mode of action of **6** and **20**. On the other hand, we found that several compounds having alkyl and halogenated side chains well suppressed the increase in *P. gingivalis*.

## 3. Materials and Methods 

### 3.1. Preparation of Eurotiumide A Derivatives.

#### 3.1.1. General Procedure

All the reactions were carried out in a round-bottomed flask with an appropriate number of necks and side arms connected to a three-way stopcock and/or a rubber septum cap under an argon atmosphere. All vessels were first evacuated by rotary pump and then flushed with argon prior to use. Solutions and solvents were introduced by hypodermic syringe through a rubber septum. During the reaction, the vessel was kept under a positive pressure of argon. Dry THF was freshly prepared by distillation from benzophenone ketyl before use. Anhydrous CH_2_Cl_2_, DMF, ethanol, MeCN, methanol, pyridine, and toluene were purchased from Kanto Chemical Co. Inc. Infrared (IR) spectra were recorded on a JASCO FT/IR-4100 spectrophotometer using a 5 mm KBr plate. Wavelengths of maximum absorbance are quoted in cm^−1^. 1H-NMR spectra were recorded on a JEOL ECA–400 (400 MHz), Bruker AV-400N (400 MHz), and Bruker AV–500 (500 MHz) in CDCl_3_. Chemical shifts are reported in parts per million (ppm), and signals are expressed as singlet (s), doublet (d), triplet (t), multiplet (m), broad (br), and overlapped. 13C-NMR spectra were recorded on a JEOL ECA–400 (100 MHz), Bruker AV–400N (100 MHz), and Bruker AV–500 (125 MHz) in CDCl_3_. Chemical shifts are reported in parts per million (ppm) (see Supplementary Materials). High resolution mass (HRMS) spectra were recorded on a Thermo Scientific Exactive. All melting points were measured with a Yanaco MP-500D. Analytical thin layer chromatography (TLC) was performed using 0.25 mm E. Merck Silica gel (60F-254) plates. Reaction components were visualized phosphomolybdic acid or ninhydrin or *p*-anisaldehyde in 10% sulfuric acid in ethanol. Kanto Chem. Co. Silica Gel 60N (particle size 0.040–0.050 mm) was used for column chromatography. 

#### 3.1.2. Synthesis of (3*S*,4*S*)-5,8-dihydroxy-4-methoxy-3-pentylisochroman-1-one (**4**)

To a solution of bromo compound **3** (10.0 mg, 30.8 µmol) in MeOH (2.3 mL) was added 6 M aqueous HCl (0.77 mL) at 0 °C. After stirring for 30 min at 40 °C, the reaction was quenched by adding saturated aqueous NaHCO_3_ at 0 °C. The mixture was extracted with EtOAc (×3) and the combined organic layers were washed with brine, dried over Na_2_SO_4_, filtered, and concentrated under reduced pressure. The residue was purified by preparative thin layer chromatography (PTLC) (EtOAc:*n*-hexane = 3:7) to give non-substituted derivative **4** (6.8 mg, 79%) as a white solid. m.p. 120–121 °C; 1H-NMR (400 MHz, CDCl_3_) δ 10.62 (1H, s), 7.06 (1H, d, *J* = 9.0 Hz), 6.91 (1H, d, *J* = 9.0 Hz), 5.89 (1H, br-s), 4.77 (1H, d, *J* = 2.7 Hz), 4.50 (1H, ddd, *J* = 2.7, 5.4, 8.3 Hz), 3.40 (3H, s), 1.95 (1H, m), 1.85 (1H, m), 1.70–1.50 (1H, overlapped), 1.46 (1H, m), 1.40–1.25 (4H, overlapped), 0.91 (3H, t, *J* = 6.8 Hz); 13C-NMR (100 MHz, CDCl_3_) δ 169.0, 156.2, 145.7, 125.1, 121.7, 118.8, 107.6, 81.4, 69.8, 56.8, 31.6, 29.8, 24.9, 22.5, 14.0.; IR (KBr) 3219, 2955, 2924, 2860, 1661, 1586, 1471, 1293, 1204, 905 cm^−1^; HRMS (ESI) *m*/*z* (M + Na)^+^ calculated for (C_15_H_20_O_5_Na)^+^ 303.1208, found 303.1200.

#### 3.1.3. Synthesis of (3*S*,4*S*)-5,8-dihydroxy-7-isopentyl-4-methoxy-3-pentylisochroman-1-one (**6**)

To a solution of eurotiumide A (**1**) (1.6 mg, 4.6 µmol) in MeOH (0.23 mL) was added Pd/C (1.6 mg, 100 w/w%) at room temperature. After stirring for 1.5 h under hydrogen atmosphere (balloon), the reaction mixture was passed through Celite and the organic solvent was removed under reduced pressure. The residue was purified with flash column chromatography (EtOAc:*n*-hexane = 2:3) to give isopentyl derivative **6** (1.4 mg, 88%) as a white wax. 1H-NMR (500 MHz, CDCl_3_) δ 10.91 (1H, s), 6.93 (1H, s), 5.62 (1H, br-s), 4.74 (1H, d, *J* = 2.5 Hz), 4.48 (1H, ddd, *J* = 2.6, 5.4, 8.6 Hz), 3.38 (3H, s), 2.62 (2H, m), 1.95 (1H, m), 1.85 (1H, m), 1.65–1.50 (2H, overlapped), 1.50–1.40 (3H, overlapped), 1.40–1.30 (4H, overlapped), 0.95 (6H, d, *J* = 6.3 Hz), 0.90 (3H, *J* = 6.9 Hz); 13C-NMR (125 MHz, CDCl_3_) δ 169.4, 154.7, 145.0, 133.6, 124.8, 118.6, 106.8, 81.4, 69.9, 56.6, 38.4, 31.6, 29.8, 29.7, 27.9, 27.5, 14.9, 22.5, 14.0.; IR (KBr) 3290, 2956, 2927, 2870, 1761, 1445, 1171, 807 cm^−1^; HRMS (ESI) *m*/*z* (M + H)^+^ calculated for (C_20_H_31_O_5_)^+^ 351.2171, found 351.2177. 

#### 3.1.4. (3*S*,4*S*)-4-methoxy-5,8-bis(methoxymethoxy)-7-methyl-3-pentylisochroman-1-one (**5a**)

To a solution of bromo compound **3** (40.0 mg, 89.4 µmol) and CsF (16.3 mg, 107 µmol) in degassed DMF (0.45 mL) were added Me_4_Sn (15 µL, 107 µmol) and PdCl_2_(PPh_3_)_2_ (6.3 mg, 8.94 µmol) at room temperature. After stirring for 50 min at 80 °C, the reaction was quenched by adding water. The mixture was extracted with EtOAc (×3) and the combined organic layers were washed with brine, dried over Na_2_SO_4_, filtered, and concentrated under reduced pressure. The residue was purified with flash column chromatography (EtOAc:*n*-hexane = 3:7) to give diMOM-protected methyl derivative **5a** (28.5 mg, 83%) as a yellow amorphous. 1H-NMR (400 MHz, CDCl_3_) δ 7.255 (1H, s), 5.21 (2H, s), 5.10 (1H, d, *J* = 6.8 Hz), 5.07 (1H, d, *J* = 6.8 Hz), 4.59 (1H, d, *J* = 1.5 Hz), 4.26 (1H, ddd, *J* = 1.5, 5.9, 7.5 Hz), 3.60 (3H, s), 3.50 (3H, s), 3.30 (3H, s), 2.39 (3H, s), 2.02 (1H, m), 1.81 (1H, m), 1.70–1.50 (1H, overlapped), 1.43 (1H, m), 1.40–1.25 (4H, overlapped), 0.91 (3H, t, *J* = 6.8 Hz); 13C-NMR (125 MHz, CDCl_3_) δ 162.4, 152.3, 149.8, 135.7, 126.3, 121.3, 118.7, 101.5, 95.0, 80.9, 68.2, 57.5, 56.7, 56.4, 31.6, 30.6, 24.9, 22.6, 17.6, 14.0.; IR (KBr) 2958, 2927, 2858, 2828, 1728, 1478, 1153 cm^−1^; HRMS (ESI) *m*/*z* (M + H)^+^ calculated for (C_20_H_31_O_7_)^+^ 383.2070, found 383.2069. 

#### 3.1.5. (3*S*,4*S*)-5,8-dihydroxy-4-methoxy-7-methyl-3-pentylisochroman-1-one (**5**)

To a solution of diMOM-protected methyl derivative **5a** (10.0 mg, 26.0 µmol) in MeOH (2.0 mL) was added 6 M aqueous HCl (0.65 mL) at 0 °C. After stirring for 1 h at 40 °C, the reaction was quenched by adding saturated aqueous NaHCO_3_. The mixture was extracted with EtOAc (×3) and the combined organic layers were washed with brine, dried over Na_2_SO_4_, filtered, and concentrated under reduced pressure. The residue was purified with PTLC (EtOAc:*n*-hexane = 3:7) to give methyl derivative **5** (5.2 mg, 68%) as a yellow solid. m.p. 113 °C; 1H-NMR (400 MHz, CDCl_3_) δ 10.89 (1H, s), 6.93 (1H, s), 5.59 (1H, br-s), 4.75 (1H, d, *J* = 2.7 Hz), 4.48 (1H, ddd, *J* = 2.7, 5.4, 8,3 Hz), 3.37 (3H, s), 2.25 (3H, s), 1.93 (1H, m), 1.84 (1H, m), 1.70-1.50 (1H, overlapped), 1.45 (1H, m), 1.40–1.25 (4H, overlapped), 0.91 (3H, t, *J* = 6.6 Hz); 13C-NMR (125 MHz, CDCl_3_) δ 169.4, 154.9, 144.9, 128.7, 125.8, 118.6, 106.6, 81.4, 69.8, 56.5, 31.6, 29.8, 24.9, 22.5, 15.8, 14.0.; IR (KBr) 3340, 2957, 2928, 2859, 1682, 1654, 1604, 1296, 1172 cm^−1^; HRMS (ESI) *m*/*z* (M + Na)^+^ calculated for (C_16_H_22_O_5_Na)^+^ 317.1365, found 317.1350.

#### 3.1.6. (3*S*,4*S*)-4-methoxy-5,8-bis(methoxymethoxy)-3-pentyl-7-vinylisochroman-1-one (**7a**)

To a solution of bromo compound **3** (200 mg, 0.447 mmol) and CsF (135.8 mg, 0.894 mmol) in degassed DMF (2.2 mL) were added tributylvinyltin (0.26 mL, 0.894 mmol) and PdCl_2_(PPh_3_)_2_ (62.8 mg, 89.0 µmol) at room temperature. After stirring for 1 h at 80 °C, the reaction was quenched by adding water. The mixture was extracted with EtOAc (×3) and the combined organic layers were washed with brine, dried over Na_2_SO_4_, filtered, and concentrated under reduced pressure. The residue was purified with flash column chromatography (EtOAc:*n*-hexane = 3:7) to give diMOM-protected vinyl derivative **7a** (185.1 mg, quant) as a yellow solid. m.p. 63–64 °C; 1H-NMR (500 MHz, CDCl_3_) δ 7.56 (1H, s), 7.14 (1H, dd, *J* = 11.1, 17.7 Hz), 5.76 (1H, d, *J* = 17.7 Hz), 5.40 (1H, d, *J* = 11.1 Hz), 5.24 (2H, s), 5.08 (1H, d, *J* = 6.3 Hz), 5.05 (1H, d, *J* = 6.3 Hz), 4.60 (1H, d, *J* = 1.3 Hz), 4.26 (1H, ddd, *J* = 1.3, 5.8, 7.4 Hz), 3.58 (3H, s), 3.50 (3H, s), 3.31 (3H, s), 2.03 (1H, m), 1.81 (1H, m), 1.56 (1H, m), 1.43 (1H, m), 1.40–1.25 (4H, overlapped), 0.90 (3H, t, *J* = 6.9 Hz); 13C-NMR (125 MHz, CDCl_3_) δ 162.0, 150.7, 150.2, 134.9, 131.3, 128.5, 119.7, 116.7, 116.0, 101.5, 95.2, 80.8, 68.3, 57.9, 56.8, 56.4, 31.6, 30.6, 24.9, 22.5, 14.0.; IR (KBr) 2953, 2931, 2861, 2829, 1730, 1471, 1426, 1155, 929 cm^−1^; HRMS (ESI) *m*/*z* (M + H)^+^ calculated for (C_21_H_31_O_7_)^+^ 395.2070, found 395.2078.

#### 3.1.7. (3*S*,4*S*)-5,8-dihydroxy-4-methoxy-3-pentyl-7-vinylisochroman-1-one (**7**)

To a solution of diMOM-protected methyl derivative **7a** (13.7 mg, 34.7 µmol) in MeOH (2.6 mL) was added 6 M aqueous HCl (0.87 mL) at 0 °C. After stirring for 3 h at 40 °C, the reaction was quenched by adding saturated aqueous NaHCO_3_. The mixture was extracted with EtOAc (×3) and the combined organic layers were washed with brine, dried over Na_2_SO_4_, filtered, and concentrated under reduced pressure. The residue was purified with PTLC (EtOAc:*n*-hexane = 3:7) to give vinyl derivative **7** (8.5 mg, 75%) as a yellow wax. 1H-NMR (500 MHz, CDCl_3_) δ 11.10 (1H, s), 7.23 (1H, s), 7.01 (1H, dd, *J* = 11.4, 17.7 Hz), 5.82 (1H, br-s), 5.80 (1H, d, *J* = 18.0 Hz), 5.37 (1H, d, *J* = 11.0 Hz), 4.77 (1H, br-s), 4.50 (1H, br-s), 3.40 (3H, s), 1.95 (1H, m), 1.85 (1H, m), 1.58 (1H, m), 1.45 (1H,m), 1.40–1.25 (4H, overlapped), 0.90 (3H, br-s); 13C-NMR (125 MHz, CDCl_3_) δ 169.3, 153.9, 145.4, 129.8, 128.0, 121.4, 120.9, 116.5, 107.7, 81.5, 69.7, 56.8, 31.6, 29.8, 24.9, 22.5, 14.0.; IR (KBr) 3311, 2956, 2930, 2859, 1659, 1438, 1171 cm^−1^; HRMS (ESI) *m*/*z* (M + Na)^+^ calculated for (C_17_H_22_O_5_Na)^+^ 329.1365, found 329.1368.

#### 3.1.8. (3*S*,4*S*)-4-methoxy-5,8-bis(methoxymethoxy)-3-pentyl-7-phenylisochroman-1-one (**9a**)

Bromo compound **3** (10.0 mg, 22.4 µmol), Cs_2_CO_3_ (21.9 mg, 67.1 µmol), phenylboronic acid (5.5 mg, 44.7 µM), and PdCl_2_(PPh_3_)_2_ (3.1 mg, 44.7 µmol) were dissolved in degassed dioxane (0.22 mL) at room temperature. After stirring for 1 h under reflux condition, the reaction was quenched by adding saturated aqueous NH_4_Cl. The mixture was extracted with EtOAc (×3) and the combined organic layers were washed with brine, dried over Na_2_SO_4_, filtered, and concentrated under reduced pressure. The residue was purified with flash column chromatography (EtOAc:*n*-hexane = 3:7) to give diMOM-protected phenyl derivative **9a** (7.4 mg, 75%) as a white wax. 1H-NMR (500 MHz, CDCl_3_) δ 7.55 (1H, d, *J* = 7.6 Hz), 7.50-7.38 (3H, overlapped), 7.36 (1H, dd, *J* = 7.3 Hz), 5.25 (2H, s), 4.80 (2H, s), 4.66 (1H, s), 4.33 (1H, t, *J* = 7.0 Hz), 3.50 (3H, s), 3.37 (3H, s), 2.92 (3H, s), 2.06 (1H, m), 1.85 (1H, m), 1.70–1.50 (1H, overlapped), 1.50–1.25 (5H, overlapped), 0.92 (3H, br-s); 13C-NMR (125 MHz, CDCl_3_) δ 162.1, 150.5, 150.0, 139.5, 137.9, 129.8, 128.3, 128.1, 127.7, 121.0, 119.9, 101.0, 95.1, 80.8, 68.3, 57.1, 56.4, 31.6, 30.6, 24.9, 22.5, 14.0.; IR (KBr) 2956, 2927, 2859, 2828, 1728, 1467, 1152, 1008, 932 cm^−1^; HRMS (ESI) *m*/*z* (M + Na)^+^ calculated for (C_25_H_32_O_7_Na)^+^ 467.2046, found 467.2043.

#### 3.1.9. (3*S*,4*S*)-5,8-dihydroxy-4-methoxy-3-pentyl-7-phenylisochroman-1-one (**9**)

To a solution of diMOM-protected methyl derivative **9a** (7.4 mg, 16.8 µmol) in THF (1.0 mL) was added 6 M aqueous HCl (0.50 mL) at 0 °C. After stirring for 6 h at room temperature, the reaction was quenched by adding saturated aqueous NaHCO_3_. The mixture was extracted with EtOAc (×3) and the combined organic layers were washed with brine, dried over Na_2_SO_4_, filtered, and concentrated under reduced pressure. The residue was purified with PTLC (EtOAc:*n*-hexane = 3:7) to give phenyl derivative **9** (6.0 mg, 90%) as a yellow solid. m.p. 173–174 °C; 1H-NMR (400 MHz, CDCl_3_) δ 11.21 (1H, s), 7.58 (2H, d, *J* = 7.3 Hz), 7.44 (2H, t, *J* = 7.3 Hz), 7.38 (1H, d, *J* = 7.6 Hz), 7.13 (1H, s), 5.76 (1H, br-s), 4.82 (1H, d, *J* = 2.7 Hz), 4.55 (1H, ddd, *J* = 2.7, 5.1, 8.3 Hz), 3.44 (3H, s), 1.98 (1H, m), 1.89 (1H, m), 1.70–1.40 (2H, overlapped), 1.40–1.25 (4H, overlapped), 0.92 (3H, t, *J* = 6.8 Hz); 13C-NMR (125 MHz, CDCl_3_) δ 169.5, 153.7, 145.4, 136.2, 131.8, 129.2, 128.3, 127.9, 125.5, 121.1, 107.8, 81.6, 69.6, 56.9, 31.6, 29.8, 24.9, 22.5, 14.0.; IR (KBr) 3307, 2955, 2928, 2859, 1650, 1425, 1295, 1194 cm^−1^; HRMS (ESI) *m*/*z* (M + H)^+^ calculated for (C_21_H_25_O_5_)^+^ 357.1702, found 357.1707.

#### 3.1.10. (3*S*,4*S*)-7-([1,1’-biphenyl]-4-yl)-4-methoxy-5,8-bis(methoxymethoxy)-3-pentylisochroman-1-one (**10a**)

Bromo compound **3** (20.0 mg, 44.7 µmol), Cs_2_CO_3_ (21.9 mg, 67.1 µmol), 4-biphenylboronic acid (5.5 mg, 44.7 µmol), and PdCl_2_(PPh_3_)_2_ (3.2 mg, 4.47 µmol) were dissolved in degassed dioxane (0.23 mL) at room temperature. After stirring for 1 h under reflux condition, the reaction was quenched by adding saturated aqueous NH_4_Cl. The mixture was extracted with EtOAc (×3) and the combined organic layers were washed with brine, dried over Na_2_SO_4_, filtered, and concentrated under reduced pressure. The residue was purified with PTLC (EtOAc:*n*-hexane = 3:7) to give diMOM-protected biphenyl derivative **10a** (18.0 mg, 88%) as a white solid. 1H-NMR (500 MHz, CDCl_3_) δ 7.74–7.60 (6H, overlapped), 7.53–7.40 (3H, overlapped), 7.38 (1H, t, *J* = 7.3 Hz), 5.28 (2H, s), 4.85 (1H, d, *J* = 7.0 Hz), 4.84 (1H, d, *J* = 7.0 Hz), 4.68 (1H, d, *J* = 1.3 Hz), 4.35 (1H, ddd, *J* = 1.3, 6.0, 7.6 Hz), 3.51 (3H, s), 3.38 (3H, s), 2.99 (3H, s), 2.08 (1H, m), 1.86 (1H, m), 1.70-1.50 (1H, overlapped), 1.46 (1H, m), 1.40–1.25 (4H, overlapped), 0.92 (3H, t, *J* = 6.9 Hz); 13C-NMR (125 MHz, CDCl_3_) δ 162.1, 150.6, 150.0, 140.5, 140.4, 139.1, 136.8, 130.2, 128.9, 128.1, 127.5, 127.0, 126.9, 120.9, 120.0, 101.1, 95.1, 80.8, 68.3, 57.2, 56.9, 56.4, 31.6, 30.6, 24.9, 22.5, 14.0.; IR (KBr) 2956, 2927, 2858, 2827, 1728, 1467, 1152, 1007, 931 cm^−1^; HRMS (ESI) *m*/*z* (M + H)^+^ calculated for (C_31_H_37_O_7_)^+^ 521.2539, found 521.2539.

#### 3.1.11. (3*S*,4*S*)-7-([1,1’-biphenyl]-4-yl)-5,8-dihydroxy-4-methoxy-3-pentylisochroman-1-one (**10**)

To a solution of diMOM-protected biphenyl derivative **10a** (12.9 mg, 24.8 µmol) in THF (1.7 mL) was added 6 M aqueous HCl (0.83 mL) at 0 °C. After stirring for 17 h at room temperature, the reaction was quenched by adding saturated aqueous NaHCO_3_ at 0 °C. The mixture was extracted with EtOAc (×3) and the combined organic layers were washed with brine, dried over Na_2_SO_4_, filtered, and concentrated under reduced pressure. The residue was purified with PTLC (EtOAc:*n*-hexane = 3:7) to give biphenyl derivative **10** (9.9 mg, 92%) as a yellow solid. m.p. 181–182 °C; 1H-NMR (400 MHz, CDCl_3_) δ 11.28 (1H, s), 7.67 (4H, s), 7.64 (2H, d, *J* = 7.3 Hz), 7.46 (2H, t, *J* = 7.3 Hz), 7.37 (1H, t, *J* = 7.3 Hz), 7.19 (1H, s), 5.75 (1H, br-s), 4.84 (1H, d, *J* = 2.7 Hz), 4.56 (1H, ddd, *J* = 2.7, 5.4, 8.3 Hz), 3.46 (3H, s), 1.98 (1H, m), 1.89 (1H, m), 1.70–1.50 (2H, overlapped), 1.45–1.25 (4H, overlapped), 0.92 (3H, t, *J* = 6.8 Hz); 13C-NMR (125 MHz, CDCl_3_) δ 169.4, 153.8, 145.5, 140.7, 135.2, 131.4, 129.6, 128.8, 127.5,127.15, 127.07, 125.3, 121.0, 107.9, 81.5, 69.8, 56.9, 31.6, 29.8, 24.9, 22.5, 14.0.; IR (KBr) 3283, 2954, 2929, 2863, 1668, 1595, 1295, 1220, 772 cm^−1^; HRMS (ESI) *m*/*z* (M + Na)^+^ calculated for (C_27_H_28_O_5_Na)^+^ 455.1834, found 455.1831.

#### 3.1.12. (3*S*,4*S*)-7-ethynyl-4-methoxy-5,8-bis(methoxymethoxy)-3-pentylisochroman-1-one (**8a**)

To a solution of aldehyde **12a** (5.4 mg, 13.6 µmol) in MeOH (0.14 mL) were added K_2_CO_3_ (5.7 mg, 40.9 µmol) and Ohira–Bestmann reagent (3.9 mg, 20.4 µmol) at room temperature. After stirring for 40 min at the same temperature, the mixture was concentrated under reduced pressure. The residue was purified with column chromatography (EtOAc:*n*-hexane = 1:4 to 1:1) to give diMOM alkyne derivative **8a** (6.3 mg, quant) as a yellow oil. 1H-NMR (500 MHz, CDCl_3_) δ 7.52 (1H, s), 5.27 (1H, d, *J* = 6.0 Hz), 5.22 (2H, s), 5.17 (1H, d, *J* = 6.0 Hz), 4.59 (1H, d, *J* = 1.3 Hz), 4.27 (1H, ddd, *J* = 1.3, 5.8, 7.4 Hz), 3.65 (3H, s), 3.49 (3H, s), 3.32 (3H, s), 2.05 (1H, m), 1.82 (1H, m), 1.65–1.50 (1H, overlapped), 1.42 (1H, m), 1.40–1.25 (4H, overlapped), 0.91 (3H, t, *J* = 7.1 Hz); 13C-NMR (125 MHz, CDCl_3_) δ 161.2, 154.6, 149.5, 130.0, 123.5, 120.4, 120.1, 101.0, 95.2, 82.7, 80.7, 79.3, 68.3, 58.1, 57.0, 56.5, 31.6, 30.5, 24.8, 22.5, 14.0.; IR (KBr) 3260, 2954, 2932, 2861, 2830, 1730, 1155, 1012, 931 cm^−1^; HRMS (ESI) *m*/*z* (M + H)^+^ calculated for (C_21_H_29_O_7_)^+^ 393.1913, found 393.1903.

#### 3.1.13. (3*S*,4*S*)-7-ethynyl-5,8-dihydroxy-4-methoxy-3-pentylisochroman-1-one (**8**)

To a solution of diMOM alkyne derivative **8a** (6.3 mg, 13.6 µmol) in MeOH (1.2 mL) was added 6 M aqueous HCl (0.40 mL) at room temperature. After stirring for 24 h at the same temperature, the reaction was quenched by adding saturated aqueous NaHCO_3_ at 0 °C. The mixture was extracted with EtOAc (×3) and the combined organic layers were washed with brine, dried over Na_2_SO_4_, filtered, and concentrated under reduced pressure. The residue was purified with column chromatography (EtOAc:*n*-hexane = 1:4 to 1:1) to give alkyne derivative **8** (3.3 mg, 67%) as a yellow solid. m.p. 132–133 °C; 1H-NMR (500 MHz, CDCl_3_) δ 11.20 (1H, s), 7.22 (1H, s), 6.03 (1H, br-s), 4.76 (1H, d, *J* = 2.5 Hz), 4.51 (1H, ddd, *J* = 2.5, 5.1, 8.2 Hz), 3.40 (3H, s), 3.39 (1H, s), 1.94 (1H, m), 1.84 (1H, m), 1.70–1.50 (1H, overlapped), 1.45 (1H, m), 1.40–1.25 (4H, overlapped), 0.91 (3H, t, *J* = 7.0 Hz); 13C-NMR (125 MHz, CDCl_3_) δ 168.6, 157.3, 145.1, 127.8, 123.4, 112.6, 108.0, 83.2, 81.4, 77.7, 69.7, 57.0, 31.5, 29.7, 24.8, 22.5, 14.0.; IR (KBr) 3294, 2956, 2930, 2859, 1679, 1434, 1172 cm^−1^; HRMS (ESI) *m*/*z* (M + H)^+^ calculated for (C_17_H_21_O_5_)^+^ 305.1389, found 305.1391.

#### 3.1.14. (3*S*,4*S*)-4-methoxy-5,8-bis(methoxymethoxy)-1-oxo-3-pentylisochromane-7-carbaldehyde (**12a**)

A stirred solution of **7a** (185.1 mg, 0.469 mmol) in CH_2_Cl_2_ (10.0 mL) was cooled to −78 °C and a stream of ozone was passed through it for 30 min. At this time, ozone gas was bubbled into the reaction mixture until the color of the reaction mixture turned to blue. After completion of the reaction, the mixture was purged with oxygen gas for 30 min before being treated with PPh_3_ (246.2 mg, 0.939 mmol) and allowed to warm to room temperature. After stirring at the same temperature for 12 h, the mixture was concentrated under reduced pressure and the resultant mixture was purified with column chromatography (EtOAc:*n*-hexane = 1:4 to 2:3) to give diMOM benzaldehyde derivative **12a** (177.4 mg, 95%) as a white solid. m.p. 38–39 °C; 1H-NMR (400 MHz, CDCl_3_) δ 10.42 (1H, s), 7.83 (1H, s), 5.29 (2H, s), 5.2 (2H, s), 4.65 (1H, d, *J* = 1.0 Hz), 4.29 (1H, *J* = 1.0, 5.6, 8.3 Hz), 3.59 (3H, s), 3.50 (3H, s), 3.35 (3H, s), 2.06 (1H, m), 1.83 (1H, m), 1.70-1.50 (1H, overlapped), 1.44 (1H, m), 1.40-1.30 (4H, overlapped), 0.91 (3H, t, *J* = 7.1 Hz); 13C-NMR (125 MHz, CDCl_3_) δ 189.9, 161.4, 156.6, 150.6, 135.8, 132.5, 120.8, 116.9, 103.0, 95.4, 81.0, 68.7, 58.4, 57.8, 57.0, 31.9, 30.8, 25.2, 22.8, 14.3.; IR (KBr) 2957, 2929, 2859, 2829, 1730, 1691, 1379, 1155, 930 cm^−1^; HRMS (ESI) *m*/*z* (M + H)^+^ calculated for (C_20_H_29_O_8_)^+^ 397.1862, found 397.1866.

#### 3.1.15. (3*S*,4*S*)-5,8-dihydroxy-4-methoxy-1-oxo-3-pentylisochromane-7-carbaldehyde (**12**)

To a solution of diMOM aldehyde derivative **12a** (10.0 mg, 25.2 µmol) in THF (1.9 mL) was added 6 M aqueous HCl (0.63 mL) at 0 °C. After stirring for 4 h at room temperature, the reaction was quenched by adding saturated aqueous NaHCO_3_ at 0 °C. The mixture was extracted with EtOAc (×3) and the combined organic layers were washed with brine, dried over Na_2_SO_4_, filtered, and concentrated under reduced pressure. The residue was purified with PTLC (EtOAc:*n*-hexane = 2:3) to give benzaldehyde derivative **12** (6.0 mg, 77%) as a pale yellow solid. m.p. 170 °C (dec); 1H-NMR (400 MHz, CDCl_3_) δ 11.33 (1H, s), 10.47 (1H, s), 7.70 (1H, d, *J* = 1.5 Hz), 6.62 (1H, br-s), 4.75 (1H, d, *J* = 2.2 Hz), 4.49 (1H, ddd, *J* = 2.2, 5.6, 8.0 Hz), 3.43 (3H, s), 2.03 (1H, s), 1.88 (1H, m), 1.61 (1H, m), 1.48 (1H, m), 1.42–1.30 (4H, overlapped), 0.92 (3H, t, *J* = 6.8 Hz); 13C-NMR (125 MHz, CDCl_3_) δ 189.0, 168.8, 158.9, 146.0, 131.3, 124.9, 121.5, 110.2, 82.2, 69.2, 57.9, 31.9, 30.3, 25.1, 22.8, 14.3.; IR (KBr) 3444, 3169, 2953, 2940, 2920, 1676, 1455, 1395, 1299 cm^−1^; HRMS (ESI) *m*/*z* (M + H)^+^ calculated for (C_16_H_21_O_6_)^+^ 309.1338, found 309.1342.

#### 3.1.16. (3*S*,4*S*)-7-(hydroxymethyl)-4-methoxy-5,8-bis(methoxymethoxy)-3-pentylisochroman-1-one (**11a**)

To a solution of diMOM aldehyde derivative **12a** (20.0 mg, 50.5 µmol) in MeOH (0.25 mL) was added NaBH_4_ (2.1 mg, 55.5 µmol) at 0 °C. After stirring for 15 min at the same temperature, the reaction was quenched by adding water at 0 °C. The mixture was extracted with EtOAc (×3) and the combined organic layers were washed with brine, dried over Na_2_SO_4_, filtered, and concentrated under reduced pressure. The residue was purified with PTLC (EtOAc:*n*-hexane = 1:1) to give diMOM hydroxymethyl derivative **11a** (18.6 mg, 93%) as a white wax. 1H-NMR (400 MHz, CDCl_3_) δ 7.46 (1H, s), 5.25 (1H, d, *J* = 6.8 Hz), 5.24 (1H, d, *J* = 6.8 Hz), 5.15 (2H, s), 4.72 (1H, dd, *J* = 6.4, 12.5 Hz), 4.62 (1H, d, *J* = 1.2 Hz), 4.58 (1H, dd, *J* = 7.8, 12.5 Hz), 4.25 (1H, ddd, *J* = 1.2, 5.8, 8.0 Hz), 3.64 (3H, s), 3.55 (1H, t, *J* = 6.8 Hz), 3.50 (3H, s), 3.31 (3H, s), 2.05 (1H, m), 1.83 (1H, m), 1.65-1.50 (1H, overlapped), 1.43 (1H, m), 1.42–1.30 (4H, overlapped), 0.91 (3H, t, *J* = 6.8 Hz); 13C-NMR (125 MHz, CDCl_3_) δ 162.4, 152.7, 150.7, 138.7, 128.9, 120.8, 119.3, 102.2, 95.4, 81.2, 68.4, 61.4, 57.8, 57.2, 56.8, 31.9, 30.9, 25.2, 22.9, 14.4.; IR (KBr) 3443, 2957, 2928, 2859, 2828, 1724, 1153, 1012 cm^−1^; HRMS (ESI) *m*/*z* (M + H)^+^ calculated for (C_20_H_31_O_8_)+ 399.2019, found 399.2017.

#### 3.1.17. (3*S*,4*S*)-5,8-dihydroxy-7-(hydroxymethyl)-4-methoxy-3-pentylisochroman-1-one (**11**)

To a solution of diMOM hydroxymethyl derivative **11a** (7.2 mg, 24.1 µmol) in MeOH (1.8 mL) was added 6 M aqueous HCl (0.45 mL) at 0 °C. After stirring for 4 h at 40 °C, the reaction was quenched by adding saturated aqueous NaHCO_3_ at 0 °C. The mixture was extracted with EtOAc (×3) and the combined organic layers were washed with brine, dried over Na_2_SO_4_, filtered, and concentrated under reduced pressure. The residue was purified with PTLC (EtOAc:*n*-hexane = 1:1) to give hydroxymethyl derivative **11** (3.9 mg, 52%) as a white solid. m.p. 143–145 °C; 1H-NMR (400 MHz, CDCl_3_) δ 10.99 (1H, s), 7.12 (1H, s), 6.03 (1H, br-s), 4.74 (1H, d, *J* = 2.4 Hz), 4.72 (2H, br-s), 4.48 (1H, ddd, *J* = 2.4, 5.2, 8.0 Hz), 3.38 (3H, s), 2.53 (1H, br-s), 1.96 (1H, m), 1.86 (1H, m), 1.70–1.50 (1H, overlapped), 1.46 (1H, m), 1.40–1.25 (4H, overlapped), 0.91 (3H, t, *J* = 6.8 Hz); 13C-NMR (125 MHz, CDCl_3_) δ 169.5, 154.2, 145.8, 130.8, 123.8, 121.4, 107.8, 82.1, 69.8, 61.2, 57.2, 31.9, 30.2, 25.2, 22.8, 14.3.; IR (KBr) 2951, 2921, 2854, 1682, 1440, 1302 cm^−1^; HRMS (ESI) *m*/*z* (M + H)^+^ calculated for (C_16_H_23_O_6_)^+^ 311.1495, found 311.1498.

#### 3.1.18. ((3*S*,4*S*)-4-methoxy-5,8-bis(methoxymethoxy)-1-oxo-3-pentylisochroman-7-yl)methylmethanesulfonate (**22**)

To a solution of diMOM hydroxymethyl derivative **11a** (7.2 mg, 24.1 µmol) in CH_2_Cl_2_ (0.47 mL) were added Et_3_N (10.8 µL, 77.5 µmol) and MsCl (6.0 µL, 77.5 µmol) at 0 °C. After stirring for 40 min at the same temperature, the reaction was quenched by adding water at 0 °C. The mixture was extracted with EtOAc (×3) and the combined organic layers were washed with brine, dried over Na_2_SO_4_, filtered, and concentrated under reduced pressure. The residue was purified with PTLC (EtOAc:*n*-hexane = 2:3) to give diMOM mesylated derivative **22** (30.4 mg, 91%) as a white wax. 1H-NMR (400 MHz, CDCl_3_) δ 7.51 (1H, s), 5.45 (1H, d, *J* = 12.0 Hz), 5.37 (1H, d, *J* = 12.2 Hz), 5.25 (2H, s), 5.14 (1H, d, *J* = 6.6 Hz), 5.12 (1H, d, *J* = 6.6 Hz), 4.62 (1H, d, *J* = 1.4 Hz), 4.27 (1H, ddd, *J* = 1.2, 5.6, 7.8 Hz), 3.59 (3H, s), 3.50 (3H, s), 3.33 (3H, s), 3.07 (3H, s), 2.03 (1H, m), 1.82 (1H, m), 1.58 (1H, m), 1.44 (1H, m), 1.40–1.25 (4H, overlapped), 0.91 (3H, t, *J* = 6.8 Hz); 13C-NMR (100 MHz, CDCl_3_) δ 161.9, 152.2, 150.5, 131.3, 130.5, 120.3, 119.7, 102.8, 95.5, 81.2, 68.6, 66.9, 58.1, 57.4, 56.9, 38.2, 31.9, 30.9, 25.2, 22.8, 14.3.; IR (KBr) 2958, 2930, 2860, 1829, 1681, 1440, 1358, 1175, 933 cm^−1^; HRMS (ESI) *m*/*z* (M + Na)^+^ calculated for (C_21_H_32_O_10_SNa)^+^ 499.1614, found 499.1616.

#### 3.1.19. (3*S*,4*S*)-7-(azidomethyl)-4-methoxy-5,8-bis(methoxymethoxy)-3-pentylisochroman-1-one (**13a**)

To a solution of diMOM mesylated derivative **22** (5.3 mg, 11.1 µmol) in DMF (55 µL) was added NaN_3_ (0.79 mg, 12.1 µmol) at room temperature. After stirring for 6 h at the same temperature, the reaction was quenched by adding water at 0 °C. The mixture was extracted with EtOAc (×3) and the combined organic layers were washed with brine, dried over Na_2_SO_4_, filtered, and concentrated under reduced pressure. The residue was purified with PTLC (EtOAc:*n*-hexane = 3:7) to give diMOM azide derivative **13a** (3.7 mg, 79%) as a pale-yellow oil. 1H-NMR (500 MHz, CDCl_3_) δ 7.44 (1H, s), 5.26 (1H, d, *J* = 6.9 Hz), 5.25 (1H, d, *J* = 6.9 Hz), 5.13 (1H, d, *J* = 6.9 Hz), 5.11 (1H, d, *J* = 6.9 Hz), 4.65 (1H, d, *J* = 14.5 Hz), 4.62 (1H, d, *J* = 1.3 Hz), 4.53 (1H, d, *J* = 14.5 Hz), 4.27 (1H, ddd, *J* = 1.3, 5.7, 7.3 Hz), 3.60 (3H, s), 3.51 (3H, s), 3.32 (3H, s), 2.04 (1H, m), 1.82 (1H, m), 1.65–1.50 (1H, overlapped), 1.43 (1H, m), 1.40–1.30 (4H, overlapped), 0.91 (3H, t, *J* = 7.0 Hz); 13C-NMR (125 MHz, CDCl_3_) δ 162.2, 152.1, 150.5, 133.5, 129.1, 119.7, 119.5, 102.6, 95.5, 81.2, 68.6, 57.9, 57.3, 56.8, 50.2, 31.9, 30.9, 25.2, 22.9, 14.4.; IR (KBr) 2957, 2928, 2858, 2829, 2105, 1729, 1153, 1009 cm^−1^; HRMS (ESI) *m*/*z* (M + H)^+^ calculated for (C_20_H_30_N_3_O_7_)^+^ 424.2084, found 424.2085.

#### 3.1.20. (3*S*,4*S*)-7-(azidomethyl)-5,8-dihydroxy-4-methoxy-3-pentylisochroman-1-one (**13**)

To a solution of diMOM azide derivative **13a** (8.3 mg, 19.6 µmol) in MeOH (1.5 mL) was added 6 M aqueous HCl (0.49 mL) at room temperature. After stirring for 4 h at 40 °C, the reaction was quenched by adding saturated aqueous NaHCO_3_ at 0 °C. The mixture was extracted with EtOAc (×3) and the combined organic layers were washed with brine, dried over Na_2_SO_4_, filtered, and concentrated under reduced pressure. The residue was purified with PTLC (EtOAc:*n*-hexane = 3:7) to give nitro derivative **13** (3.1 mg, 49%) as a white solid. m.p. 98–99 °C; 1H-NMR (400 MHz, CDCl_3_) δ 10.98 (1H, s), 7.10 (1H, s), 5.81 (1H, br-s), 4.78 (1H, d, *J* = 2.9 Hz), 4.52 (1H, ddd, *J* = 2.9, 5.4, 8.5 Hz), 4.45 (1H, d, *J* = 14.4 Hz), 4.42 (1H, d, *J* = 14.4 Hz), 3.41 (3H, s), 1.93 (1H, m), 1.86 (1H, m), 1.70-1.50 (1H, overlapped), 1.47 (1H, m), 1.40–1.25 (4H, overlapped), 0.91 (3H, t, *J* = 7.1 Hz); 13C-NMR (125 MHz, CDCl_3_) δ 169.1, 154.4, 145.7, 126.2, 124.6, 121.8, 108.0, 81.7, 70.4, 57.2, 49.3, 31.9, 30.0, 25.2, 22.8, 14.3.; IR (KBr) 2959, 2924, 2857, 2108, 1654, 1441, 1293, 1170 cm^−1^; HRMS (ESI) *m*/*z* (M + H)^+^ calculated for (C_16_H_22_N_3_O_5_)^+^ 336.1559, found 336.1563.

#### 3.1.21. (3*S*,4*S*)-7-(aminomethyl)-4-methoxy-5,8-bis(methoxymethoxy)-3-pentylisochroman-1-one (**14a**)

To a solution of diMOM azide derivative **13a** (3.3 mg, 7.8 µmol) in MeOH (0.78 mL) was added Et_3_N (0.10 mL, 7.35 mmol) and Pd/C (1.6 mg, 1.5 µmol) at room temperature. After stirring for 1 h at the same temperature, the mixture was filtered, and the filtrate was concentrated under reduced pressure. The residue was purified with PTLC (MeOH:CH_2_Cl_2_ = 1:9) to give diMOM amine derivative **14a** (2.0 mg, 65%) as brown oil. 1H-NMR (400 MHz, CDCl_3_) δ 7.49 (1H, s), 5.26 (2H, s), 5.16 (1H, d, *J* = 7.2 Hz), 5.07 (1H, d, *J* = 6.8 Hz), 4.61 (1H, d, *J* = 1.2 Hz), 4.27 (1H, ddd, *J* = 1.2, 6.0, 7.6 Hz), 4.00 (2H, s), 3.61 (3H, s), 3.50 (3H, s), 3.32 (3H, s), 2.59 (1H, br-s), 2.03 (1H, m), 1.82 (1H, m), 1.57 (1H, m), 1.43 (1H, m), 1.40-1.25 (1H, overlapped), 0.91 (3H, t, *J* = 6.8 Hz); 13C-NMR (125 MHz, CDCl_3_) δ 162.6, 152.6, 150.5, 128.1, 120.0, 119.0, 102.4, 95.4, 81.2, 68.5, 57.9, 57.2, 56.8, 42.5, 32.0, 30.9, 30.0, 25.2, 22.9, 14.4.; IR (KBr) 2957, 2925, 2857, 2827, 1726, 1470, 1153, 1005 cm^−1^; HRMS (ESI) *m*/*z* (M + H)^+^ calculated for (C_20_H_32_NO_7_)^+^ 398.2179, found 398.2178.

#### 3.1.22. (3*S*,4*S*)-7-(aminomethyl)-5,8-dihydroxy-4-methoxy-3-pentylisochroman-1-one (**14**)

To a solution of diMOM amine derivative **14a** (4.4 mg, 11.1 µmol) in MeOH (0.83 mL) was added 6 M aqueous HCl (0.28 mL) at 0 °C. After stirring for 5 h at room temperature, the reaction was quenched by adding saturated aqueous NaHCO_3_ at 0 °C. The mixture was extracted with the mixture of MeOH and CH_2_Cl_2_ (MeOH:CH_2_Cl_2_ = 1:4) (×4) and the combined organic layers were dried over Na_2_SO_4_, filtered and concentrated under reduced pressure. The residue was purified with PTLC (MeOH:CHCl_3_ saturated with NH_3_ = 1:9) to give amiomethyl derivative **14** (1.1 mg, 32%) as brown solid. m.p. 78–80 °C; 1H-NMR (400 MHz, CDCl_3_) δ 6.98 (1H, s), 4.59 (1H, d, *J* = 1.8 Hz), 4.35 (1H, ddd, *J* = 1.8, 6.0, 8.0 Hz), 3.97 (1H, d, *J* = 13.3 Hz), 3.88 (1H, d, *J* = 13.3 Hz), 3.19 (3H, s), 1.98 (1H, m), 1.83 (1H, m), 1.56 (1H, m), 1.43 (1H, m), 1.40–1.25 (4H, overlapped), 0.90 (3H, t, *J* = 7.0 Hz) ; 13C-NMR (125 MHz, CDCl_3_) δ 169.9, 154.2, 146.2, 130.0, 125.8, 122.9, 108.1, 82.8, 68.5, 56.9, 42.3, 31.9, 30.6, 25.1, 22.8, 14,3.; IR (KBr) 2956, 2921, 2857, 1676, 1441, 1171 cm^−1^; HRMS (ESI) *m*/*z* (M + Na)^+^ calculated for (C_16_H_23_NO_5_Na)^+^ 332.1474, found 332.1474.

#### 3.1.23. ((3*S*,4*S*)-5,8-dihydroxy-4-methoxy-7-nitro-3-pentylisochroman-1-one (**15**)

To a solution of **3** (28.9 mg, 89.1 µmol) in AcOH (0.50 mL) was added the mixture of AcOH and 70% HNO_3_ (0.80 mL:0.20 mL) at 0 °C. After stirring for 10 min at the same temperature, the reaction was quenched by adding saturated aqueous NaHCO_3_ at 0 °C. The mixture was extracted with EtOAc (×3) and the combined organic layers were washed with saturated aqueous NaHCO_3_ and brine, dried over Na_2_SO_4_, filtered, and concentrated under reduced pressure. The residue was pathed through SiO_2_ plug and the resultant mixture of monoMOM nitro derivative **15a** was used for the next reaction without further purification. To a solution of **15a** mixture in MeOH (7.5 mL) was added 6 M aqueous HCl (2.4 mL) at 0 °C. After stirring for 5 h at 40 °C, the reaction was quenched by adding saturated aqueous NaHCO_3_ at 0 °C. The mixture was extracted with EtOAc (×3) and the combined organic layers were washed with brine, dried over Na_2_SO_4_, filtered, and concentrated under reduced pressure. The residue was purified with PTLC (EtOAc:*n*-hexane = 1:1) to give nitro derivative **15** (21.5 mg, 74%) as a yellow solid. m.p. 158-159; 1H-NMR (400 MHz, CDCl_3_) δ 11.89 (1H, s), 7.78 (1H, s), 6.80 (1H, br-s), 4.82 (1H, d, *J* = 2.6 Hz), 4.55 (1H, ddd, *J* = 2.6, 5.2, 8.3 Hz), 3.46 (3H, s), 1.96 (1H, m), 1.86 (1H, m), 1.59 (1H, m), 1.47 (1H, m), 1.40–1.25 (4H, overlapped), 0.91 (3H, t, *J* = 7.1 Hz); 13C-NMR (125 MHz, CDCl_3_) δ 167.5, 150.4, 144.9, 137.6, 129.4, 119.7, 110.7, 81.0, 70.3, 57.6 , 31.4, 29.4, 24.7, 22.4, 14.0; IR (KBr) 3416, 2962, 2927, 2857, 1679, 1445, 1261, 1018, 800 cm^−1^; HRMS (ESI) *m*/*z* (M + H)^+^ calculated for (C_15_H_20_NO_7_)^+^ 326.1240, found 326.1224.

#### 3.1.24. (3*S*,4*S*)-7-amino-5,8-dihydroxy-4-methoxy-3-pentylisochroman-1-one (**16**)

To a solution of nitro derivative **15** (5.0 mg, 15.4 µmol) in THF (0.62 mL) and MeOH (80 µL) was added PtO_2_ (0.3 mg, 1.54 µmol) at room temperature. After stirring for 1.5 h at the same temperature under hydrogen atmosphere (1 atm), the mixture was passed through a membrane filter to remove PtO_2_. The mixture was concentrated under reduced pressure and the residue was purified with PTLC (EtOAc:*n*-hexane = 3:7, developed by three times) to give nitro derivative **16** (4.3 mg, 95%) as a yellow solid. m.p. 118–119 °C; 1H-NMR (500 MHz, CDCl_3_) δ 10.72 (1H, s), 6.45 (1H, s), 5.68 (1H, br-s), 4.67 (1H, d, *J* = 2.5 Hz), 4.46 (1H, ddd, *J* = 2.5, 5.5, 8.3 Hz), 4.05 (1H, br-s), 3.32 (3H, s), 1.94 (1H, m), 1.84 (1H, m), 1.75–1.50 (1H, overlapped), 1.45 (1H, m), 1.40–1.25 (4H, overlapped), 0.90 (3H, t, *J* = 7.0 Hz); 13C-NMR (125 MHz, CDCl_3_) δ 169.8, 145.9, 144.5, 137.2, 109.8, 108.4, 106.8, 82.4, 69.1, 56.1, 31.6, 30.1, 24.9, 22.5, 14.0; IR (KBr) 3378, 2957, 2926, 2858, 1681, 1464, 1217, 1171 cm^−1^; HRMS (ESI) *m*/*z* (M + Na)^+^ calculated for (C_15_H_21_NO_5_Na)^+^ 318.1317, found 318.1321.

#### 3.1.25. (3*S*,4*S*)-7-chloro-8-hydroxy-4-methoxy-5-(methoxymethoxy)-3-pentylisochroman-1-one (**18a**)

To a solution of **3** (5.0 mg, 15.4 µmol) in DMF (0.18 mL) was added the solution of *N*-chlorosuccinimide (4.1 mg, 30.8 µmol) in DMF (31 µL) at room temperature. After stirring for 5 h at 65 °C, the reaction was quenched by adding saturated aqueous NaHCO_3_ at 0 °C. The mixture was extracted with EtOAc (×3) and the combined organic layers were washed with brine, dried over Na_2_SO_4_, filtered, and concentrated under reduced pressure. The residue was purified with PTLC (EtOAc:*n*-hexane = 1:9) to give monoMOM chloro derivative **18a** (3.3 mg, 60%) as a brown solid. m.p. 79–81 °C; 1H-NMR (400 MHz, CDCl_3_) δ 11.23 (1H, s), 7.55 (1H, s), 5.18 (1H, d, *J* = 7.0 Hz), 5.16 (1H, d, *J* = 7.0 Hz), 4.59 (1H, d, *J* = 1.7 Hz), 4.39 (1H, ddd, *J* = 1.7, 6.0, 8.0 Hz), 3.50 (3H, s), 3.30 (3H, s), 2.07 (1H, m), 1.86 (1H, m), 1.70-1.50 (1H, overlapped), 1.47 (1H, m), 1.45-1.25 (4H, overlapped), 0.92 (3H, t, *J* = 7.1 Hz); 13C-NMR (125 MHz, CDCl_3_) δ 168.7, 152.8, 146.3, 125.1, 123.6, 123.0, 109.0, 95.7, 82.7, 67.4, 56.8, 56.4, 31.5, 30.4, 24.7, 22.5, 14.0.; IR (KBr) 2955, 2927, 2853, 2826, 1681, 1453, 1433, 1206 cm^−1^; HRMS (ESI) *m*/*z* (M + Na)^+^ calculated for (C_17_H_23_O_6_ClNa)^+^ 381.1081, found 381.1088.

#### 3.1.26. (3*S*,4*S*)-7-chloro-5,8-dihydroxy-4-methoxy-3-pentylisochroman-1-one (**18**)

To a solution of monoMOM chloro derivative **18a** (3.3 mg, 9.20 µmol) in MeOH (0.69 mL) was added 6 M aqueous HCl (0.23 mL) at 0 °C. After stirring for 2 h at 40 °C, the reaction was quenched by adding saturated NaHCO_3_ at 0 °C. The mixture was extracted with EtOAc (×3) and the combined organic layers were washed with brine, dried over Na_2_SO_4_, filtered, and concentrated under reduced pressure. The residue was purified with PTLC (EtOAc:*n*-hexane = 1:9) to give chloro derivative **18** (2.1 mg, 73%) as a brown solid. m.p. 119-120 °C; 1H-NMR (400 MHz, CDCl_3_) δ 11.17 (1H, br-s), 7.34 (1H, s), 6.34 (1H, br-s), 4.82 (1H, br-s), 4.59 (1H, ddd, *J* = 2.8, 5.6, 8.4 Hz), 3.48 (3H, s), 2.03 (1H, m), 1.93 (1H, m), 1.64 (1H, m), 1.53 (1H, m), 1.51–1.35 (4H, overlapped), 0.98 (3H, t, *J* = 7.2 Hz); 13C-NMR (100 MHz, CDCl_3_) δ 168.7, 152.1, 145.6, 124.9, 122.8, 121.1, 108.5, 81.8, 69.6, 57.0, 31.5, 29.8, 24.8, 22.5, 14.0.; IR (KBr) 3282, 2958, 2929, 2860, 1681, 1437, 1198 cm^−1^; HRMS (ESI) *m*/*z* (M + H)^+^ calculated for (C_15_H_20_O_5_Cl)^+^ 315.0999, found 315.0998.

#### 3.1.27. (3*S*,4*S*)-7-bromo-5,8-dihydroxy-4-methoxy-3-pentylisochroman-1-one (**19**)

To a solution of bromo derivative **2** (11.0 mg, 24.6 µmol) in MeOH (1.8 mL) was added 6 M aqueous HCl (0.62 mL) at 0 °C. After stirring for 3.5 h at 40 °C, the reaction was quenched by adding saturated aqueous NaHCO_3_ at 0 °C. The mixture was extracted with EtOAc (×3) and the combined organic layers were washed with brine, dried over Na_2_SO_4_, filtered, and concentrated under reduced pressure. The residue was purified with PTLC (EtOAc:*n*-hexane = 1:9) to give bromo derivative **19** (8.6 mg, 97%) as a white solid. m.p. 132 °C; 1H-NMR (400 MHz, CDCl_3_) δ 11.26 (1H, s), 7.36 (1H, s), 6.00 (1H, br-s), 4.76 (1H, d, *J* = 2.7 Hz), 4.52 (1H, ddd, *J* = 2.7, 5.1, 8.3 Hz), 3.41 (3H, s), 1.95 (1H, m), 1.86 (1H, m), 1.70-1.50 (2H, overlapped), 1.40–1.25 (4H, overlapped), 0.91 (3H, t, *J* = 7.0 Hz); 13C-NMR (125 MHz, CDCl_3_) δ 168.4, 153.0, 145.8, 127.9, 121.5, 111.6, 108.2, 81.4, 70.0, 57.0, 31.5, 29.6, 24.8, 22.5, 14.0.; IR (KBr) 3296, 2955, 2930, 2859, 1679, 1432, 1197 cm^−1^; HRMS (ESI) *m*/*z* (M + Na)^+^ calculated for (C_15_H_19_O_5_BrNa)^+^ 381.0314, found 381.0322.

#### 3.1.28. (3*S*,4*S*)-5,8-dihydroxy-7-iodo-4-methoxy-3-pentylisochroman-1-one (**20**)

To a solution of **3** (12.6 mg, 38.8 µmol) in DMF (0.35 mL) was added the solution of *N*-iodosuccinimide (17.5 mg, 77.6 µmol) in DMF (50 µL) at room temperature. After stirring for 3 h at room temperature, the reaction was quenched by adding saturated aqueous NaHCO_3_ at 0 °C. The mixture was extracted with CH_2_Cl_2_ (×3) and the combined organic layers were washed with brine, dried over Na_2_SO_4_, filtered, and concentrated under reduced pressure. The residue was pathed through SiO_2_ plug and the resultant mixture of monoMOM iodo derivative **20a** was used for the next reaction without further purification. To a solution of crude mixture of **20a** in MeOH (0.83 mL) was added 6 M aqueous HCl (0.30 mL) at 0 °C. After stirring for 5 h at 40 °C, the reaction was quenched by adding saturated aqueous NaHCO_3_ at 0 °C. The mixture was extracted with EtOAc (×3) and the combined organic layers were washed with brine, dried over Na_2_SO_4_, filtered, and concentrated under reduced pressure. The residue was purified with PTLC (EtOAc:*n*-hexane = 1:9) to give iodo derivative **20** (4.0 mg, 87%) as a pale-yellow oil. m.p. 109–110 °C; 1H-NMR (500 MHz, CDCl_3_) δ 11.44 (1H, s), 7.57 (1H, s), 6.11 (1H, br-s), 4.51 (1H, ddd, *J* = 2.8, 5.4, 8.5 Hz), 3.40 (3H, s), 1.94 (1H, m), 1.85 (1H, m), 1.75–1.50 (4H, overlapped), 1.45 (1H, m), 1.40–1.30 (4H, overlapped), 0.91 (3H, t, *J* = 7.0 Hz); 13C-NMR (125 MHz, CDCl_3_) δ 168.3, 155.3, 146.3, 133.8, 122.6, 107.1, 85.5, 81.5, 69.8, 56.9, 31.5, 29.7, 24.8, 22.5, 14.0; IR (KBr) 3293, 2977, 298, 2857, 1674, 1427, 1197 cm^−1^; HRMS (ESI) *m*/*z* (M + Na)^+^ calculated for (C_15_H_19_O_5_Ina)^+^ 429.0175, found 429.0174.

### 3.2. Bactericidal Assay

Methicillin-susceptible *Staphylococcus aureus* (MSSA) ATCC25923 and methicillin-resistant *Staphylococcus aureus* (MRSA) ATCC 33,591 were aerobically incubated at 37 °C in Luria–Bertani medium (LB, Nippon Becton Dickinson Company, Tokyo, Japan). *Porphyromonas gingivalis* W83 was anaerobically incubated at 37 °C in Gifu anaerobic medium (GAM, Nissui, Tokyo, Japan). Each culture (20 µL) prepared to an optical density of 1.5 at 600 nm were appropriately incubated with various concentrations of synthesized compounds in 200 µL of culture medium at 37 °C for 24 h in 96-well plate (Thermo scientific, MA, USA). Compounds were dissolved in DMSO (Wako, Osaka, Japan). The degree of turbidity in the broth culture was measured at absorbance 600 nm using microplate reader (Thermo scientific, MA, USA).

### 3.3. Cellular Toxicity

Human lung adenocarcinoma epithelial cell line A549 cells were cultured at 37 °C in growth medium (DMEM with 10% fetal bovine serum) in 5% CO_2_, and then seeded into 96-well plates at a density of 1 × 10^5^ cells/mL. Once the cells reached 80%–90% confluence, they were treated with or without 10 µM of various compounds at 37 °C for 12 h. Next, 10 μL Cell Counting Kit-8 (Dojindo Molecular Technologies, Kumamoto, Japan) solution was added to each well, and the plate was incubated for 2 h at 37 °C. Cell viability was determined by measuring the absorbance at 450 nm using a fluorimeter (Varioscan, Thermo, USA).

## 4. Conclusions

We constructed a chemical library of the side-chain derivatives of eurotiumide A, which is a dihydroisocoumarin-type marine natural product. The antimicrobial evaluation of these compounds was conducted against MSSA, MRSA, and *P. gingivalis*. We discovered several compounds to be effective against these strains; among them, the isopentyl derivative **6** is especially more active against all three strains than **1**. Continuous research to clarify the modes of action of these derivatives is under way in our laboratory.

## Figures and Tables

**Table 1:** The IC_50_ values (µM) of the selected side chain derivatives against methicillin-susceptible *S. aureus* (MSSA), methicillin-resistant *S. aureus* (MRSA), and *P. gingivalis*. Vancomycin (VCM) was used as a positive control against MSSA and MRSA. Cefcapene pivoxyl (CFPN-PI) was used as a positive control against *P. gingivalis*.

Strains	1	6	7	16	18	19	20	VCM	CFPN-PI
Methicillin-susceptible *S. aureus* (MSSA)	–	5.6	–	–	–	–	9.0	1.3	–
Methicillin-resistant *S. aureus* (MRSA)	–	4.3	–	–	–	–	–	1.5	–
*P. gingivalis*	3.6	2.0	3.5	6.7	6.4	7.0	3.5	–	0.03

## Supplementary Materials

The following are available online at https://www.mdpi.com/1660-3397/18/2/92/s1, 1H- and 13C-NMR charts of all new compounds.
