# Supplementary material for: Synthesis and Antimicrobial Evaluation of Side-Chain Derivatives based on Eurotiumide A
Source: Mar Drugs. 2020 Jan 30;18(2):92. doi: 10.3390/md18020092 (PMC7074549; doi:10.3390/md18020092)

Supplementary material

Synthesis and Antimicrobial Evaluation of Side-Chain Derivatives based on Eurotiumide A

Atsushi Nakayama[a]*, Hideo Sato[a], Tenta Nakamura[a], Mai Hamada[a], Shuji Nagano[a], Shuhei Kameyama[a], Yui Furue[b], Naoki Hayashi[b], Go Kamoshida[b], Sangita Karanjit[a], Masataka Oda[b], and Kosuke Namba[a]*

[1] Graduate School of Pharmaceutical Sciences and Research Cluster on "Innovative Chemical Sensing", Tokushima University, 1-78-1 Shomachi, Tokushima 770-8505, Japan; anakaya@tokushima-u.ac.jp

[2] Department of Microbiology and Infection Control Sciences, Kyoto Pharmaceutical University, Misasaginakauchi-cho, Yamashita-Ku, Kyoto 607-8414, Japan; e-mail@e-mail.com

* Correspondence: anakaya@tokushima-u.ac.jp (A.N.) & namba@tokushima-u.ac.jp (K.N.)

Table of contents

· **1H-NMR and 13C-NMR spectra of synthetic compounds S2 – S28**

Compound 4

^{1}H-NMR ($CDCl_3$, 400 MHz)

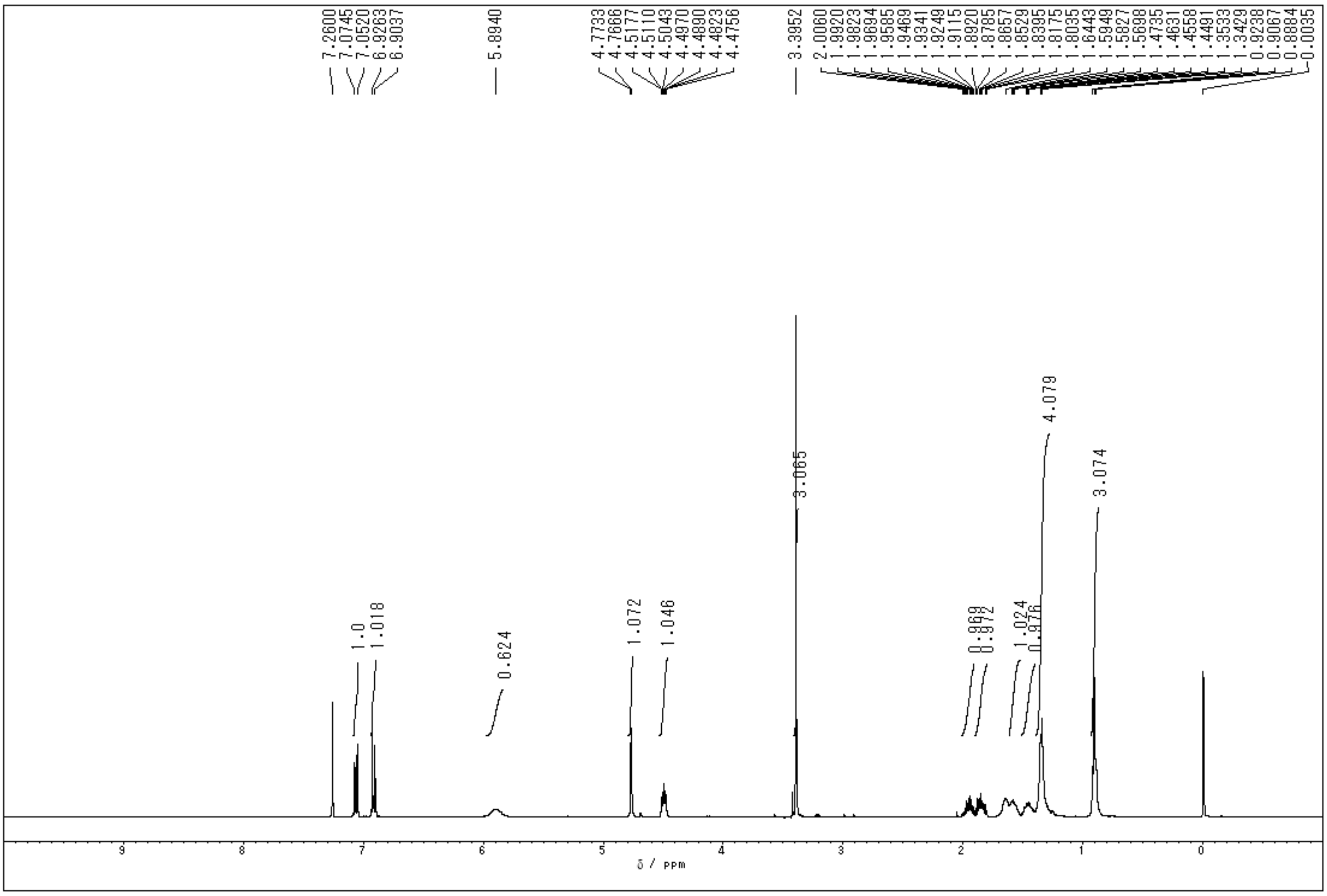

^{13}C-NMR ($CDCl_3$, 100 MHz)

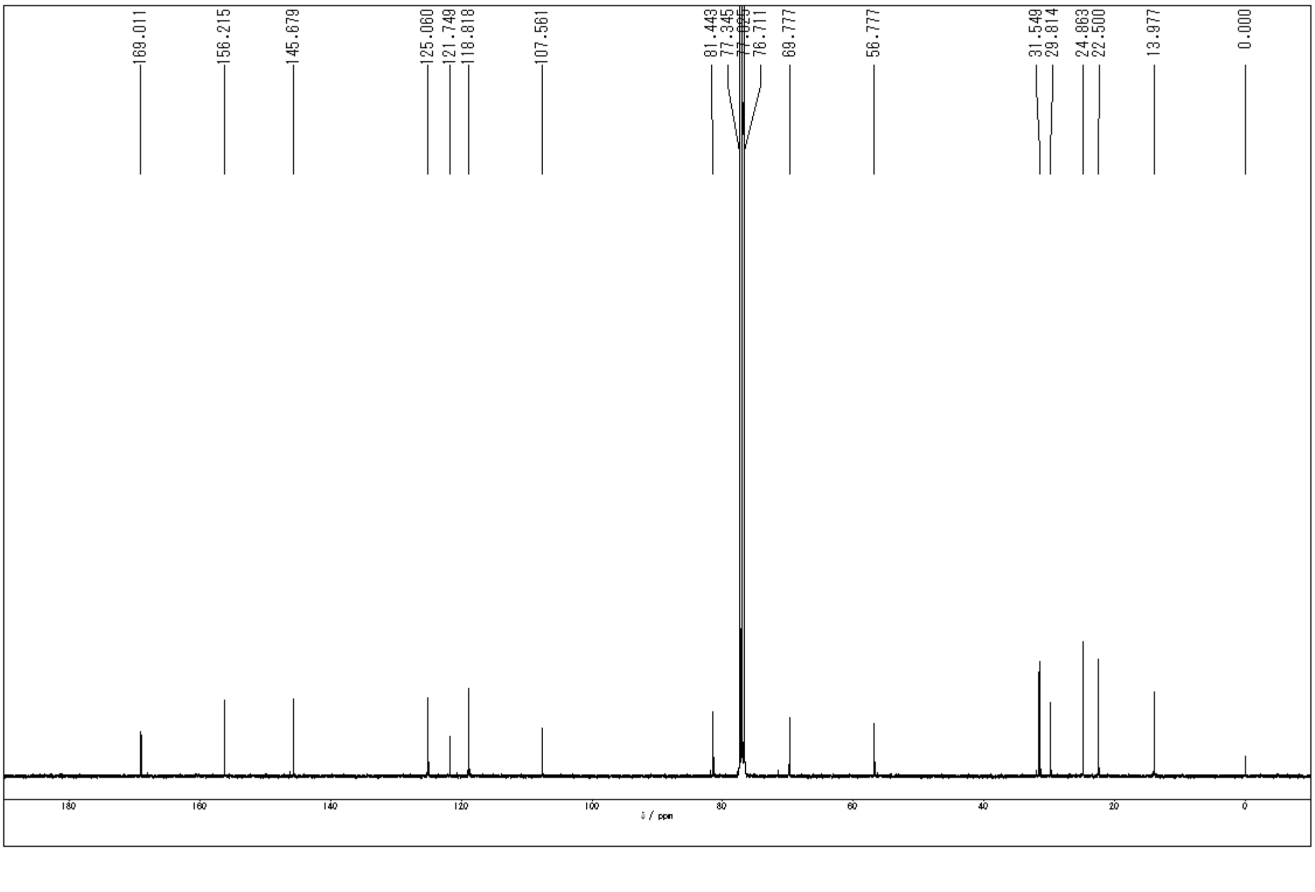

Compound 5a

^{1}H-NMR ($CDCl_3$, 400 MHz)

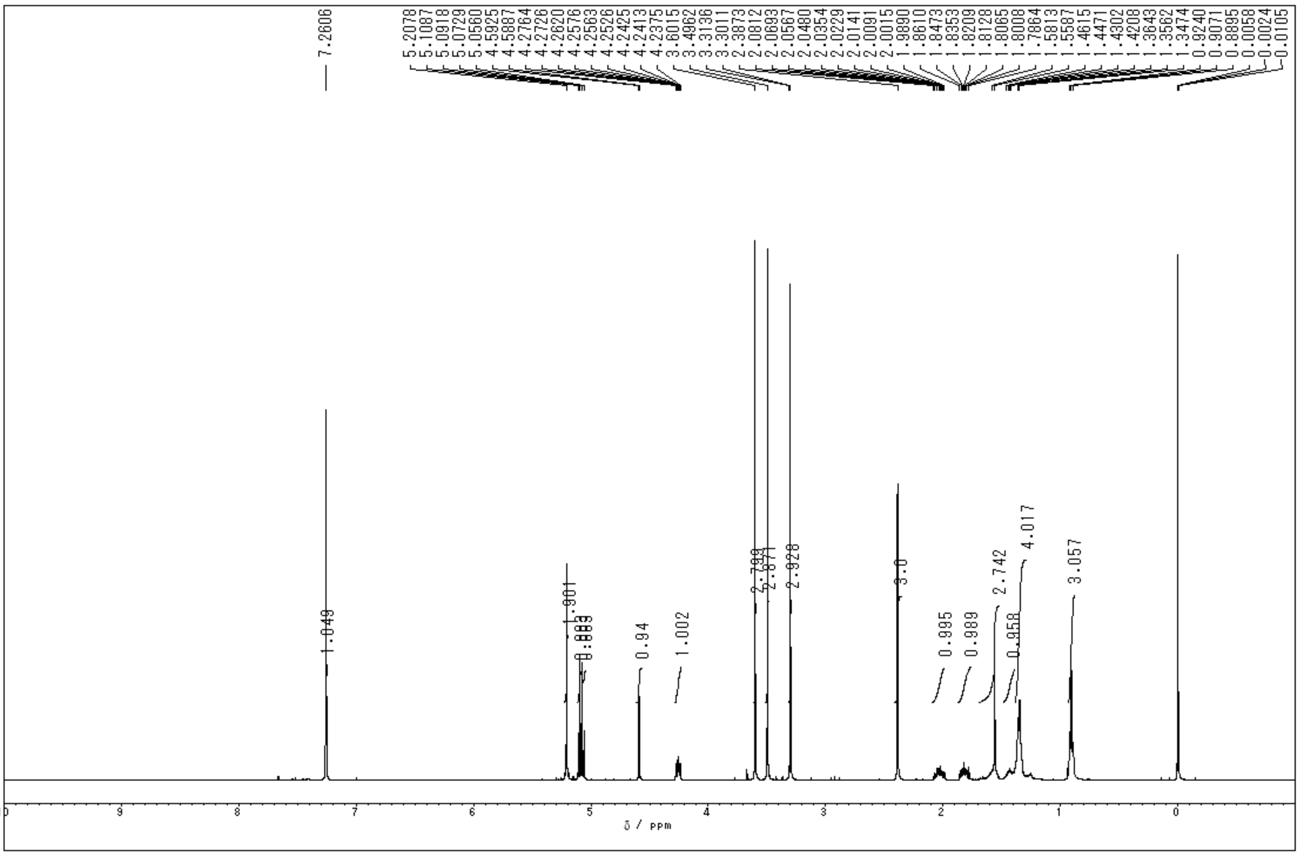

^{13}C-NMR ($CDCl_3$, 125 MHz)

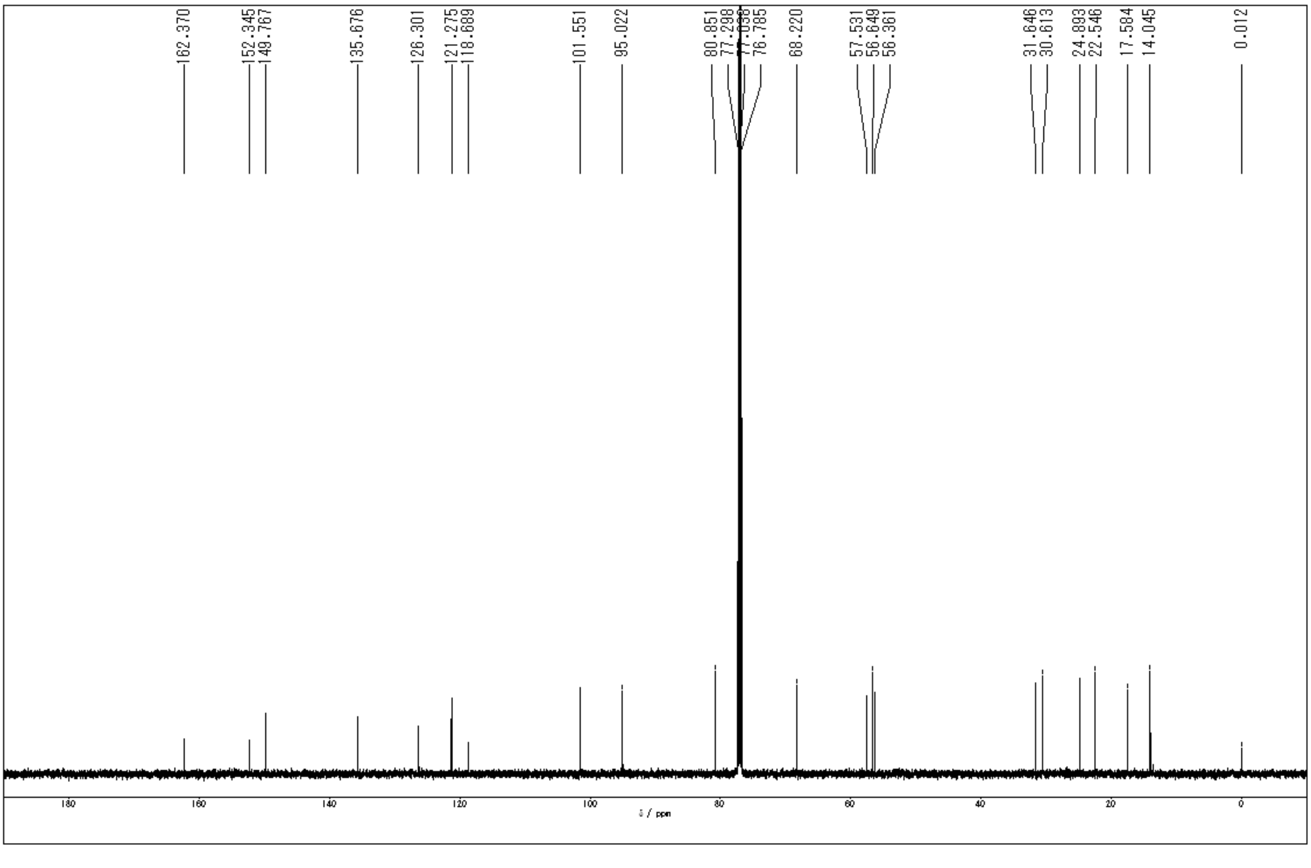

Compound 5

^{1}H-NMR ($CDCl_3$, 400 MHz)

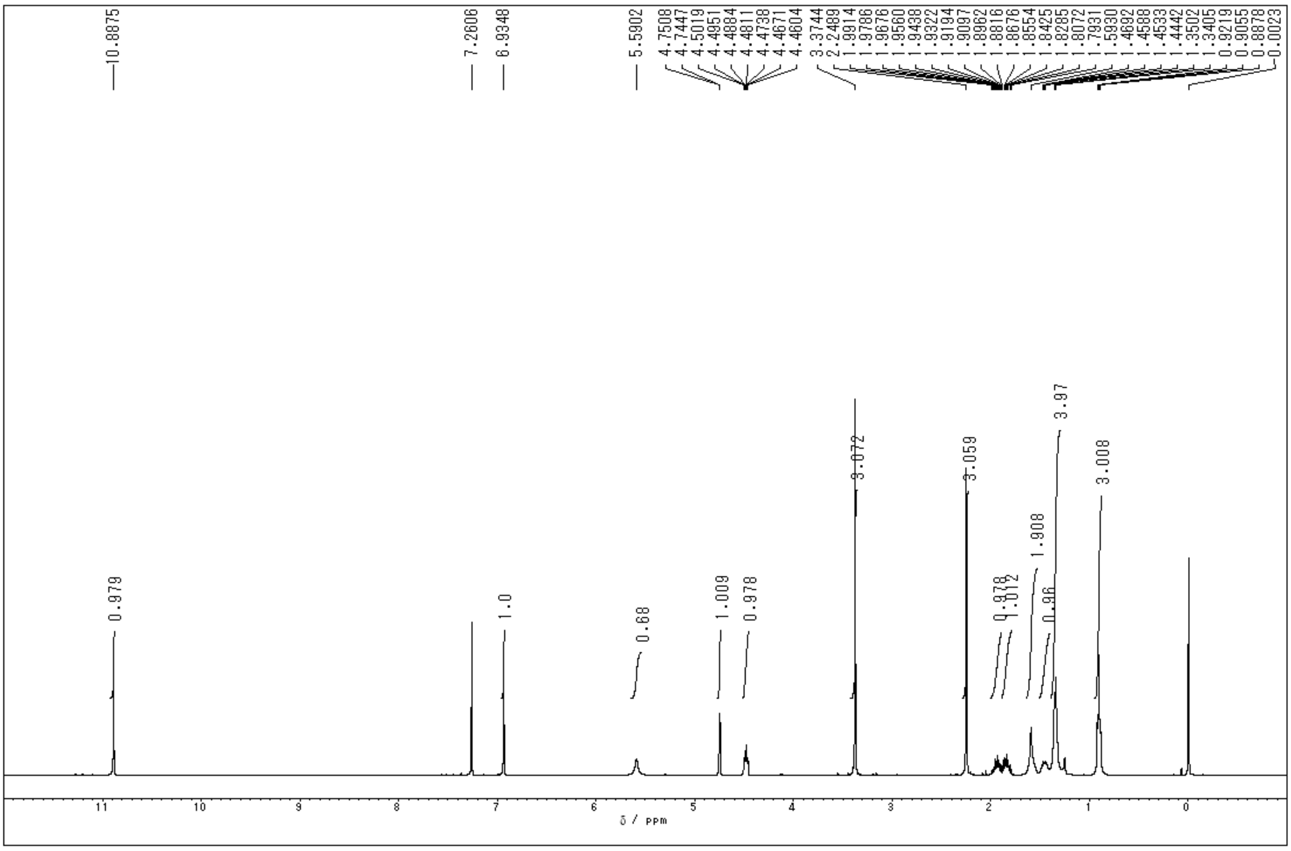

^{13}C-NMR ($CDCl_3$, 125 MHz)

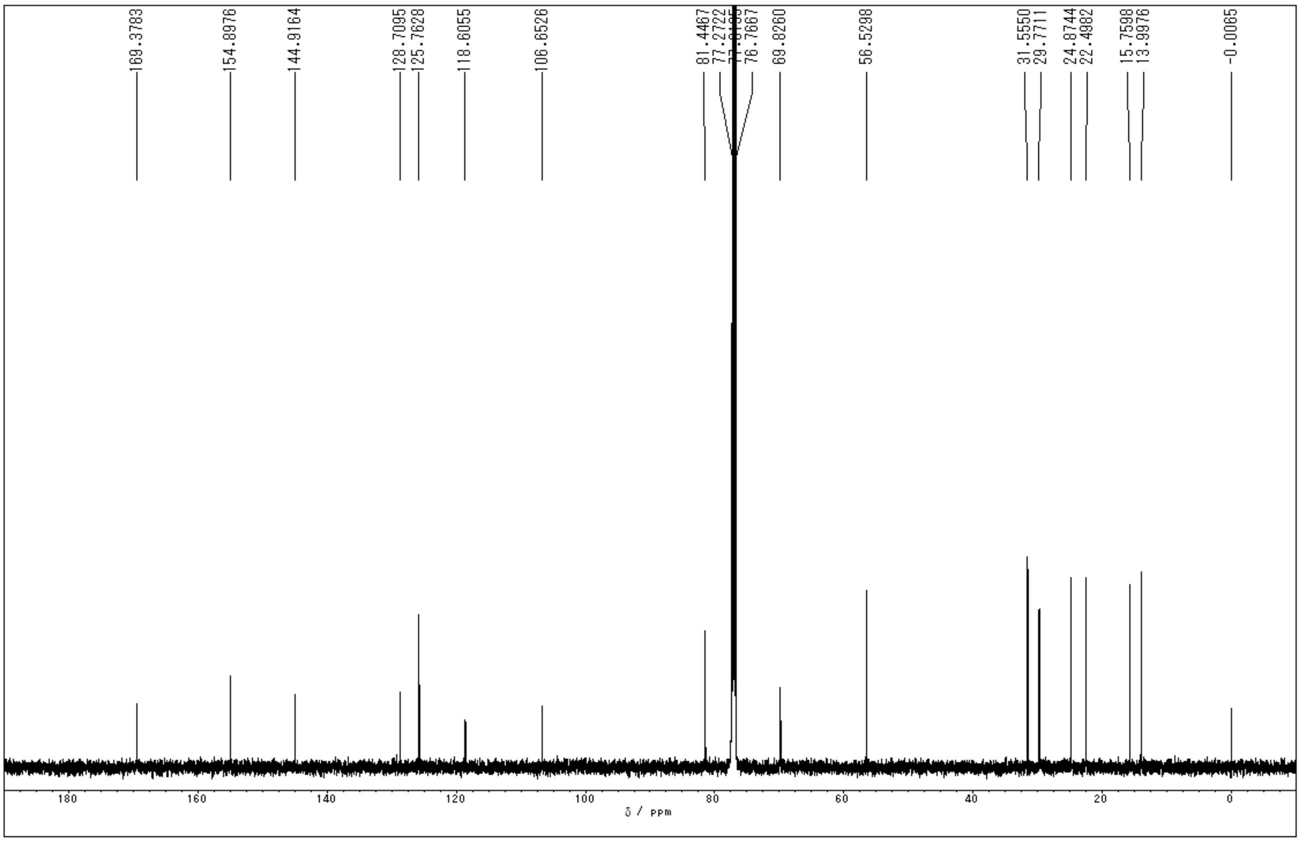

Compound 6

^{1}H-NMR ($CDCl_3$, 500 MHz)

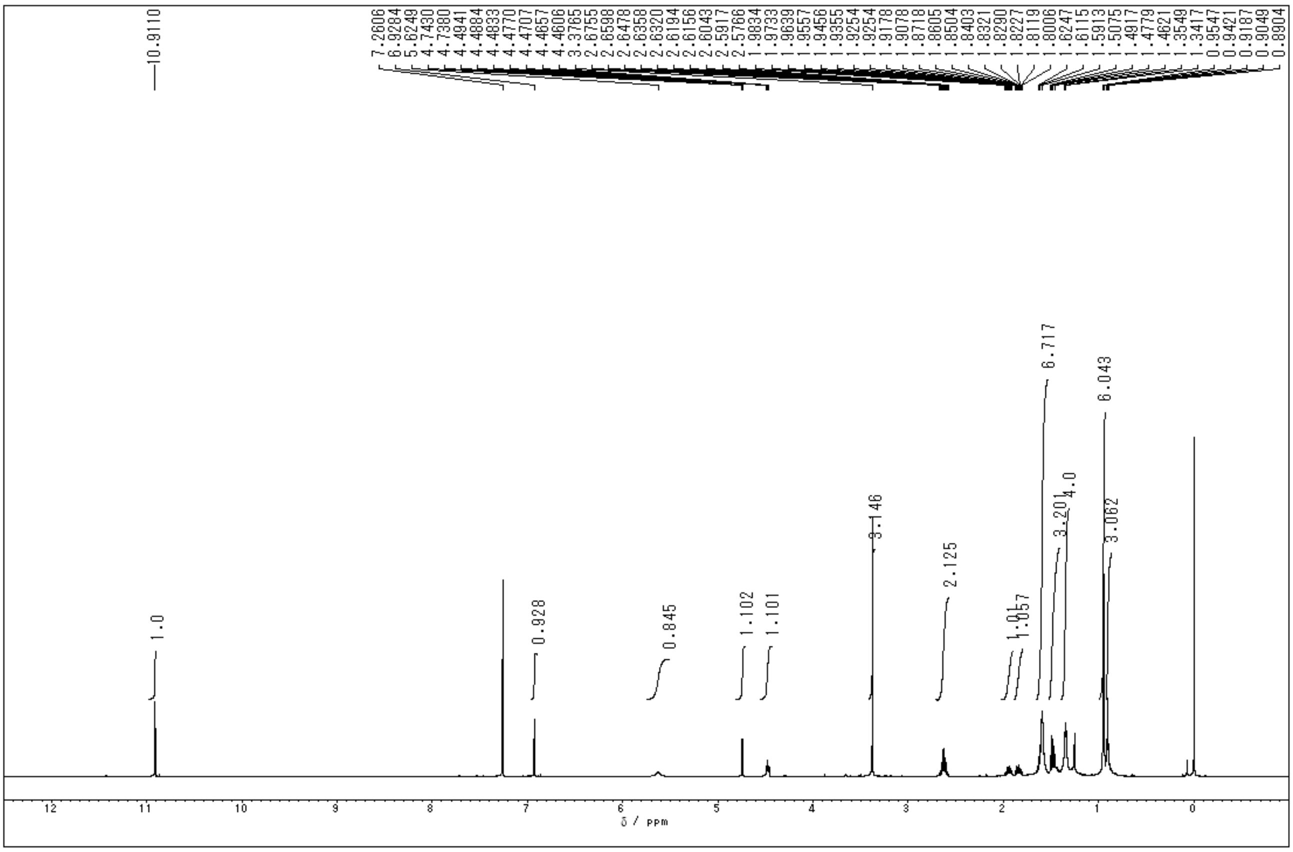

^{13}C-NMR ($CDCl_3$, 125 MHz)

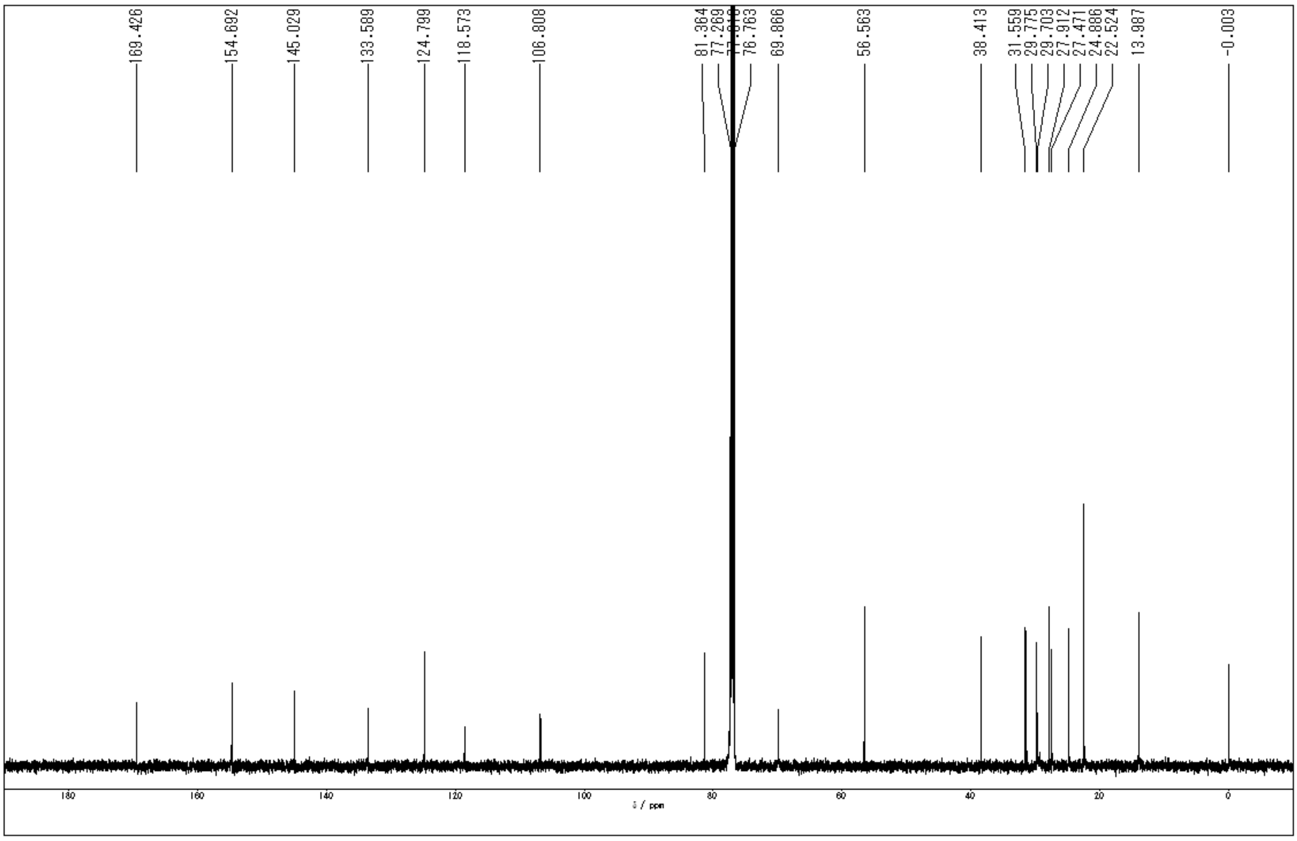

Compound 7a

^{1}H-NMR ($CDCl_3$, 500 MHz)

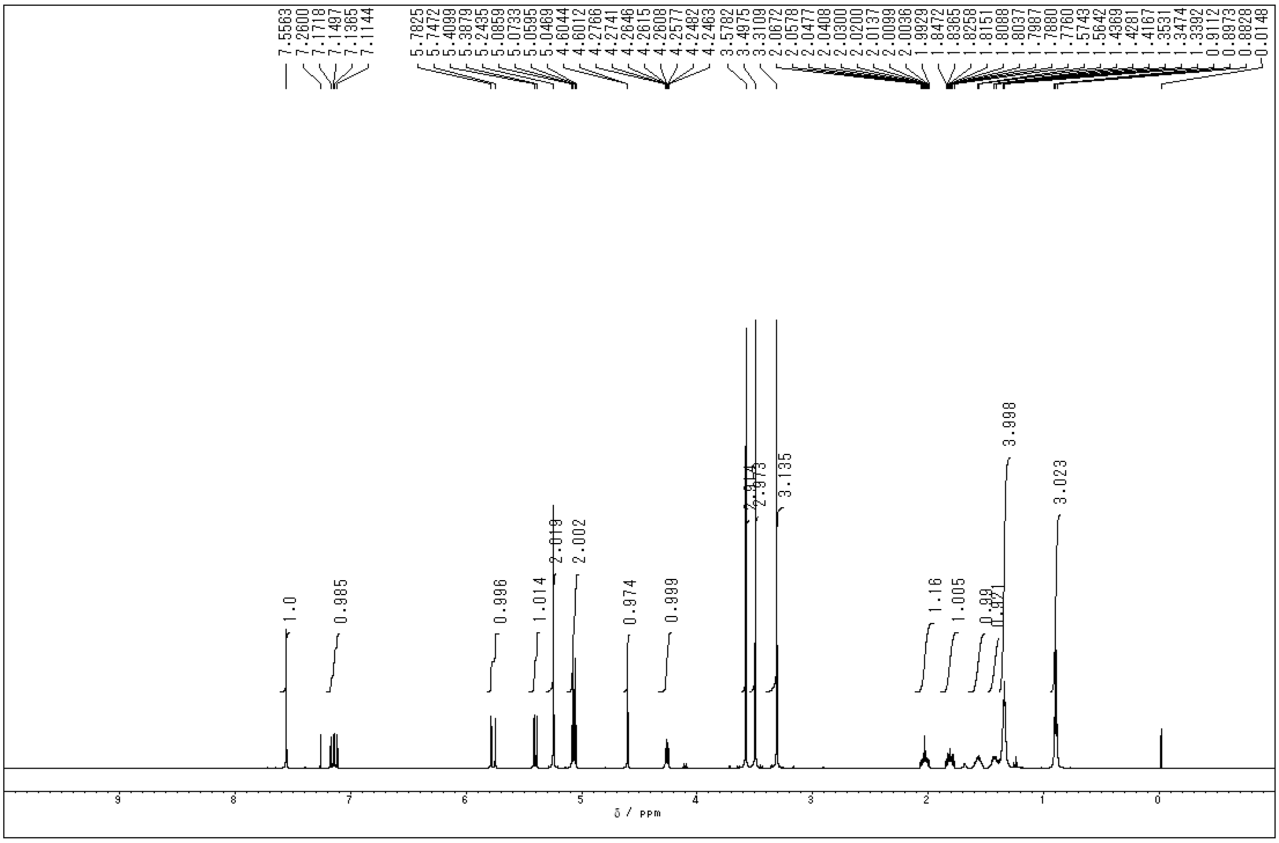

^{13}C-NMR ($CDCl_3$, 125 MHz)

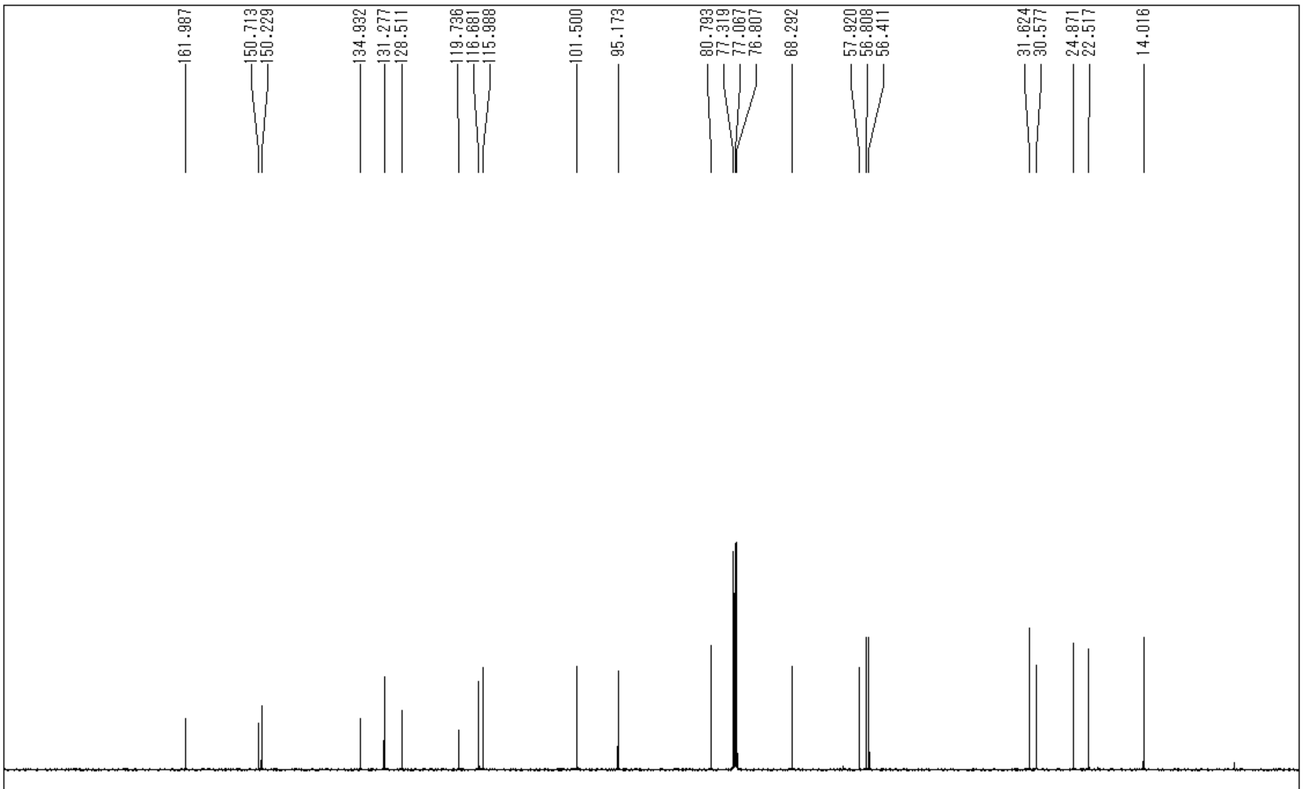

Compound 7

^{1}H-NMR ($CDCl_3$, 500 MHz)

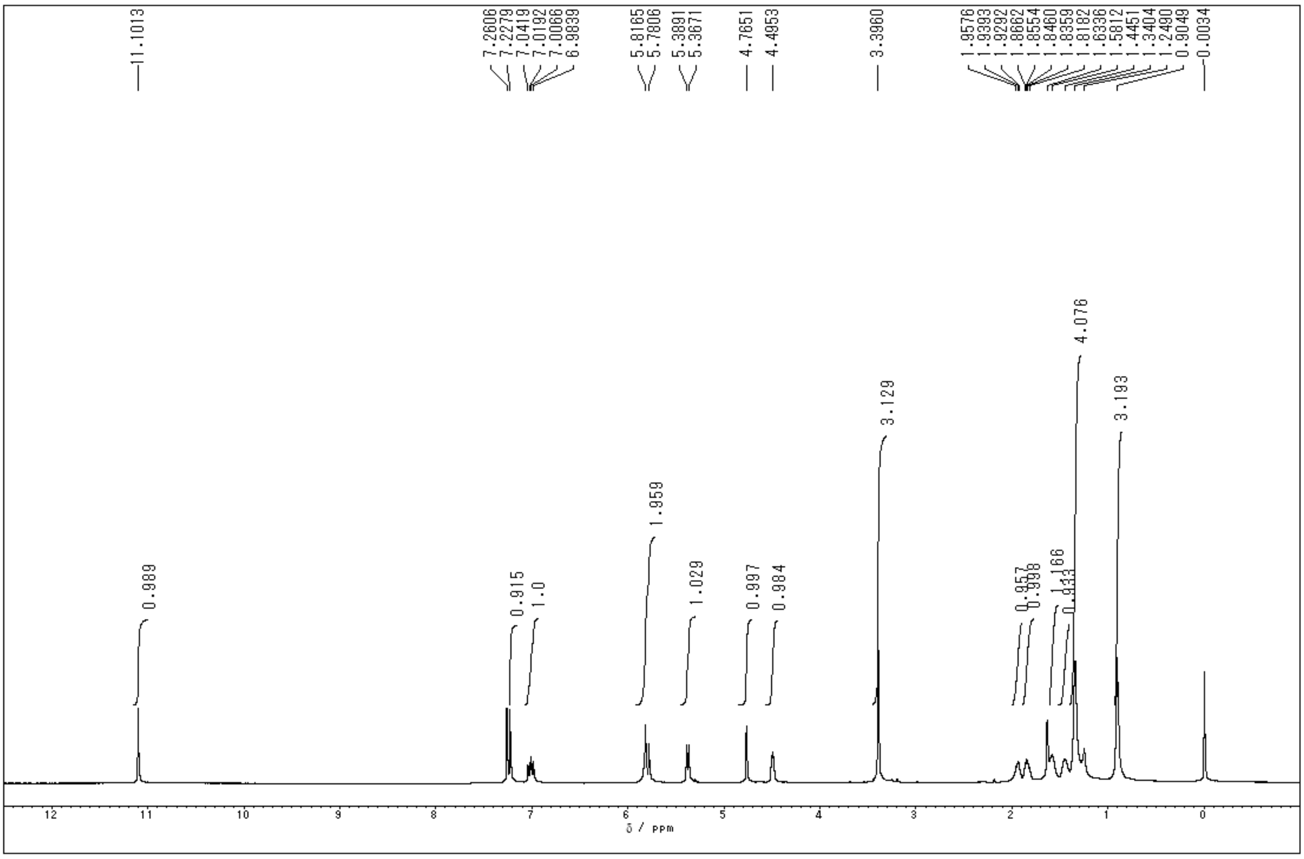

^{13}C-NMR ($CDCl_3$, 125 MHz)

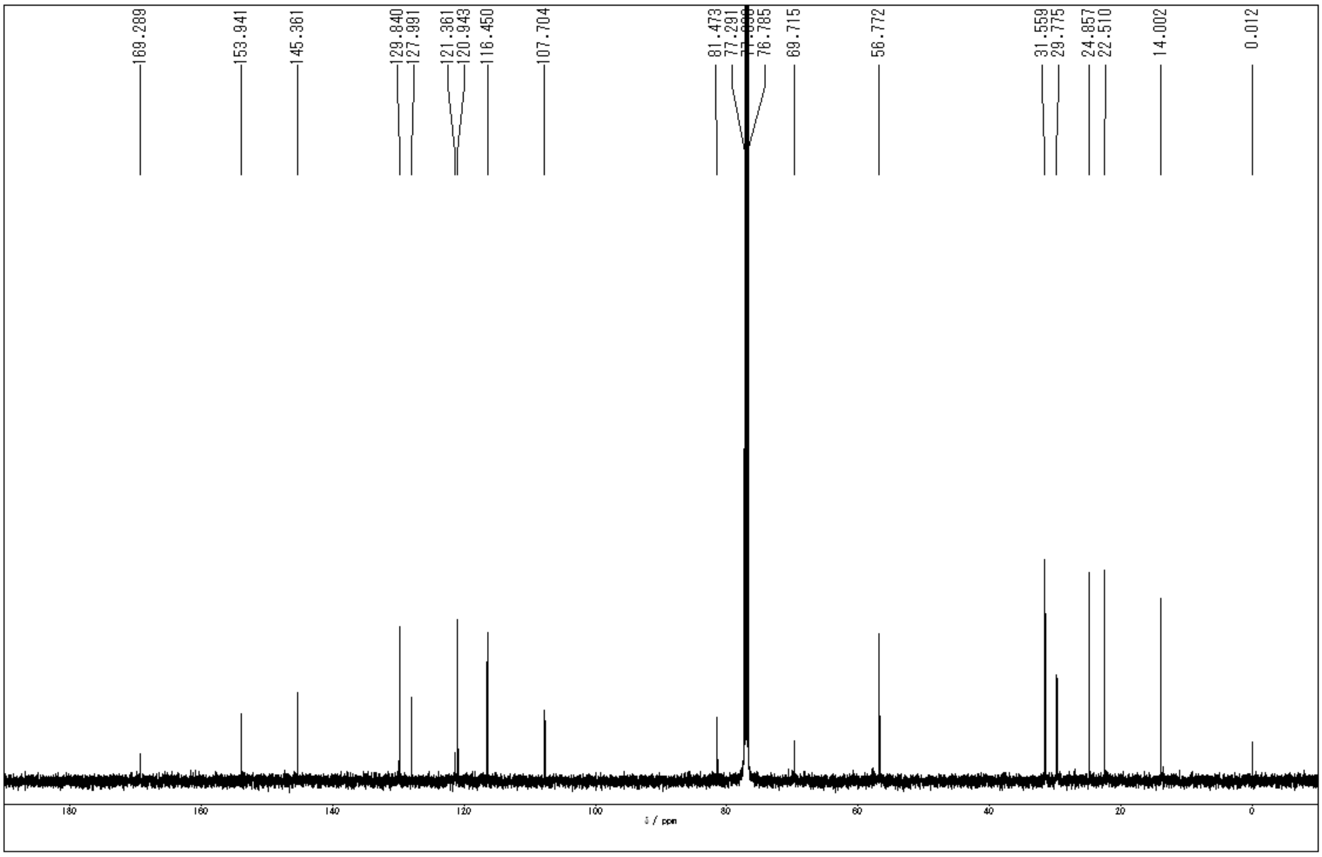

Compound 8a

^{1}H-NMR ($CDCl_3$, 500 MHz)

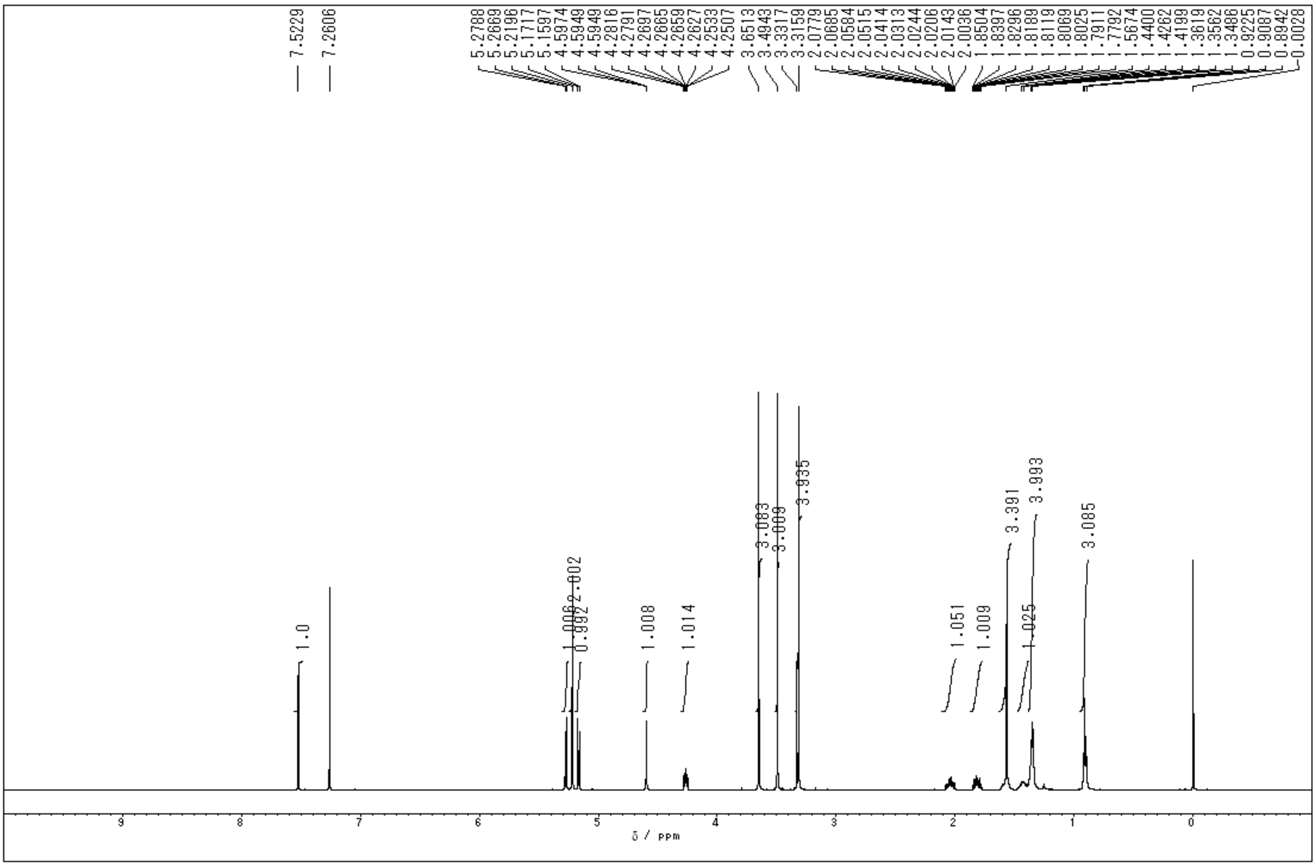

^{13}C-NMR ($CDCl_3$, 125 MHz)

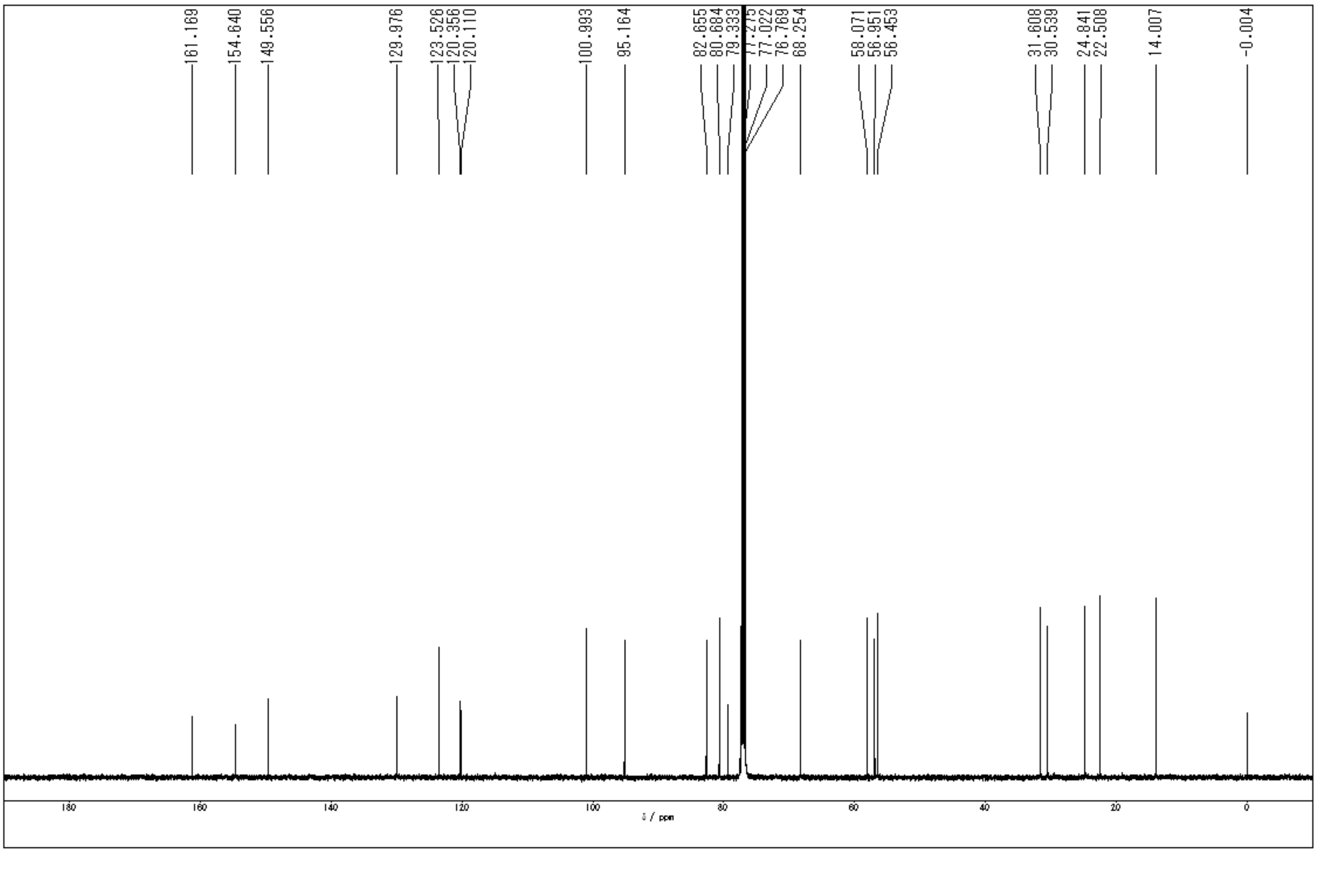

Compound 8

^{1}H-NMR ($CDCl_3$, 500 MHz)

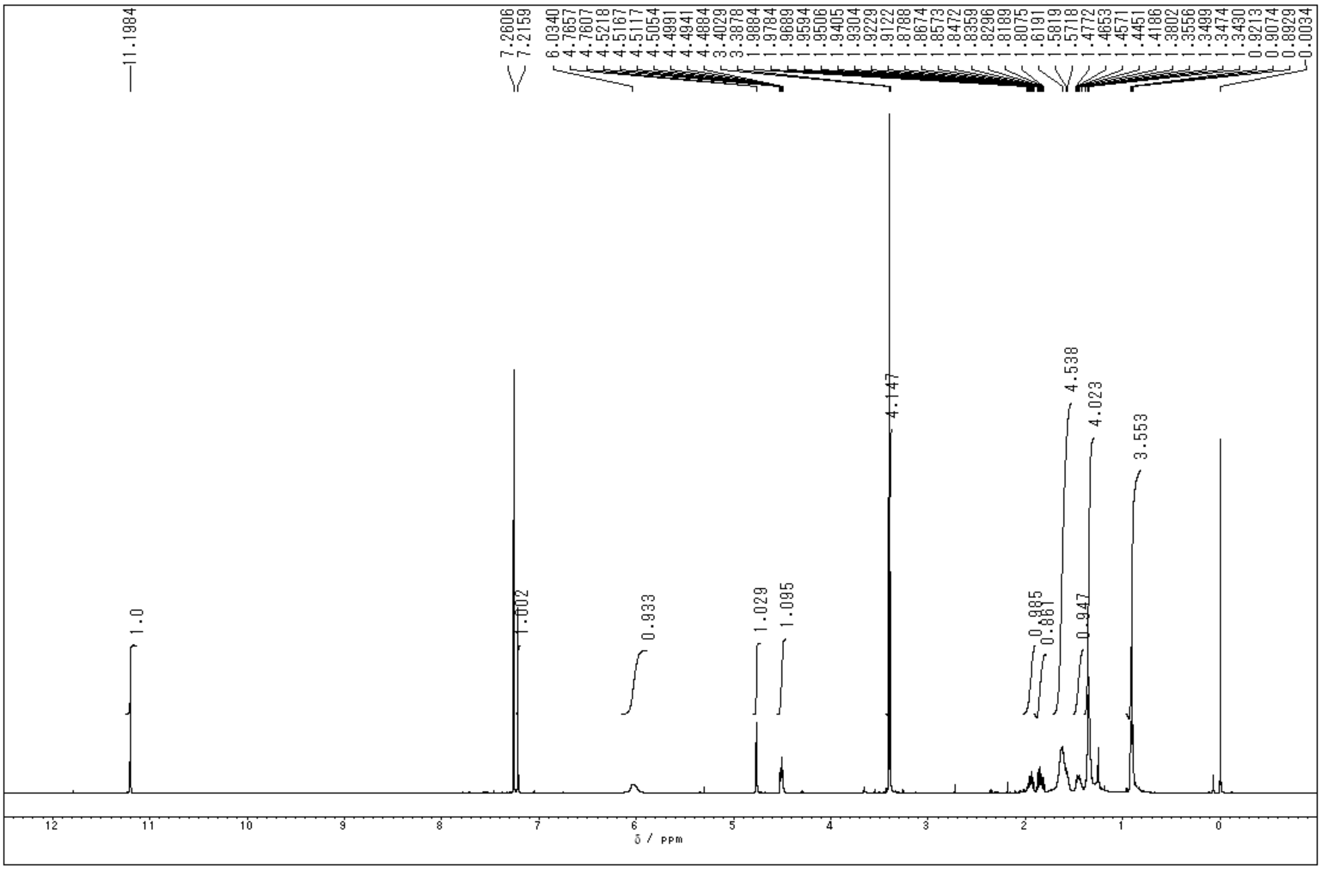

^{13}C-NMR ($CDCl_3$, 125 MHz)

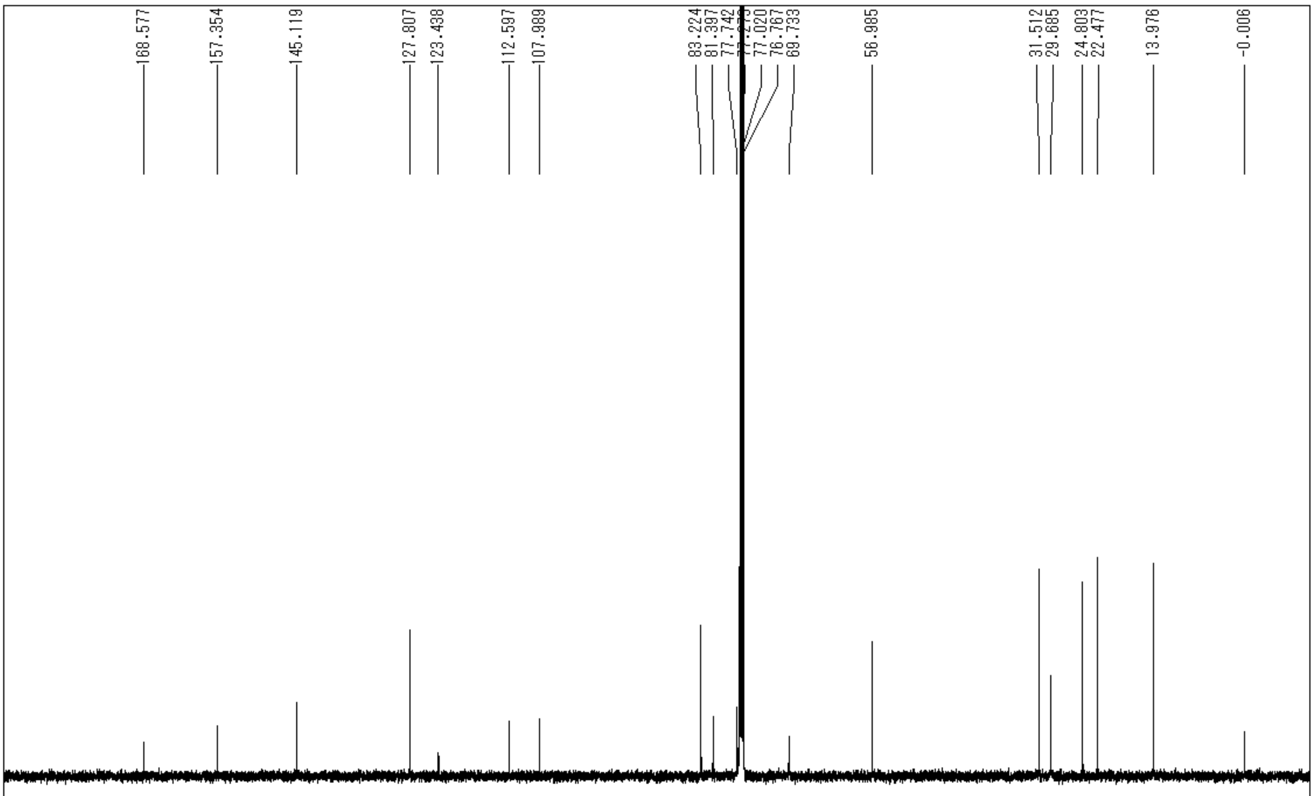

Compound 9a

^{1}H-NMR ($CDCl_3$, 500 MHz)

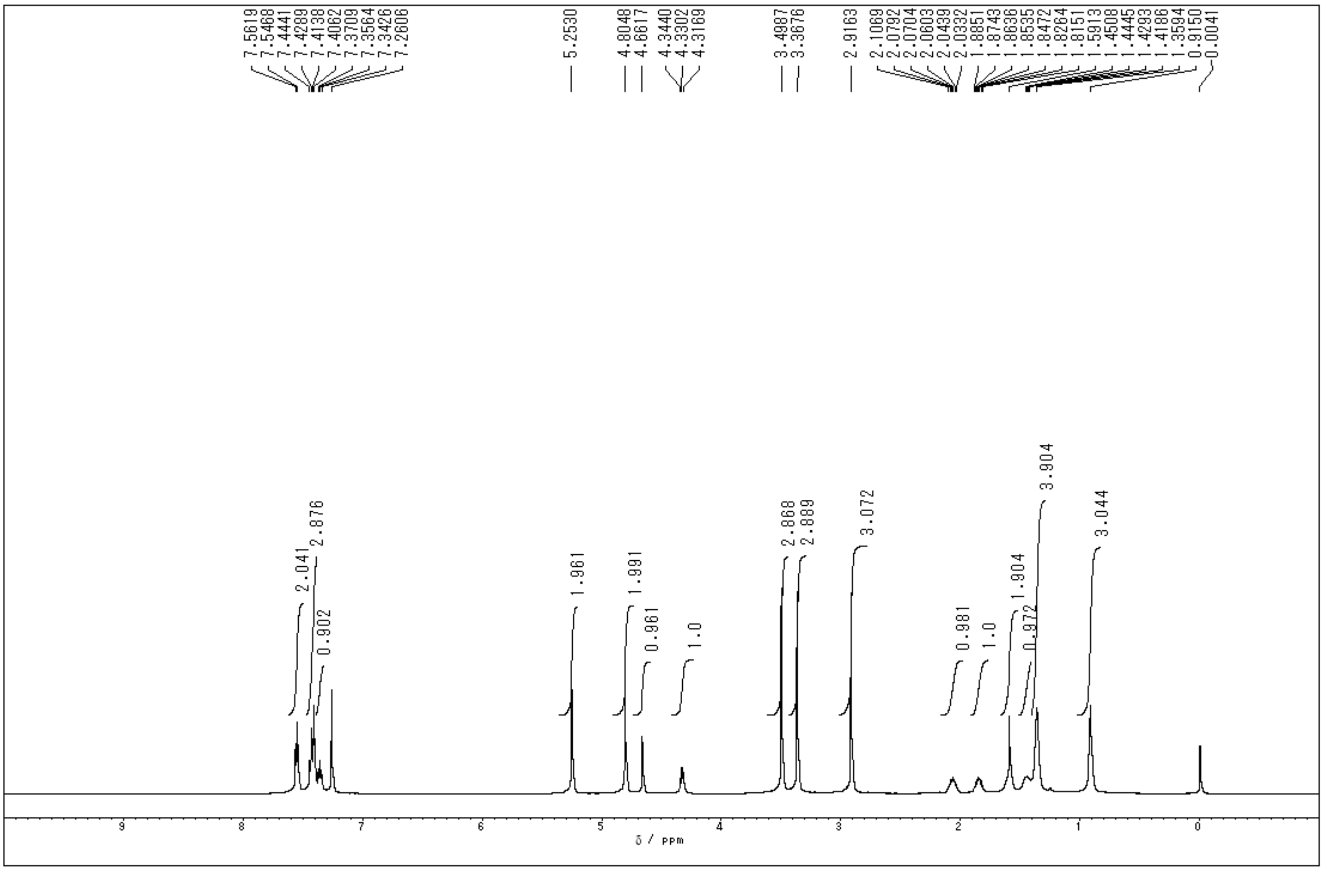

^{13}C-NMR ($CDCl_3$, 125 MHz)

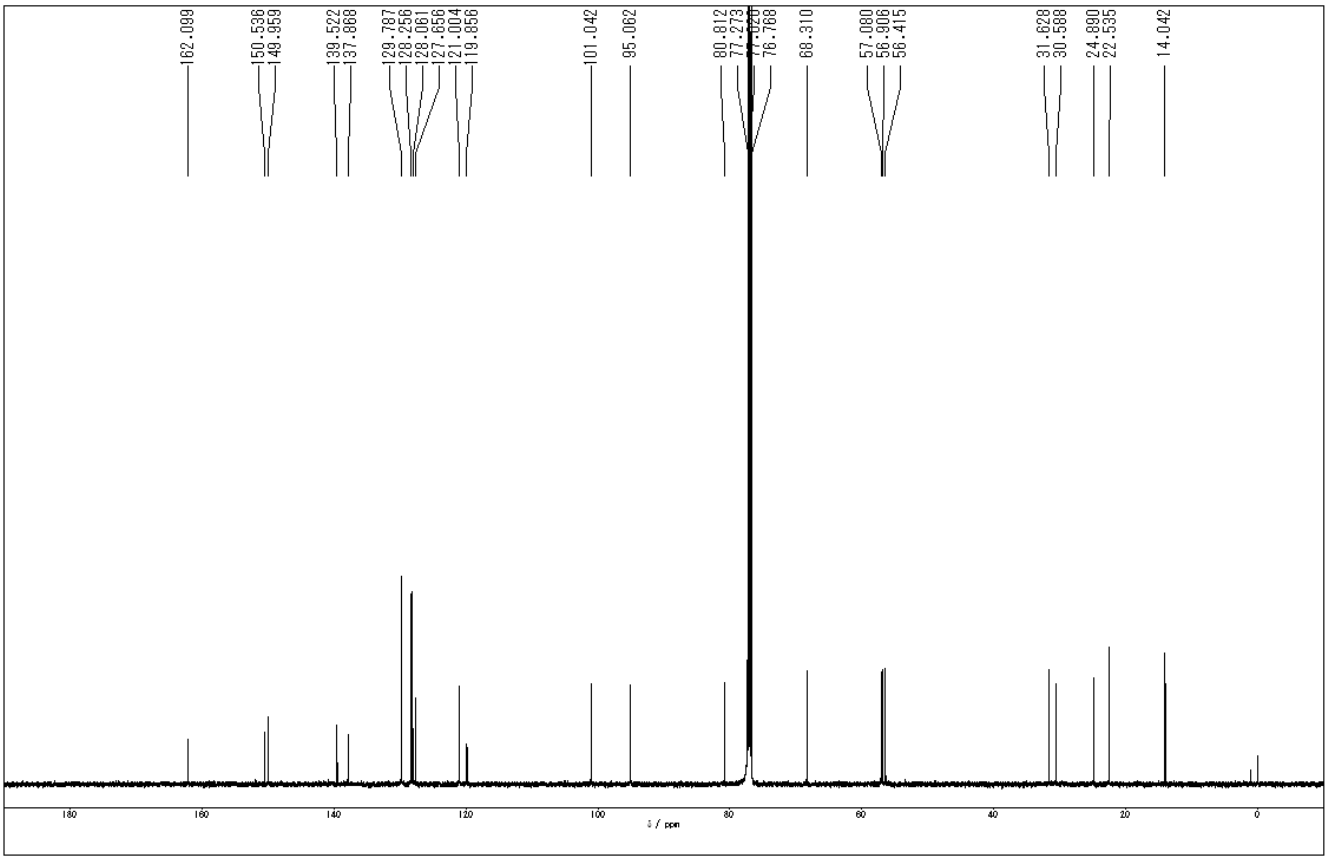

Compound 9

^{1}H-NMR ($CDCl_3$, 400 MHz)

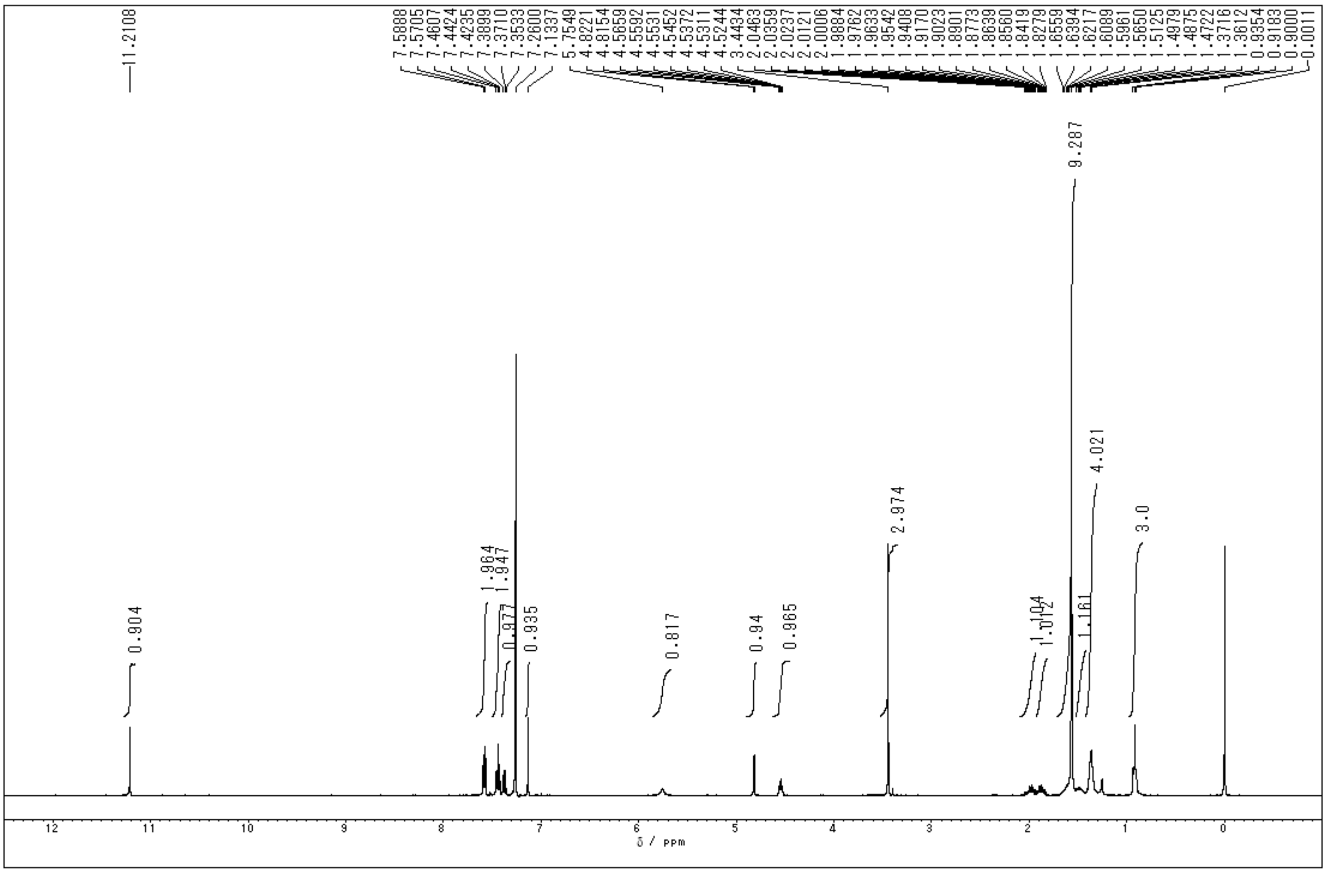

^{13}C-NMR ($CDCl_3$, 125 MHz)

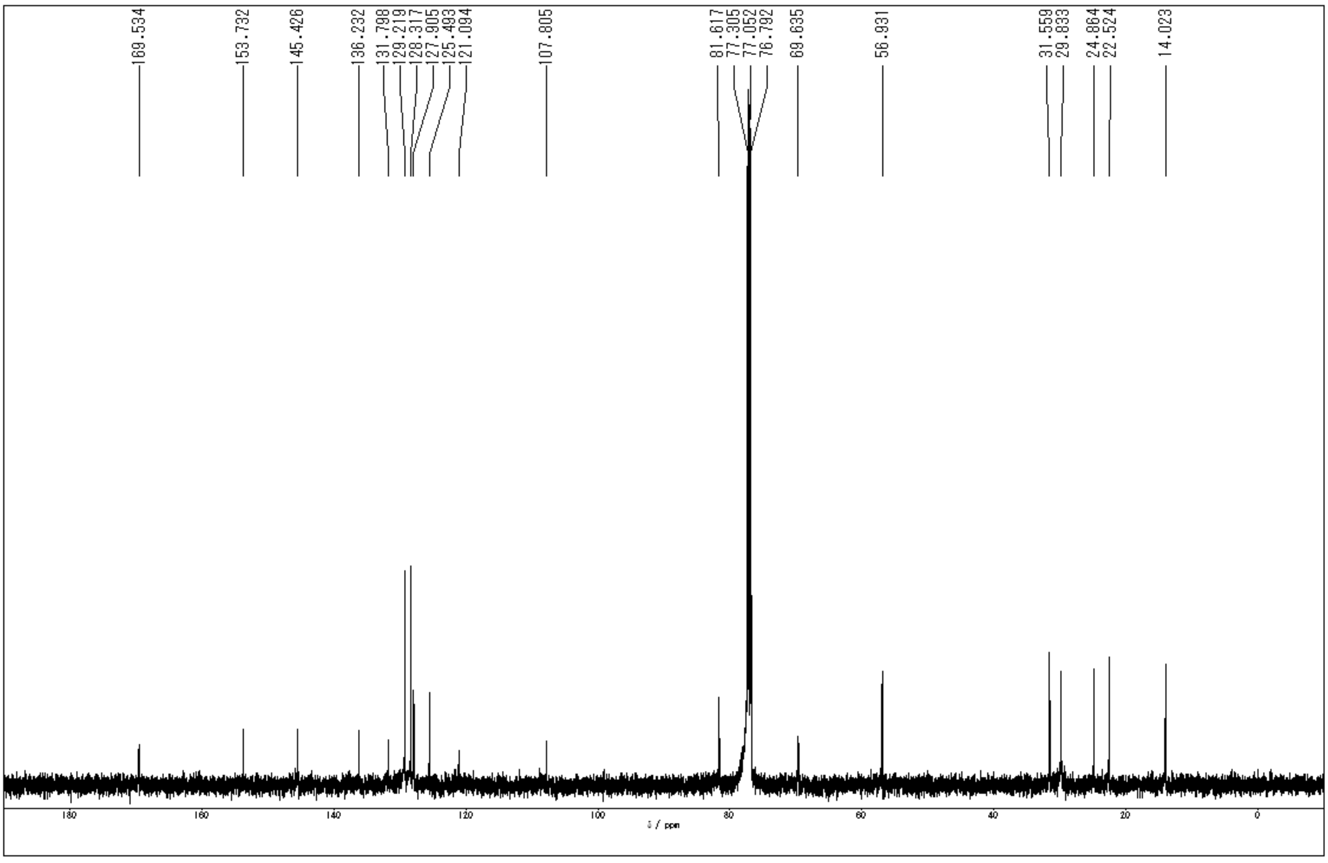

Compound 10a

^{1}H-NMR ($CDCl_3$, 500 MHz)

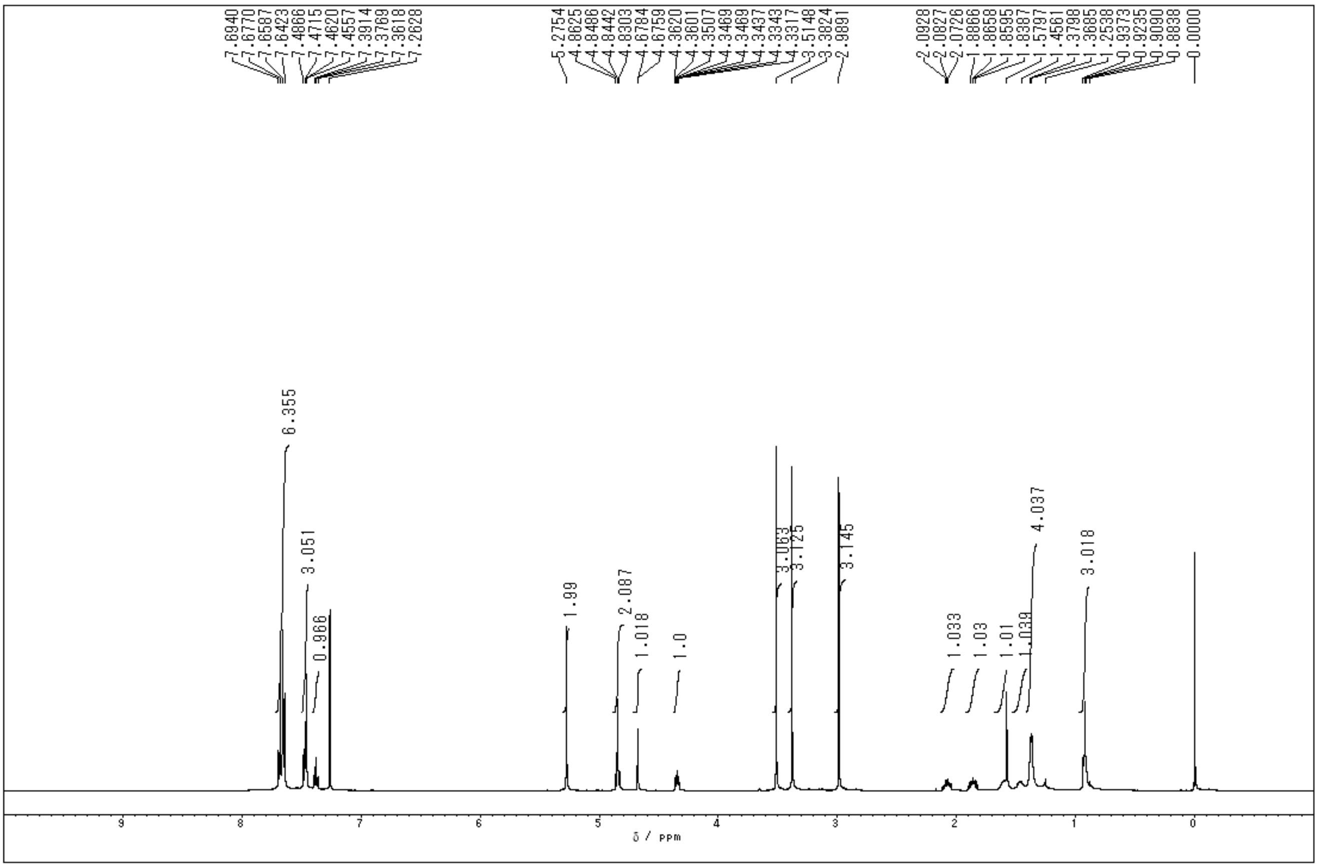

^{13}C-NMR ($CDCl_3$, 125 MHz)

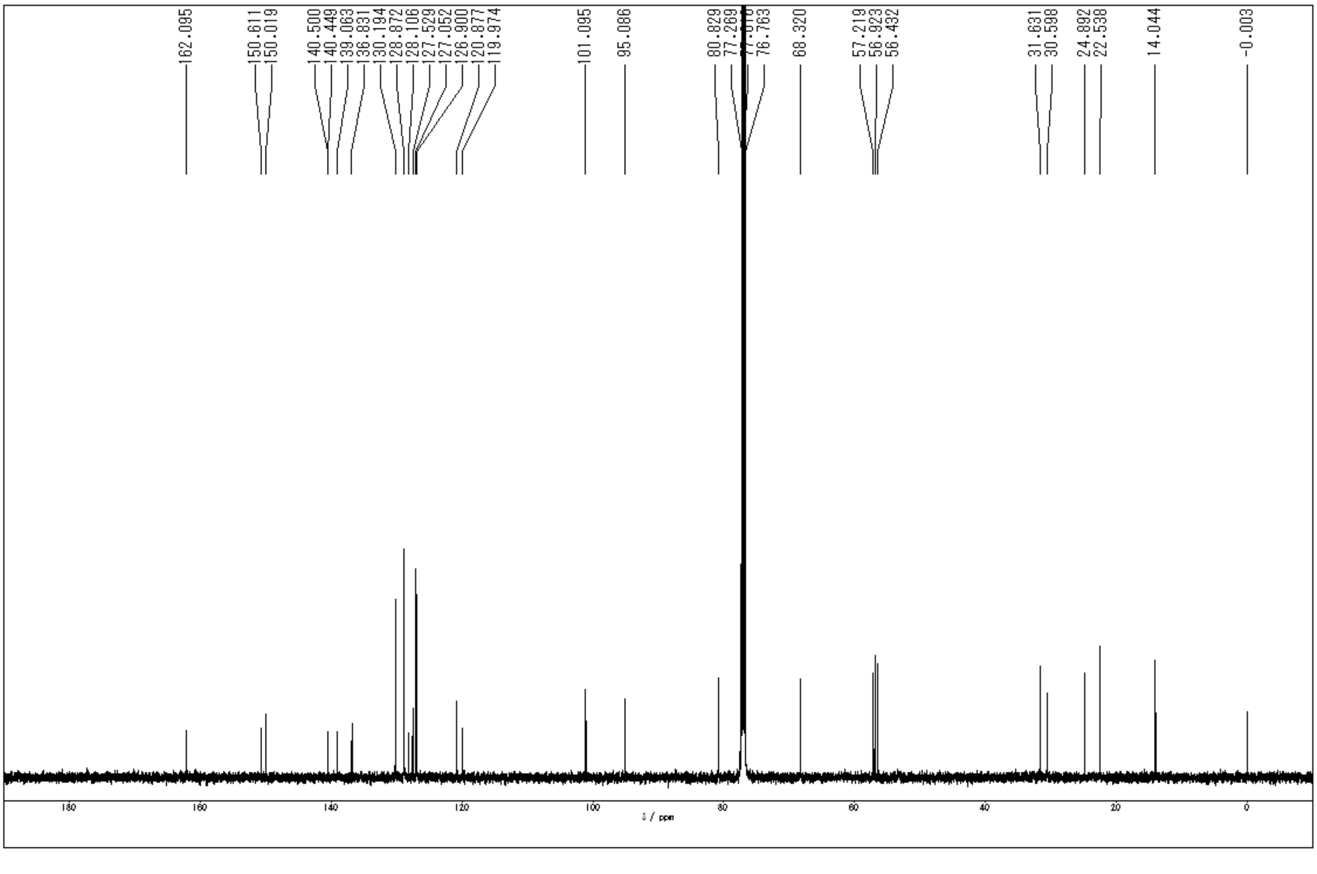

Compound 10

^{1}H-NMR ($CDCl_3$, 400 MHz)

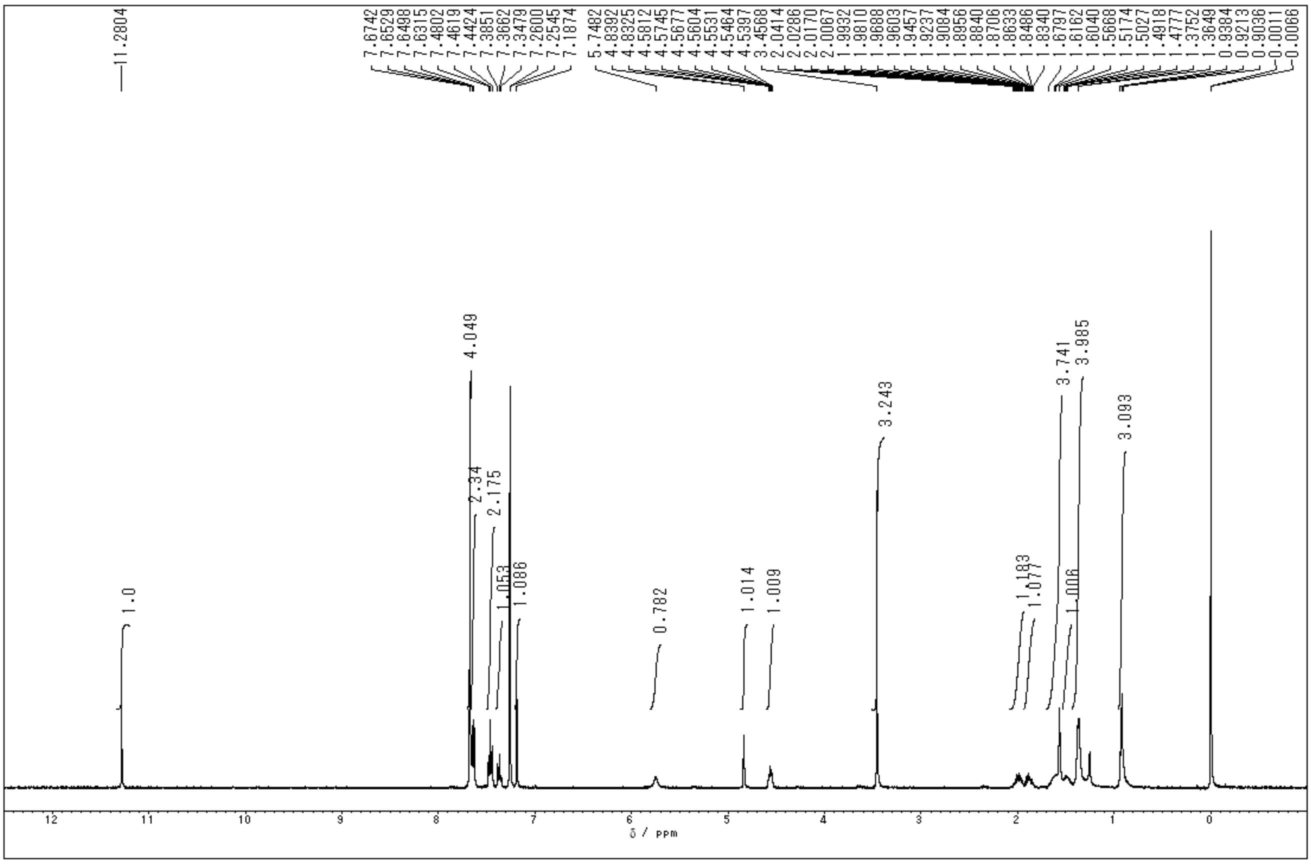

^{13}C-NMR ($CDCl_3$, 125 MHz)

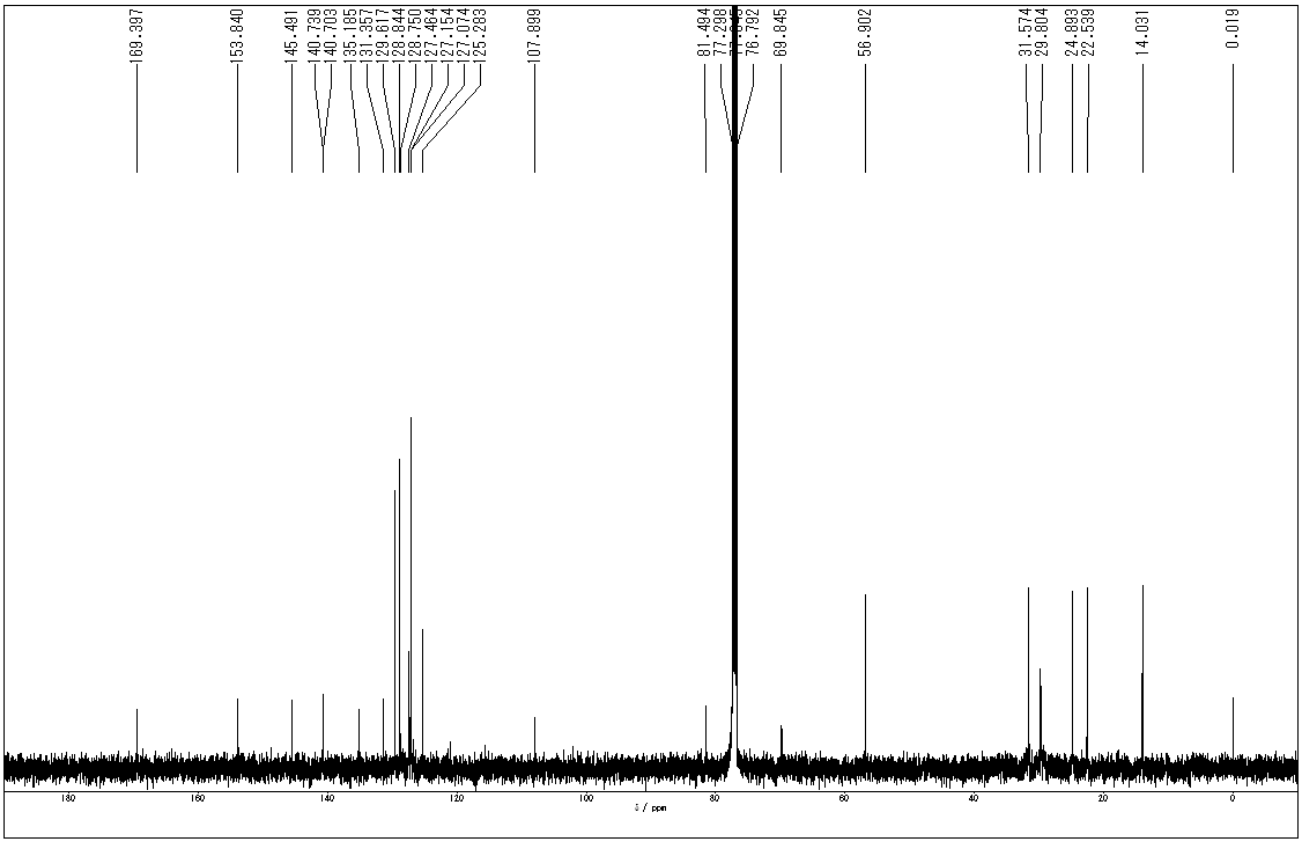

Compound 11a

^{1}H-NMR ($CDCl_3$, 400 MHz)

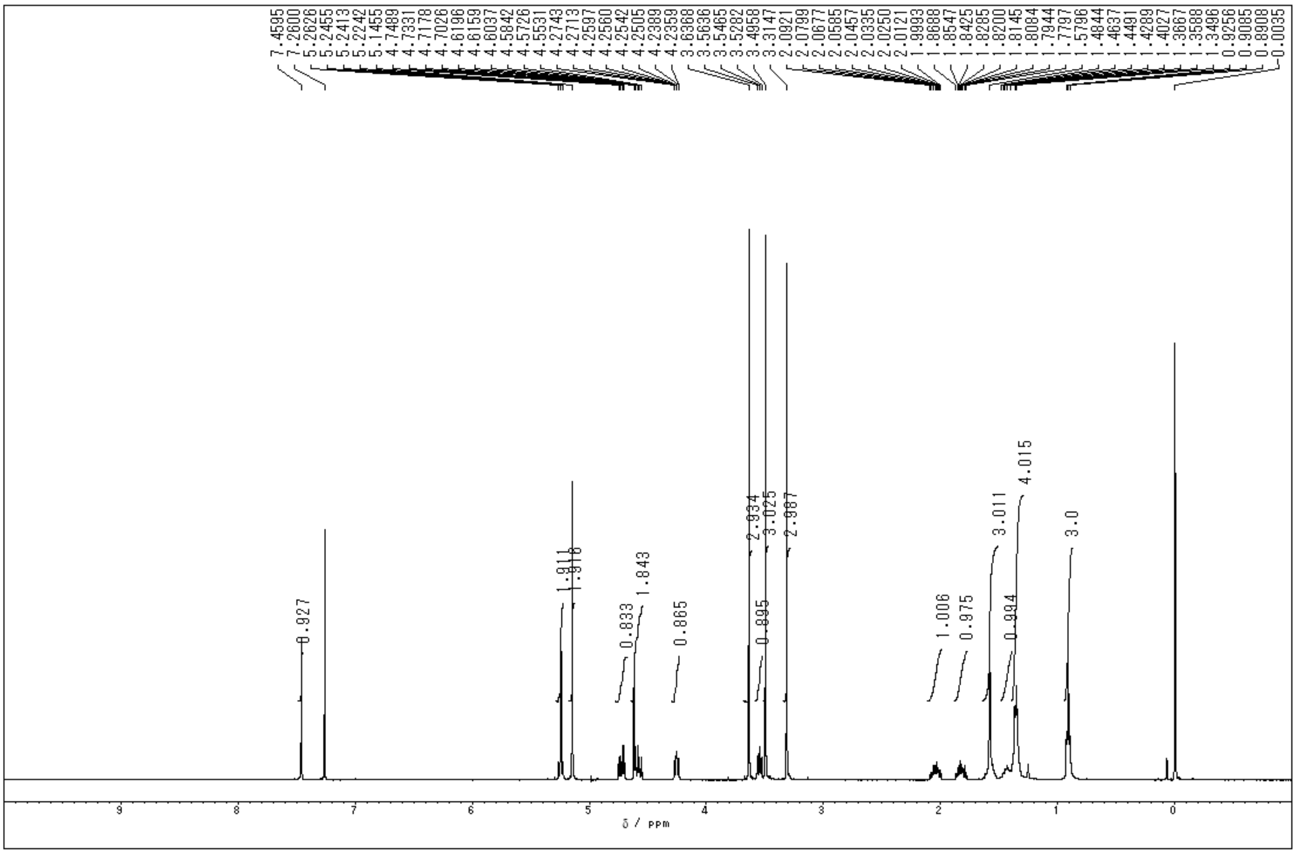

^{13}C-NMR ($CDCl_3$, 125 MHz)

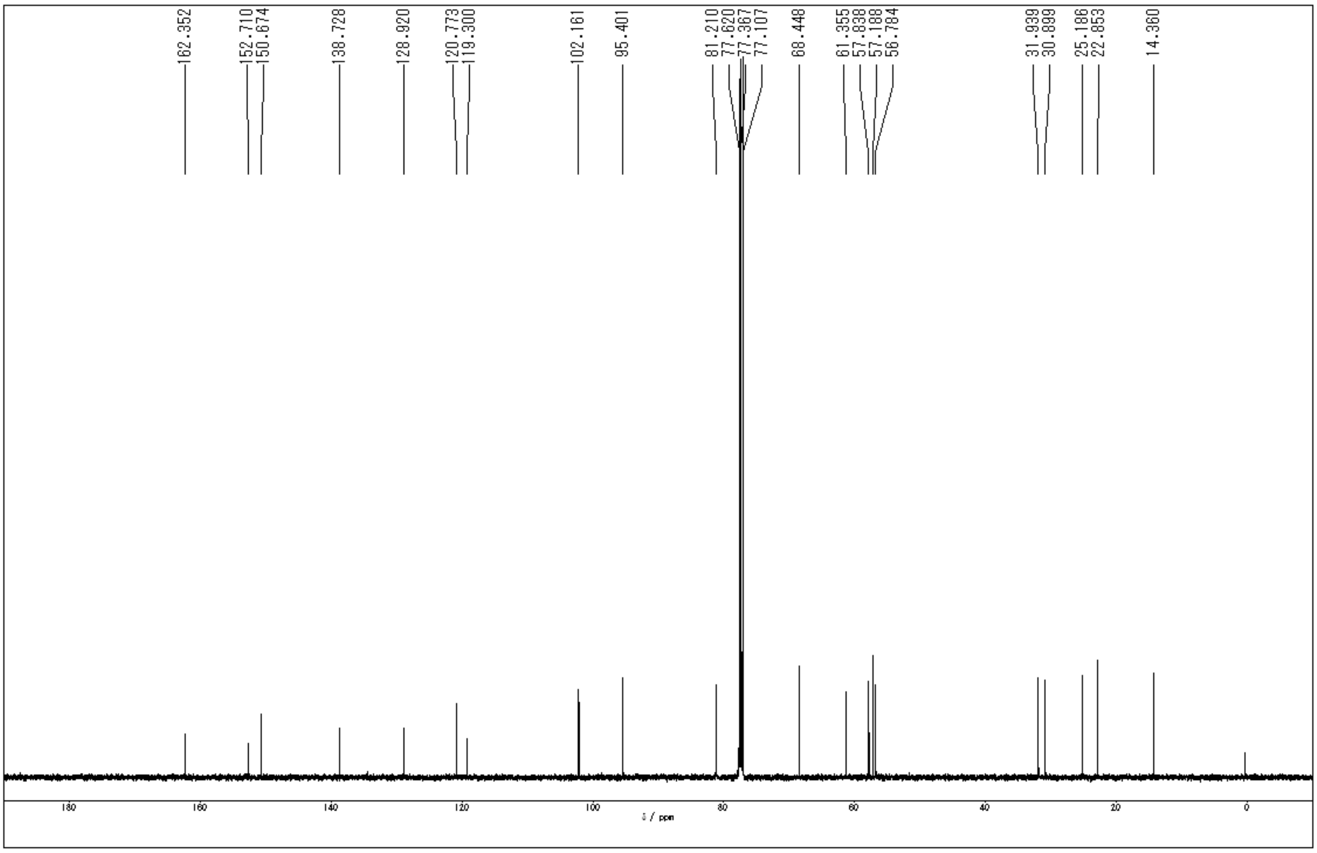

Compound 11

^{1}H-NMR ($CDCl_3$, 400 MHz)

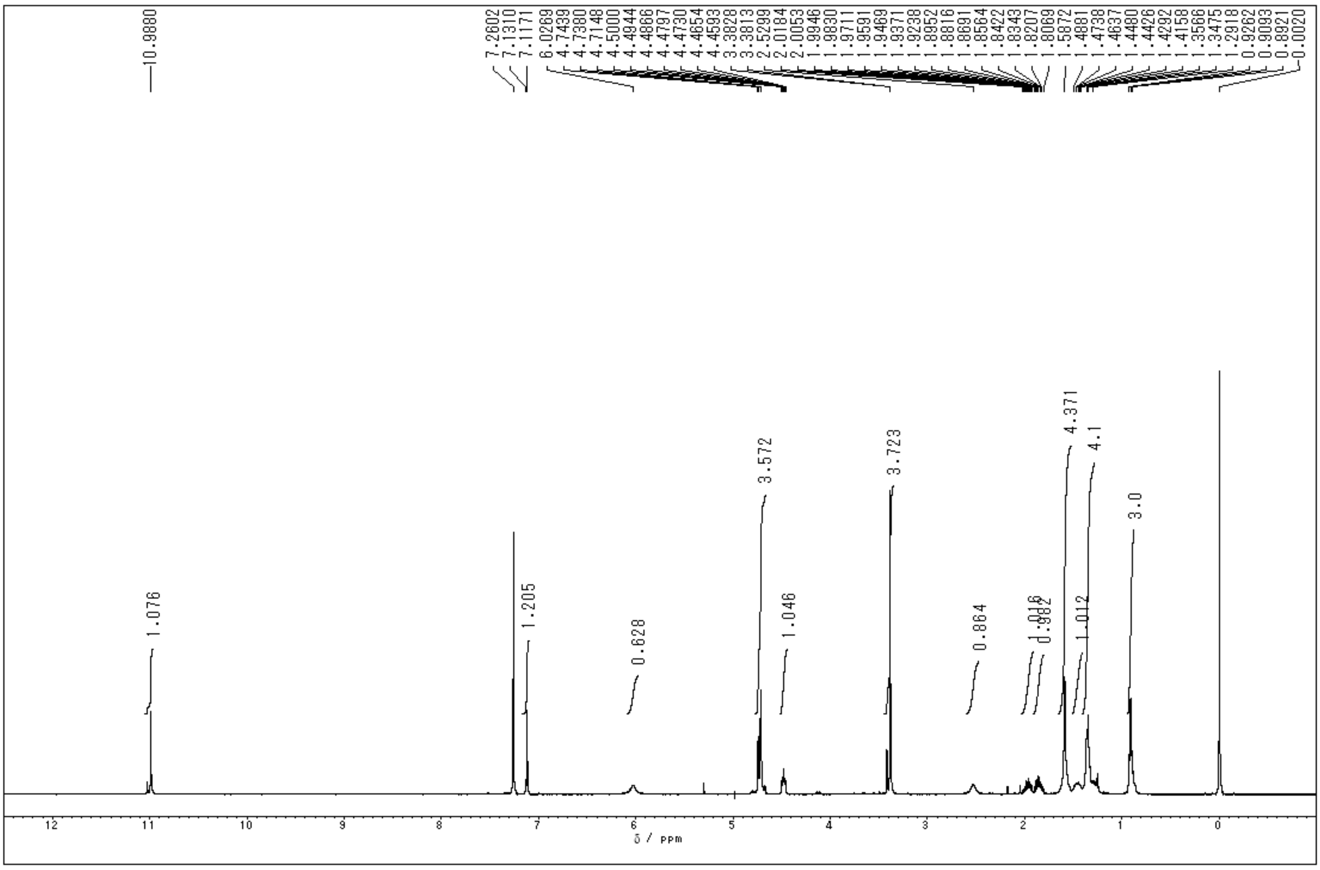

^{13}C-NMR ($CDCl_3$, 125 MHz)

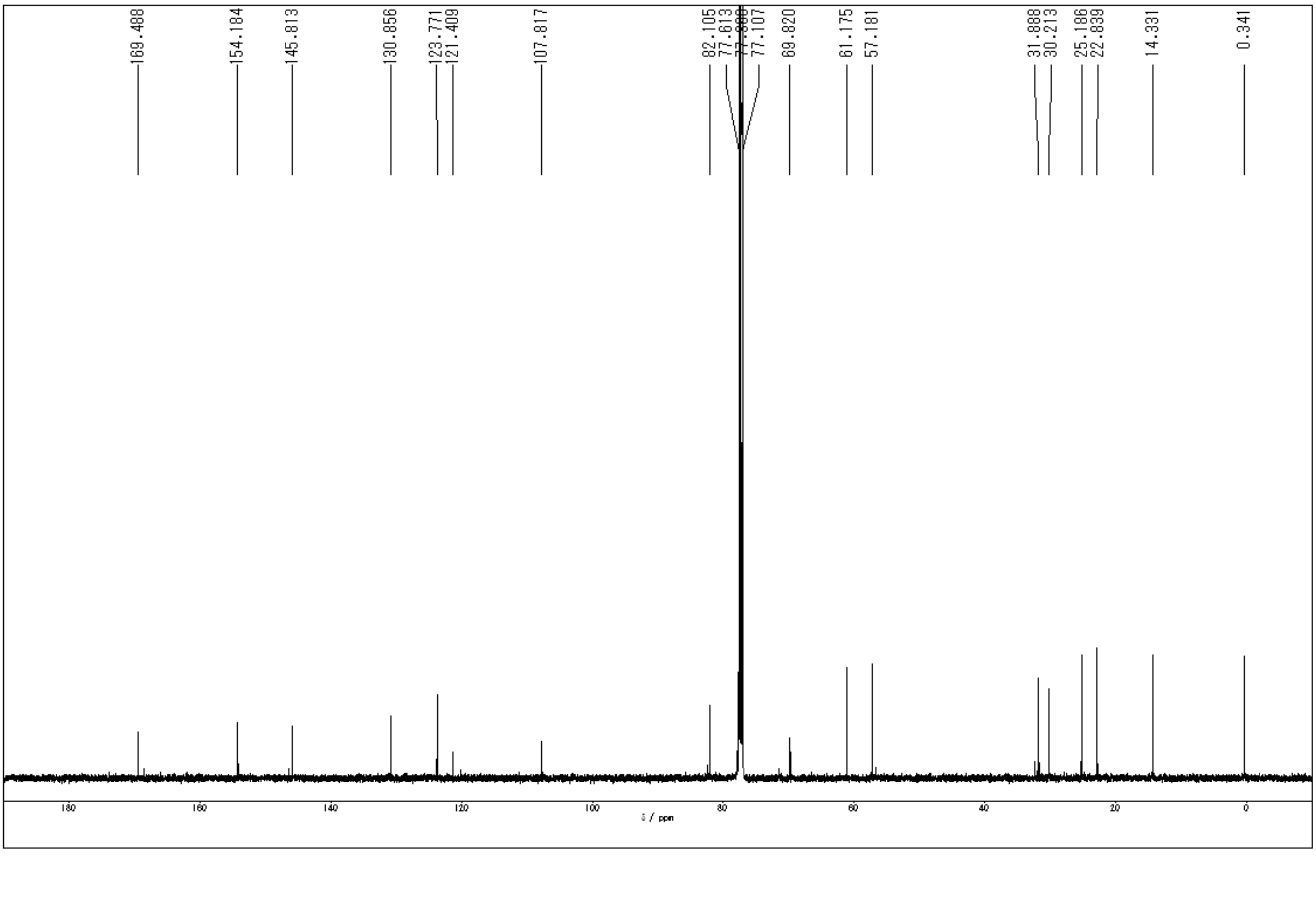

Compound 12a

^{1}H-NMR ($CDCl_3$, 400 MHz)

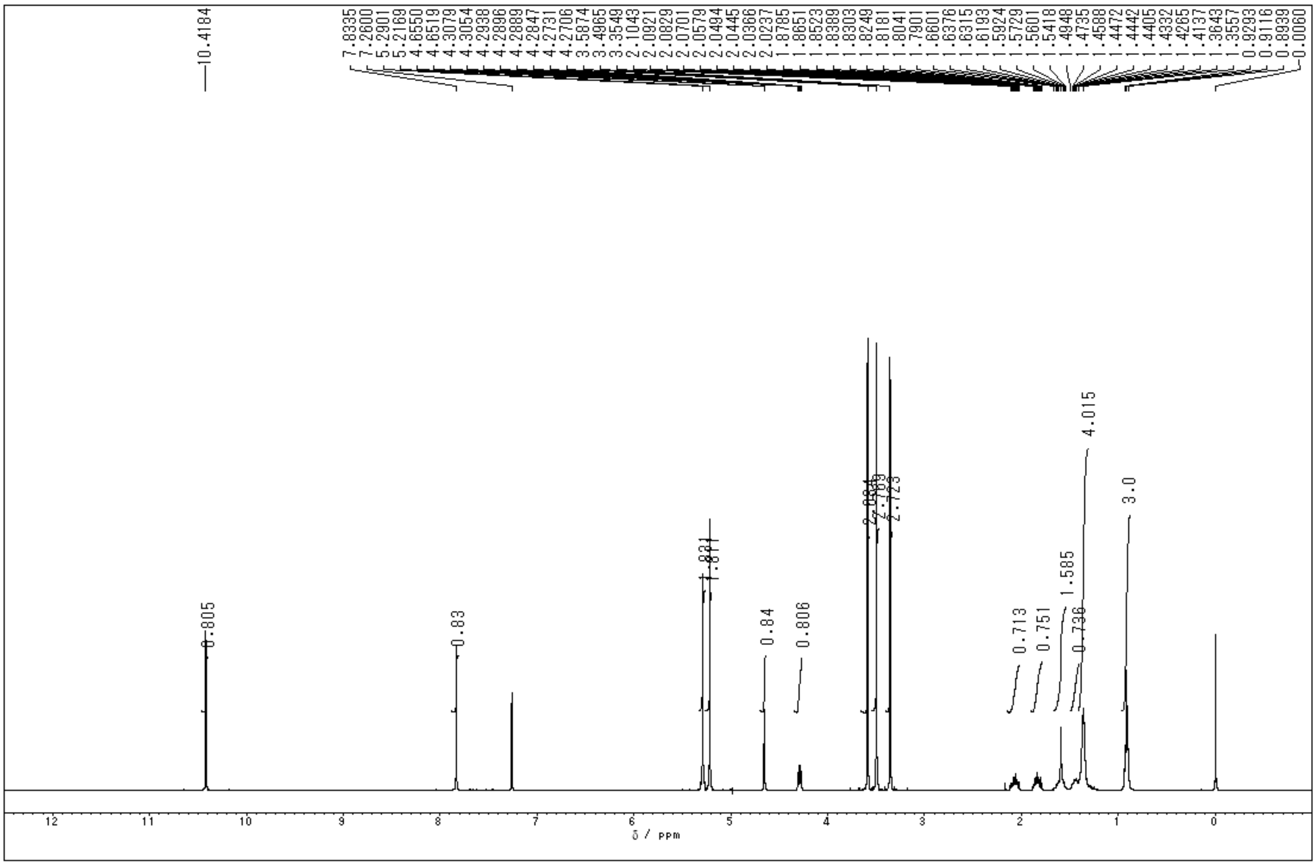

^{13}C-NMR ($CDCl_3$, 125 MHz)

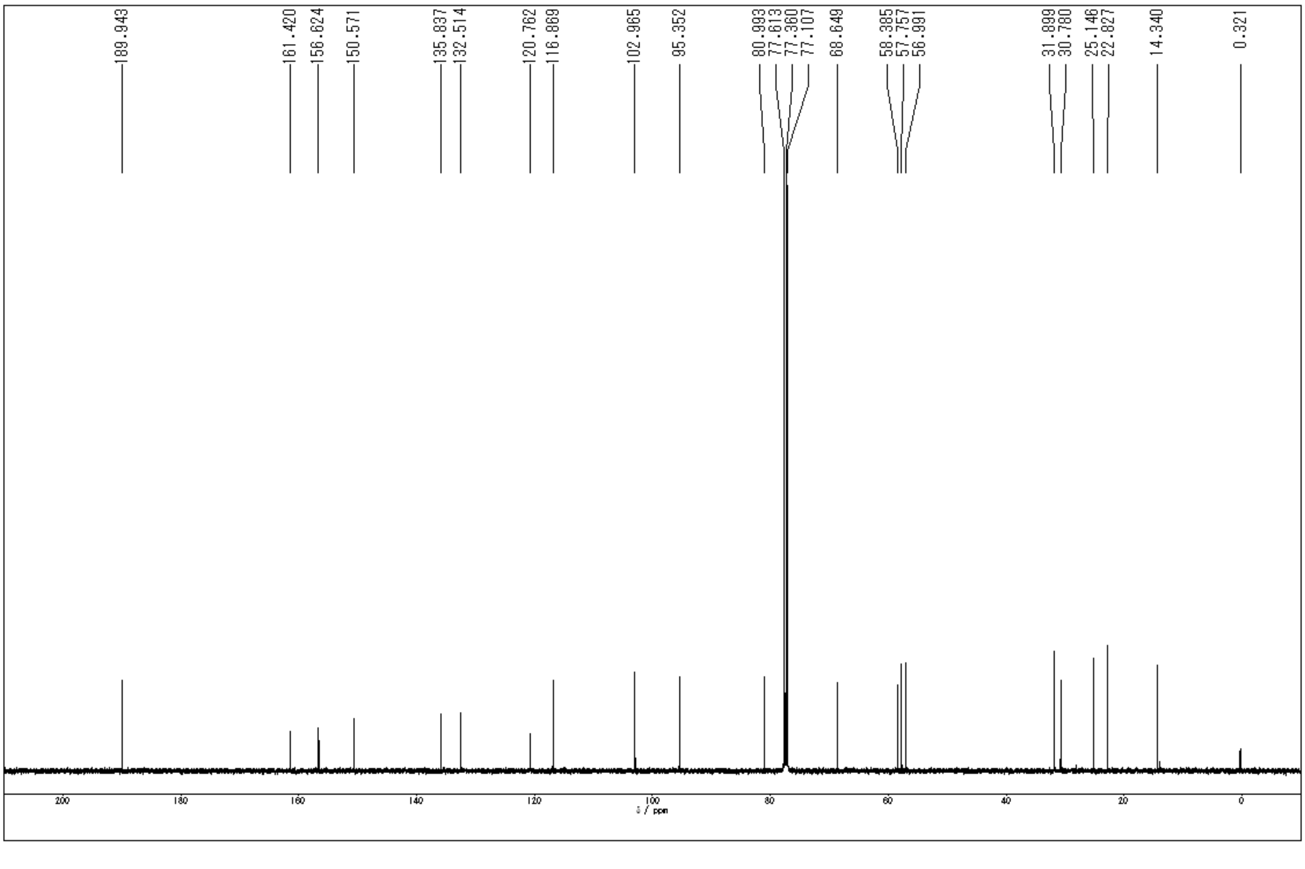

Compound 12

^{1}H-NMR ($CDCl_3$, 400 MHz)

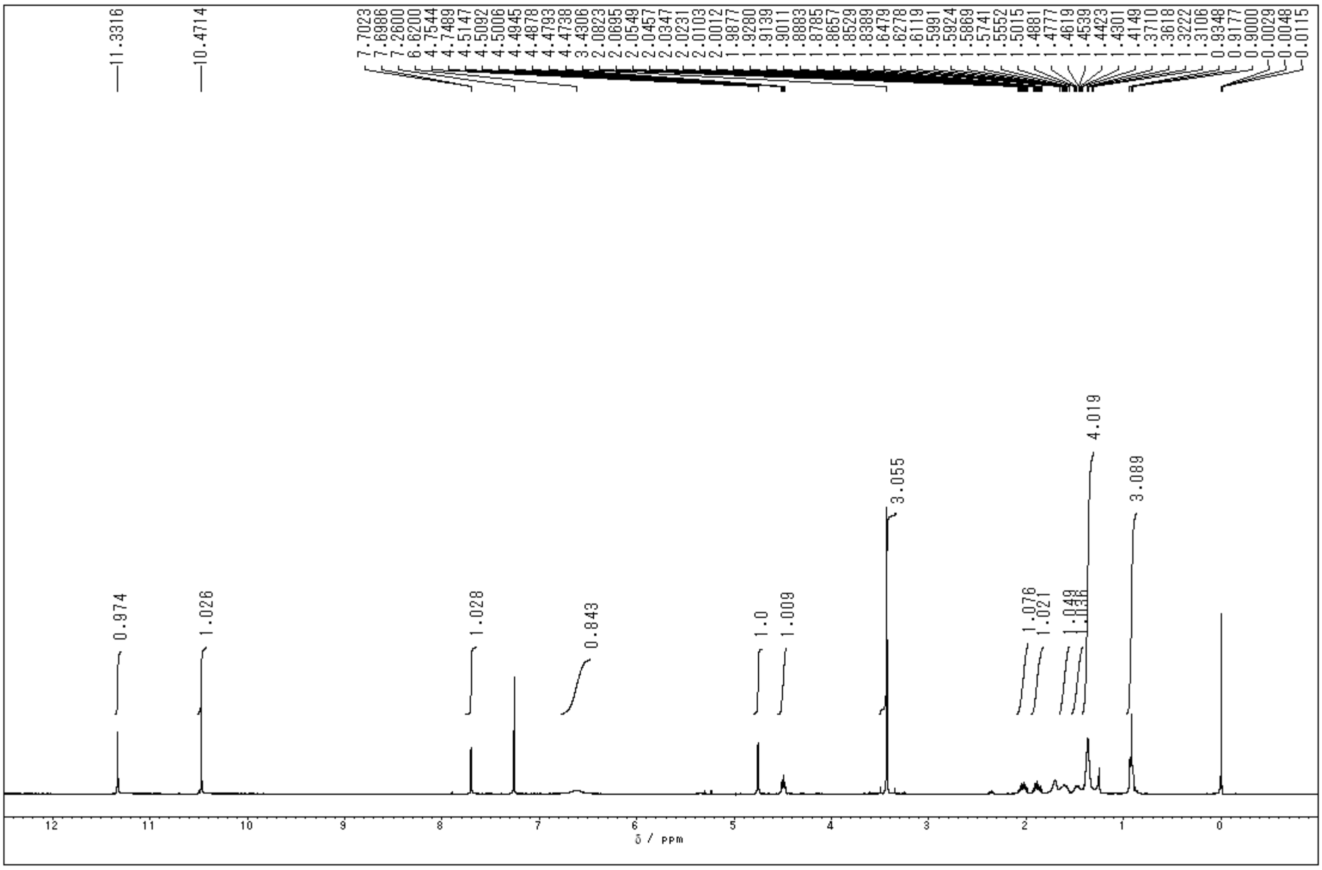

^{13}C-NMR ($CDCl_3$, 125 MHz)

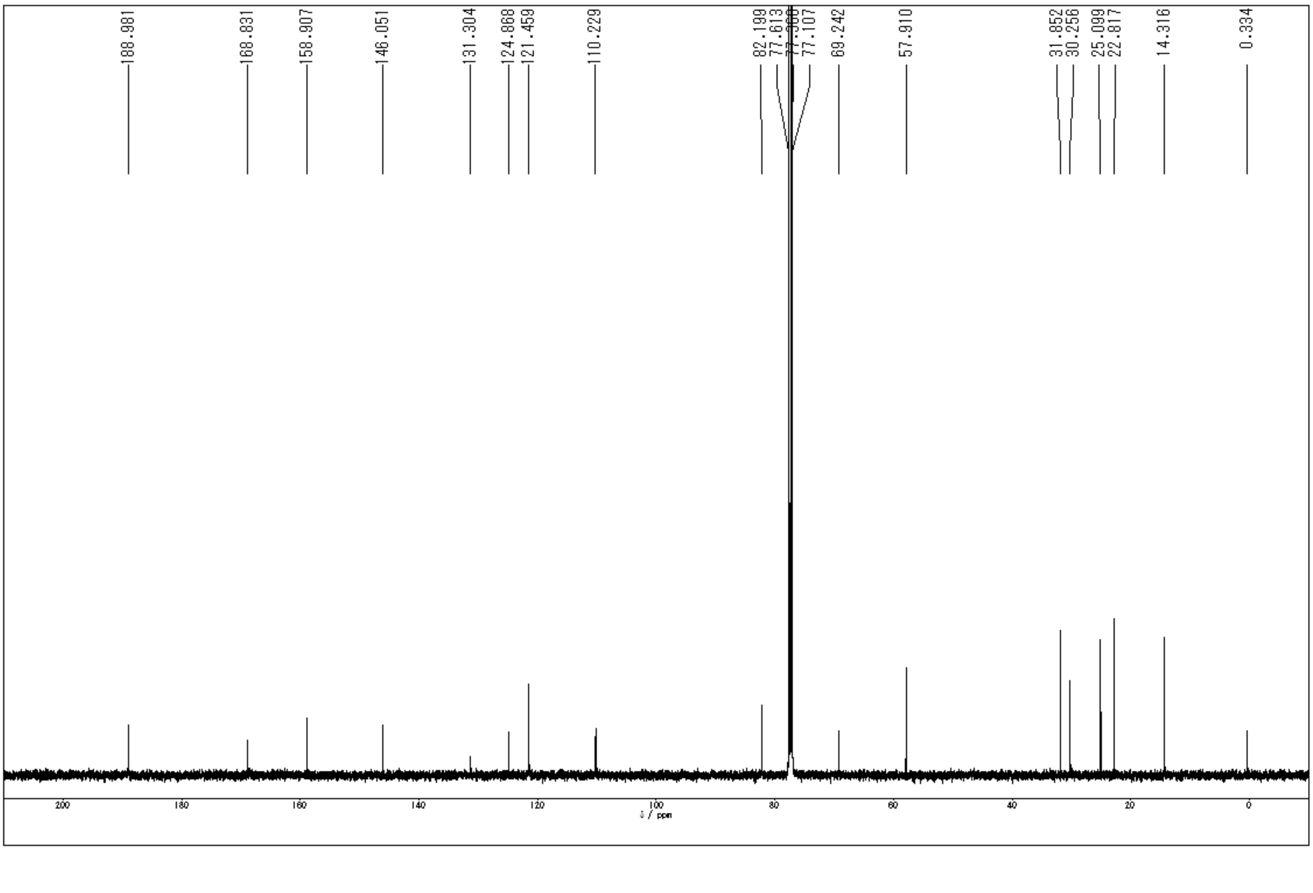

Compound 22

^1H-NMR ($CDCl_3$, 400 MHz)

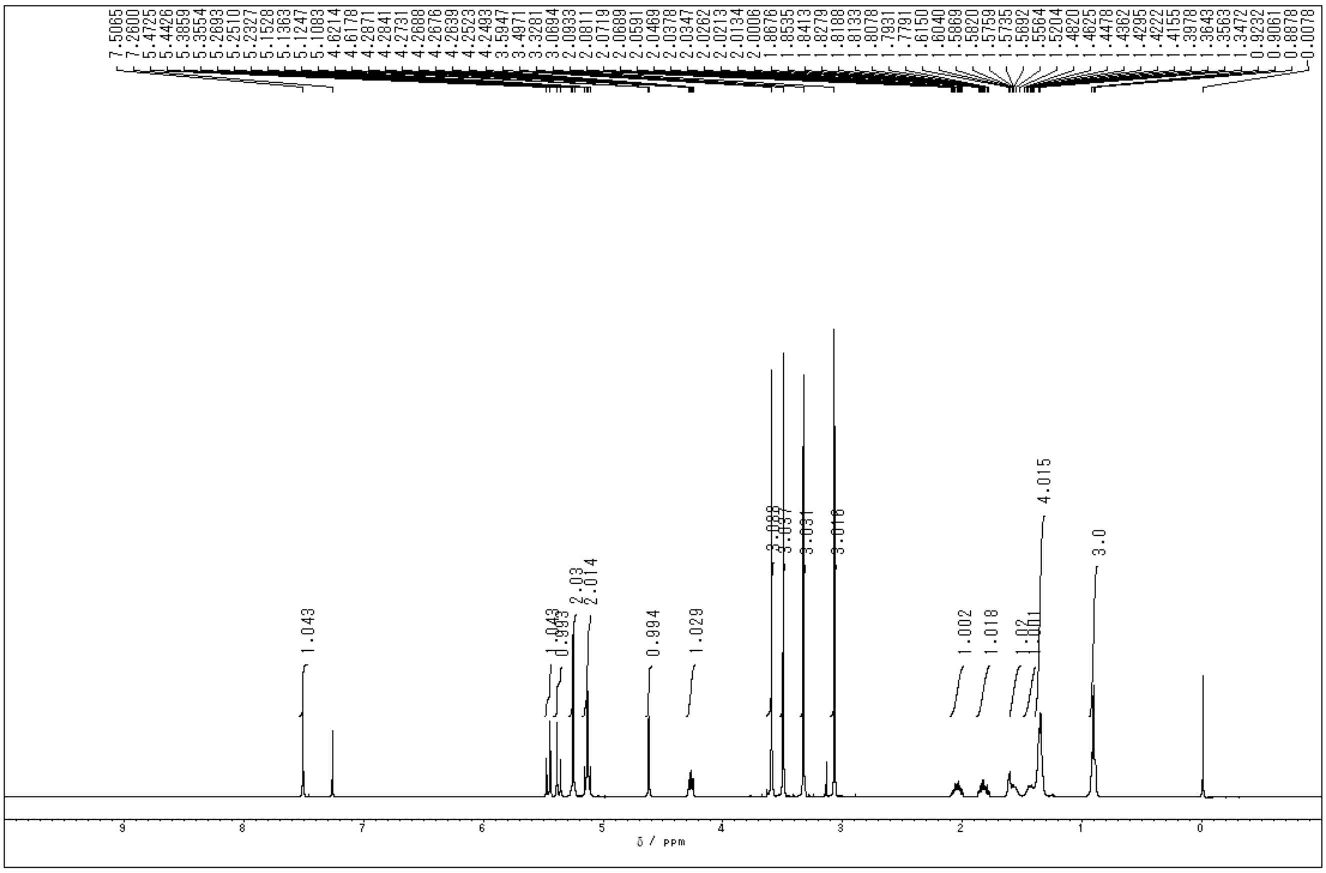

^{13}C-NMR ($CDCl_3$, 100 MHz)

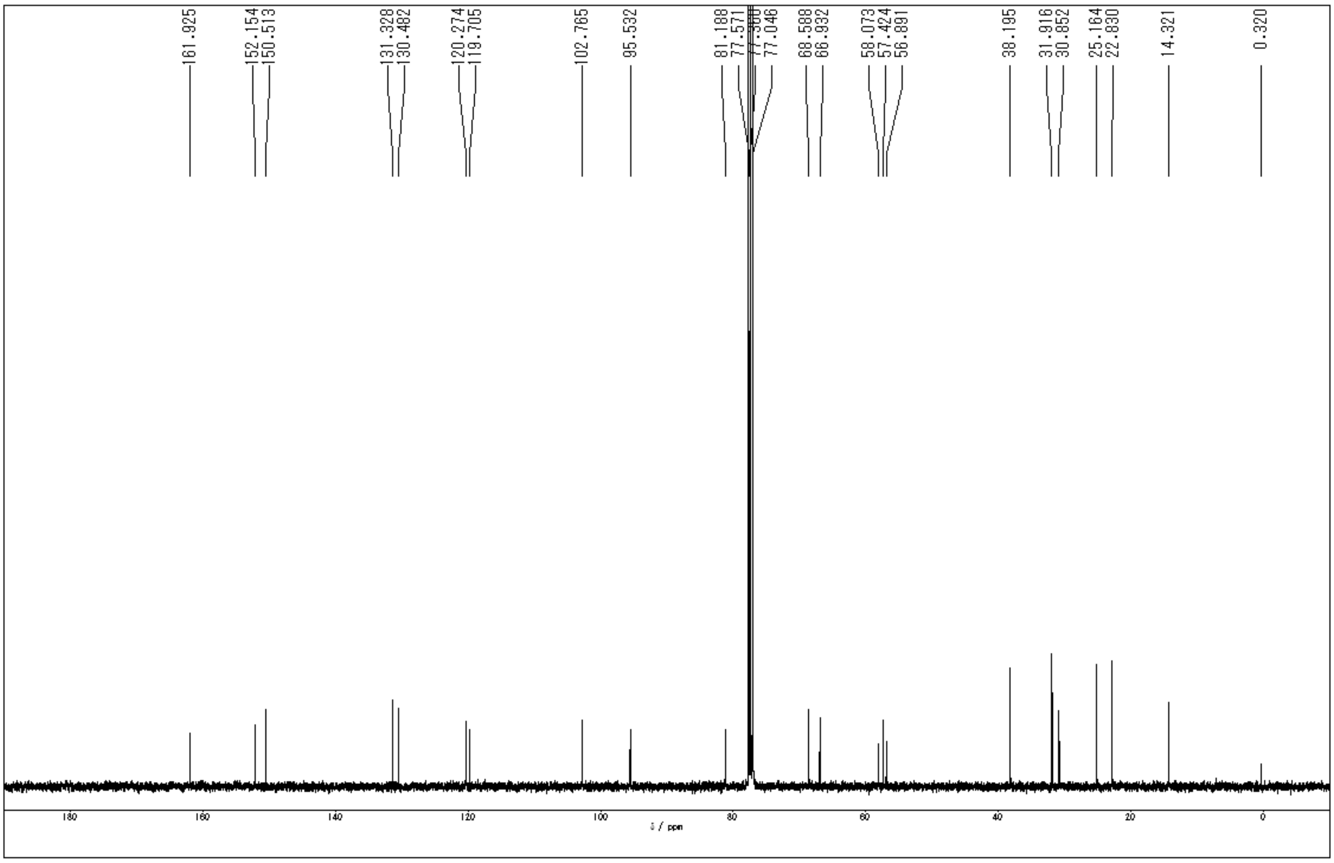

Compound 13a

^{1}H-NMR ($CDCl_3$, 500 MHz)

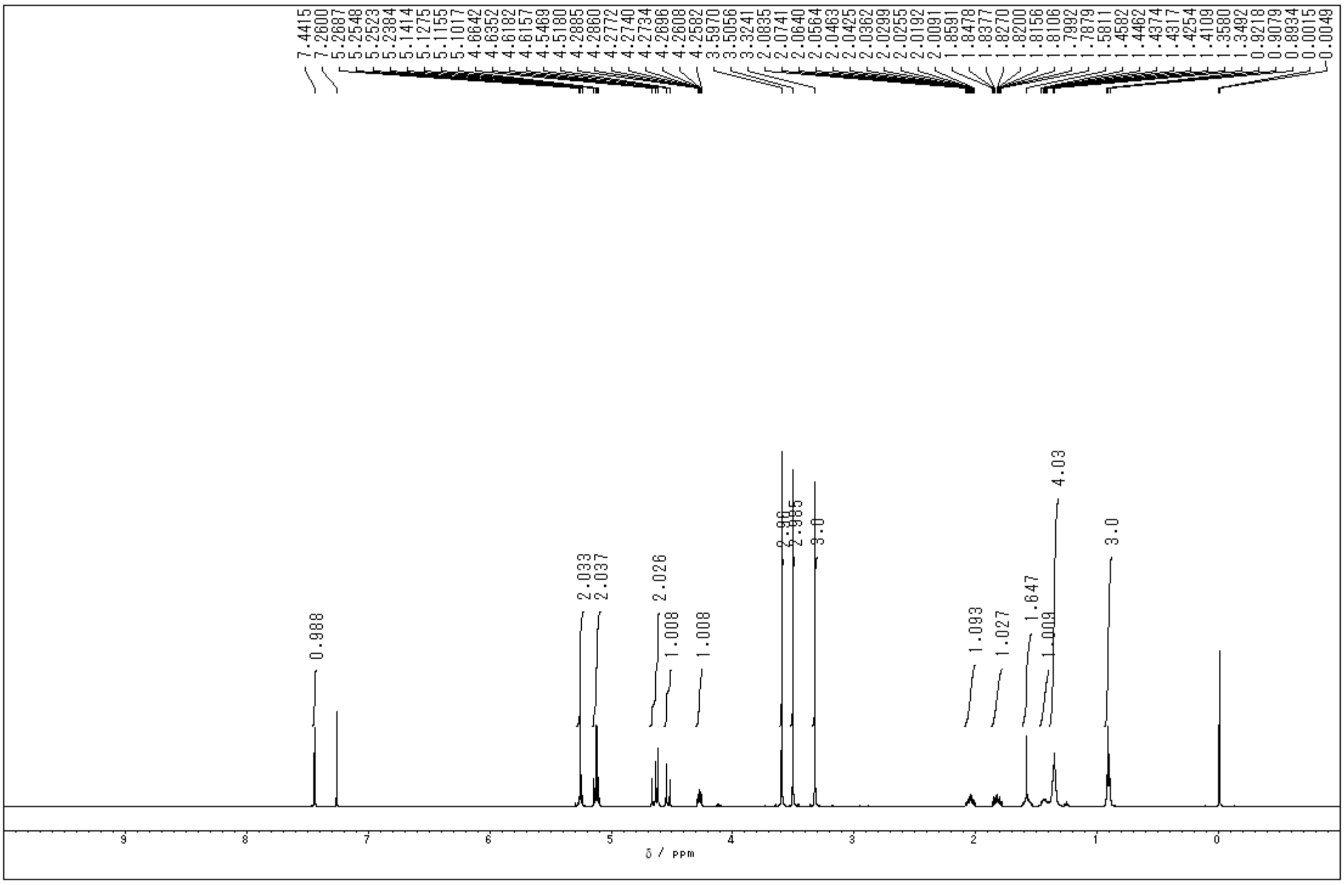

^{13}C-NMR ($CDCl_3$, 125 MHz)

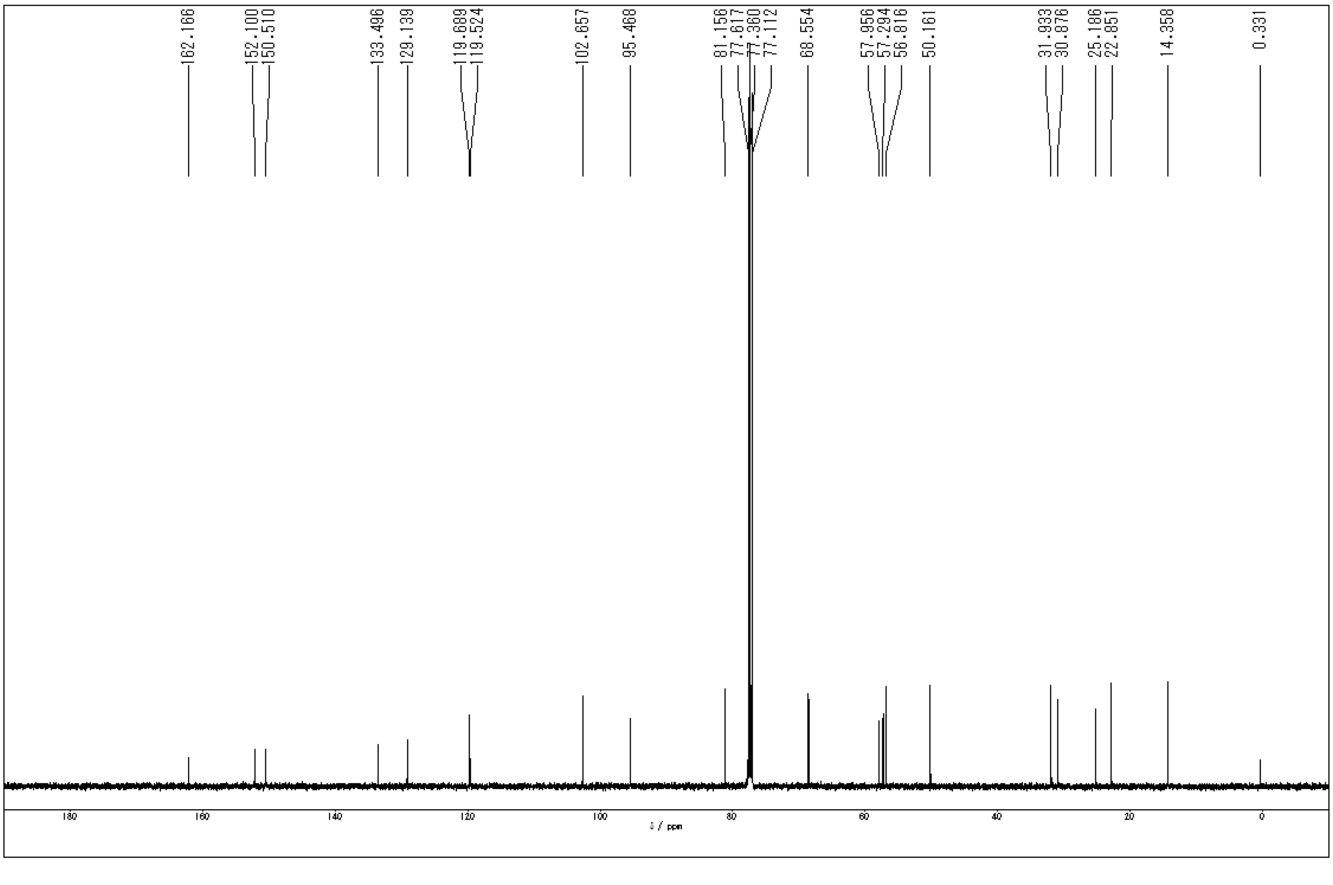

Compound 13

^{1}H-NMR ($CDCl_3$, 400 MHz)

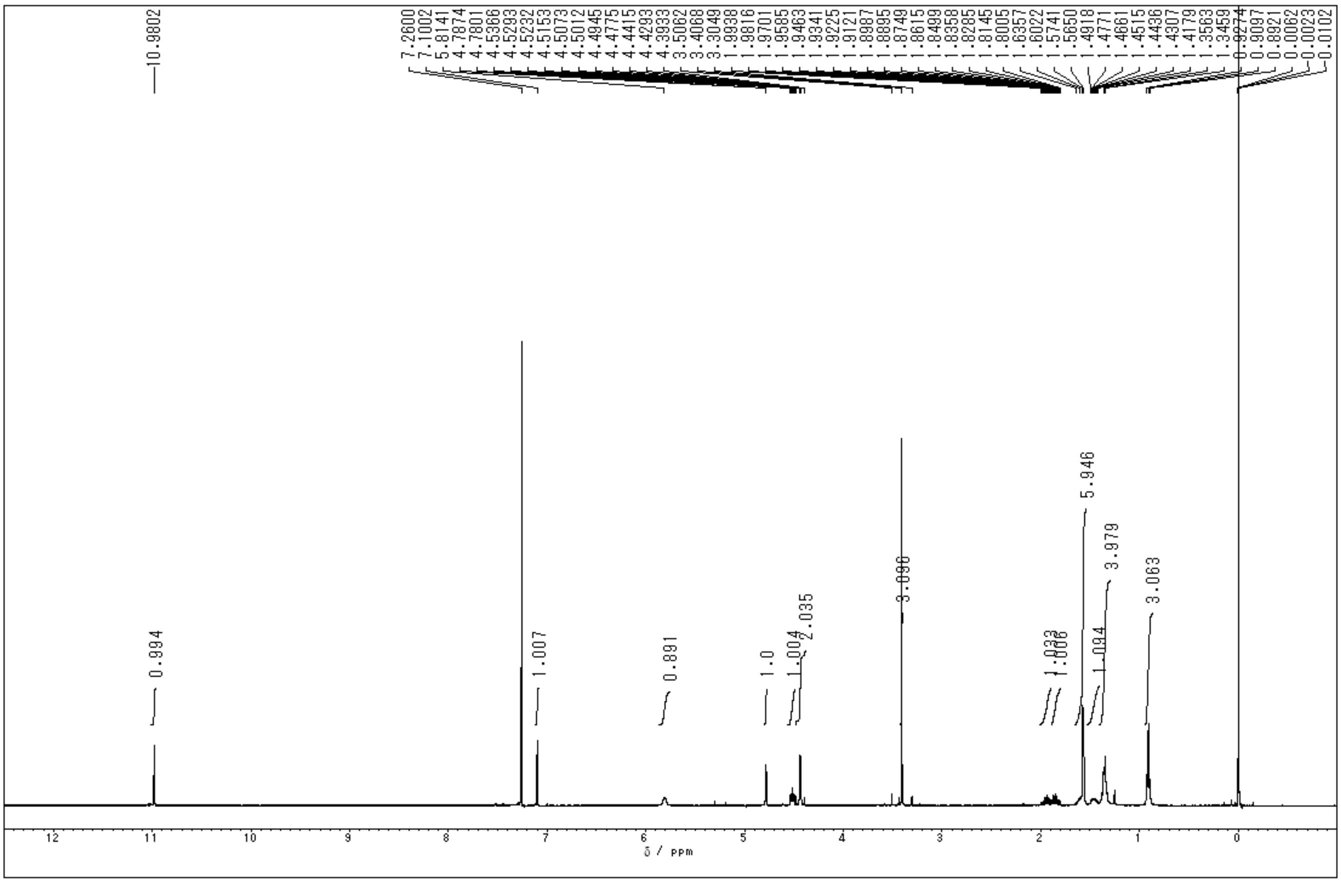

^{13}C-NMR ($CDCl_3$, 125 MHz)

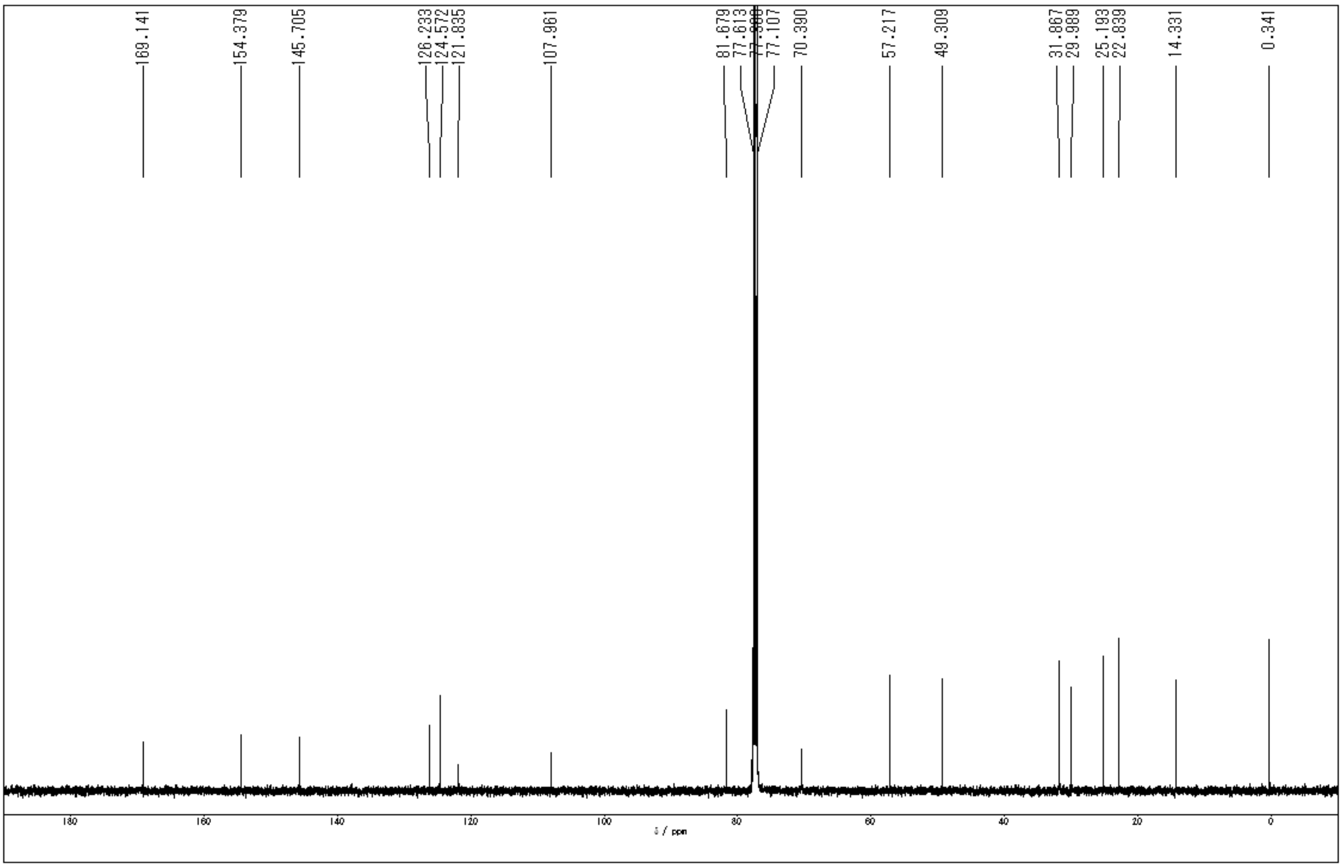

Compound 14a

^{1}H-NMR ($CDCl_3$, 400 MHz)

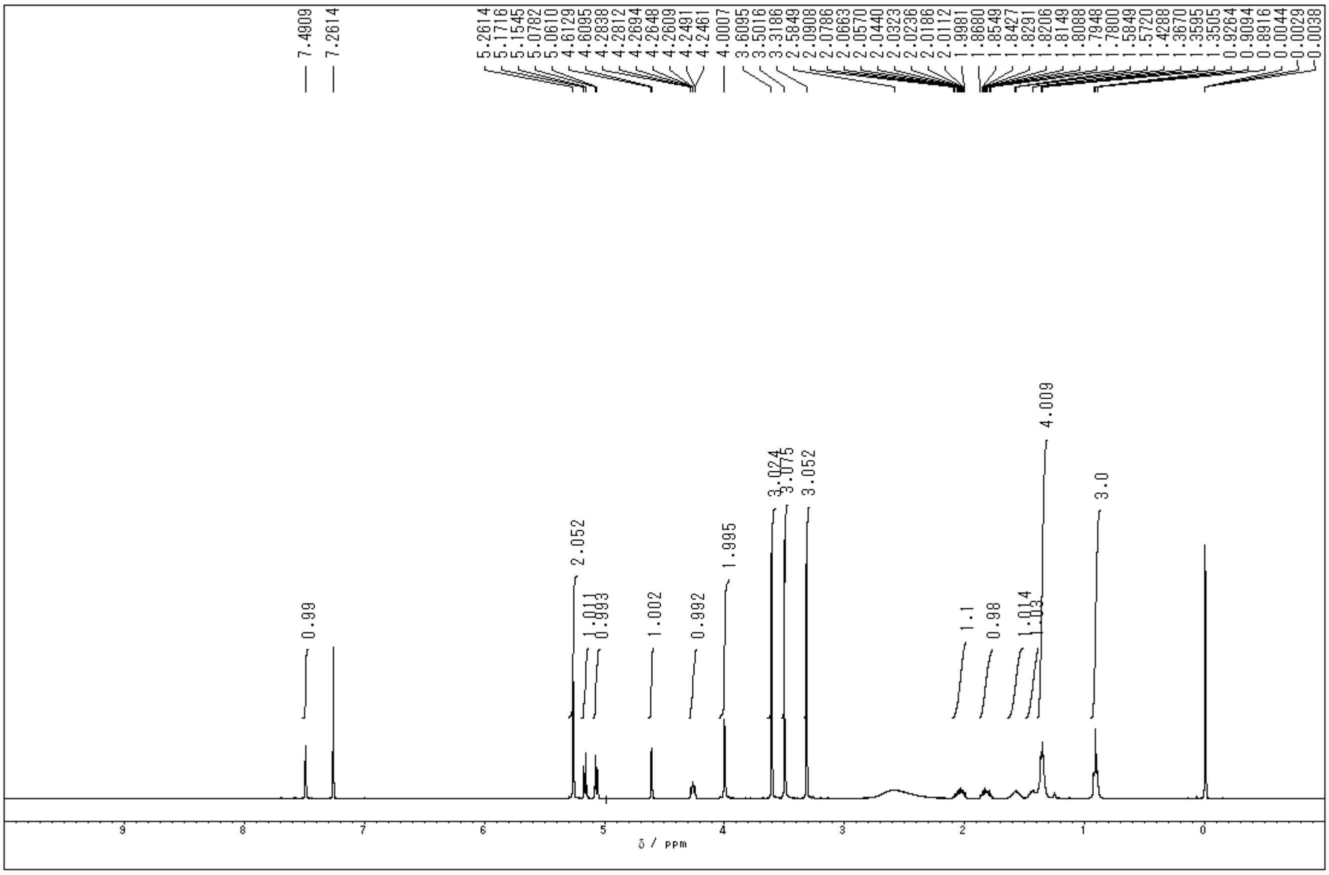

^{13}C-NMR ($CDCl_3$, 125 MHz)

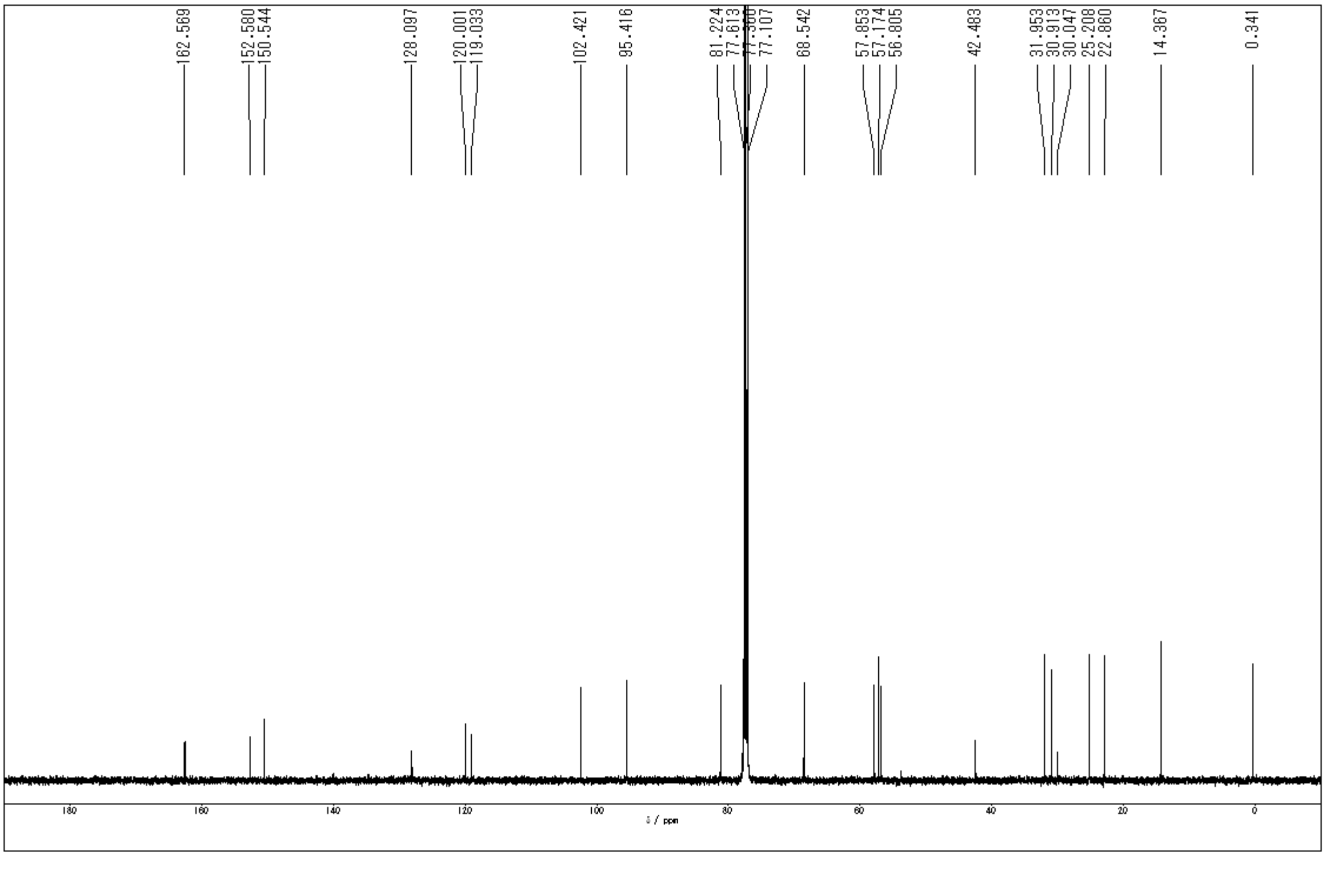

Compound 14

^{1}H-NMR ($CDCl_3$, 500 MHz)

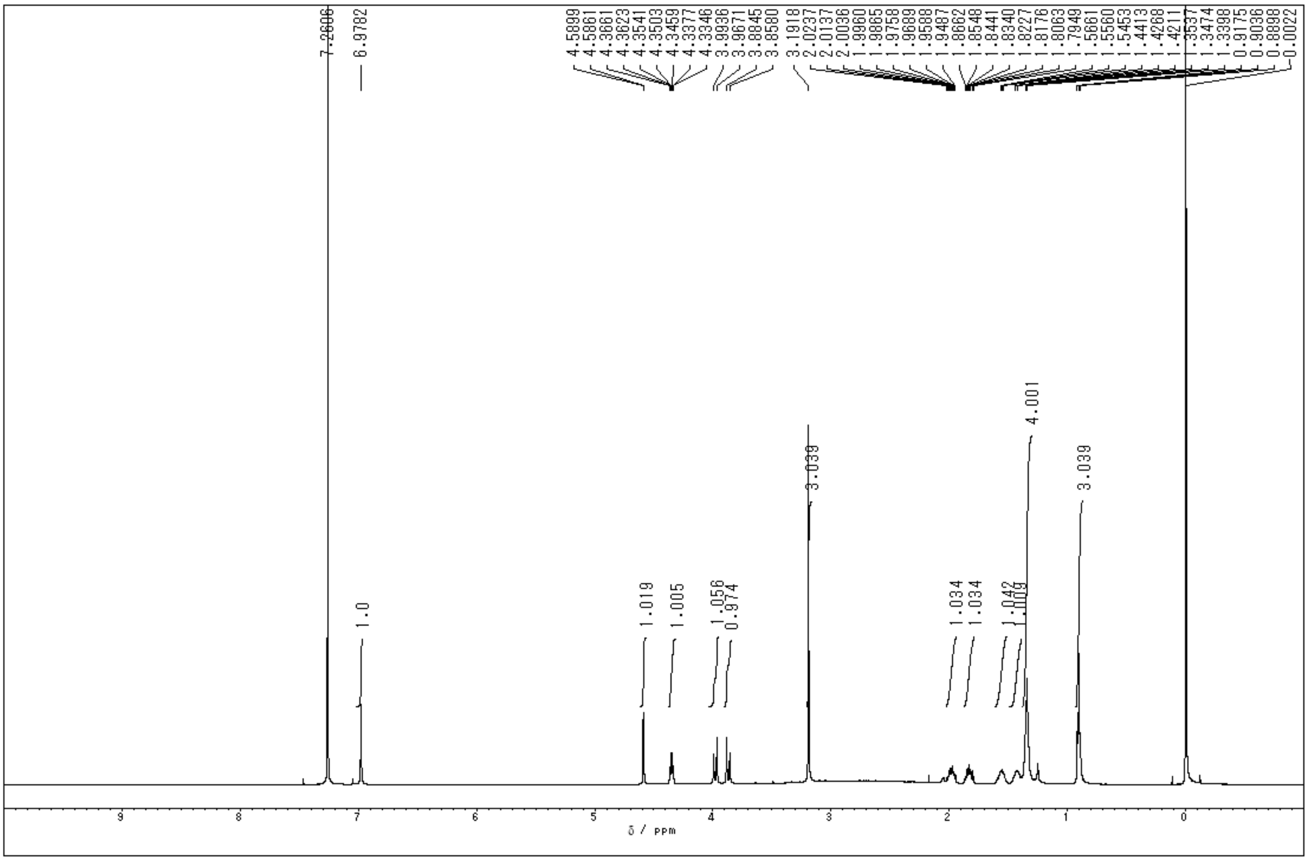

^{13}C-NMR ($CDCl_3$, 125 MHz)

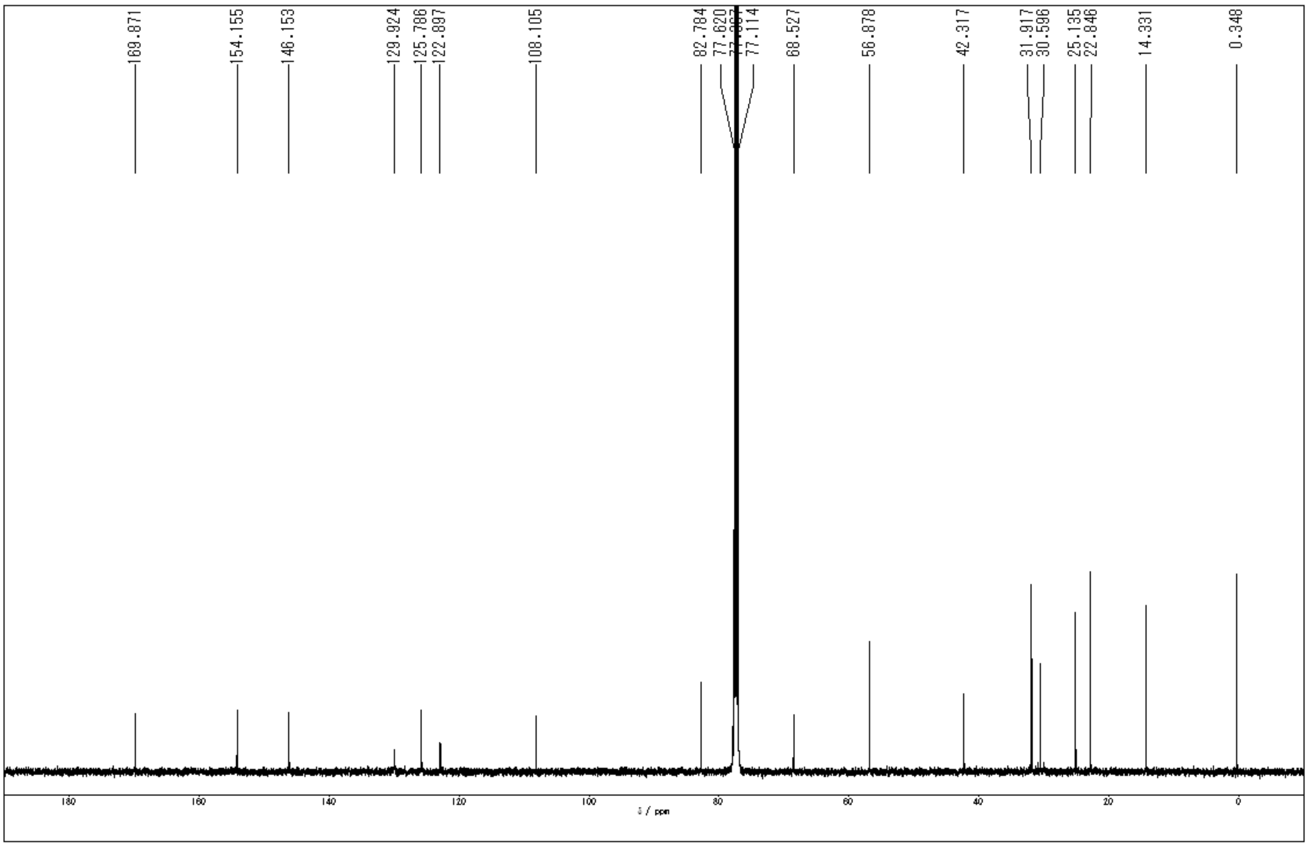

Compound 15

^{1}H-NMR ($CDCl_3$, 400 MHz)

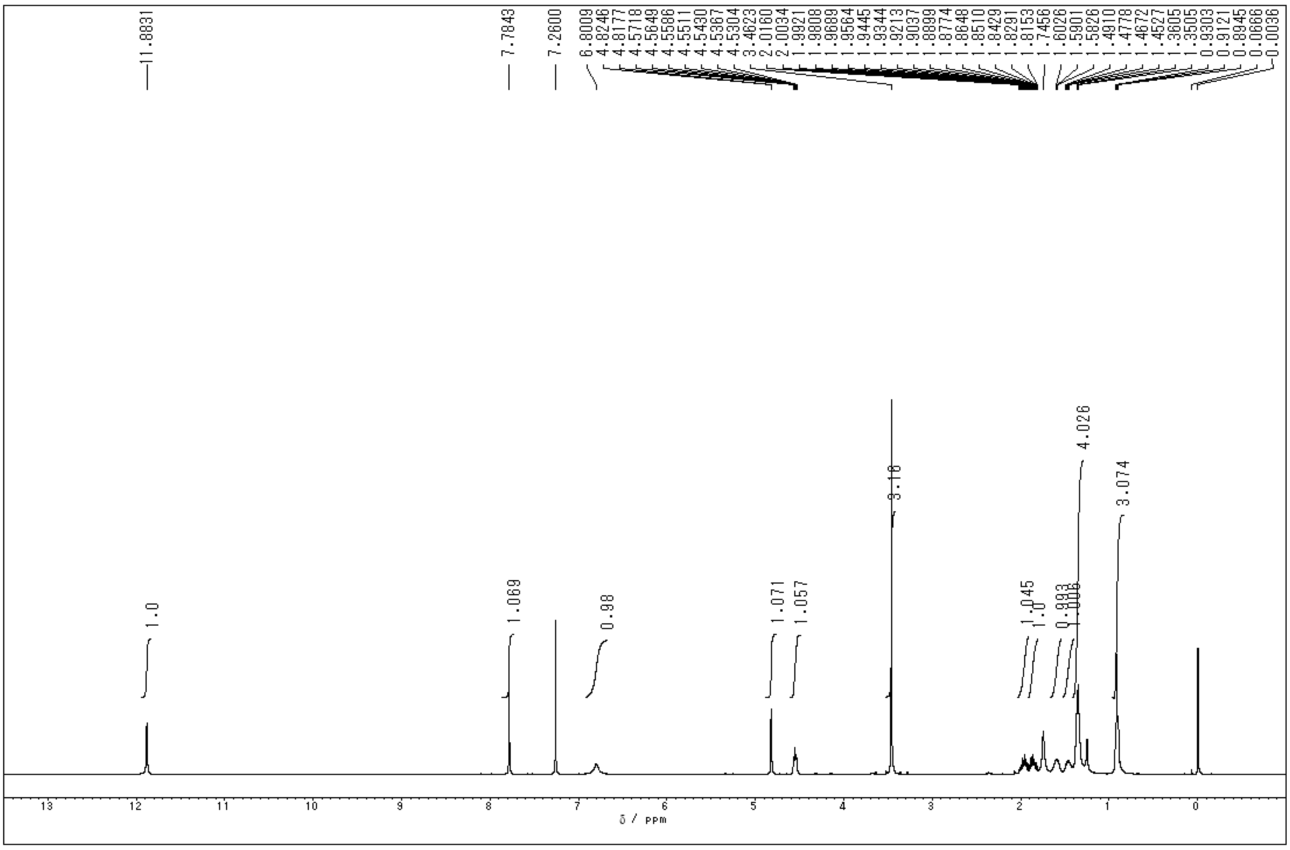

^{13}C-NMR ($CDCl_3$, 125 MHz)

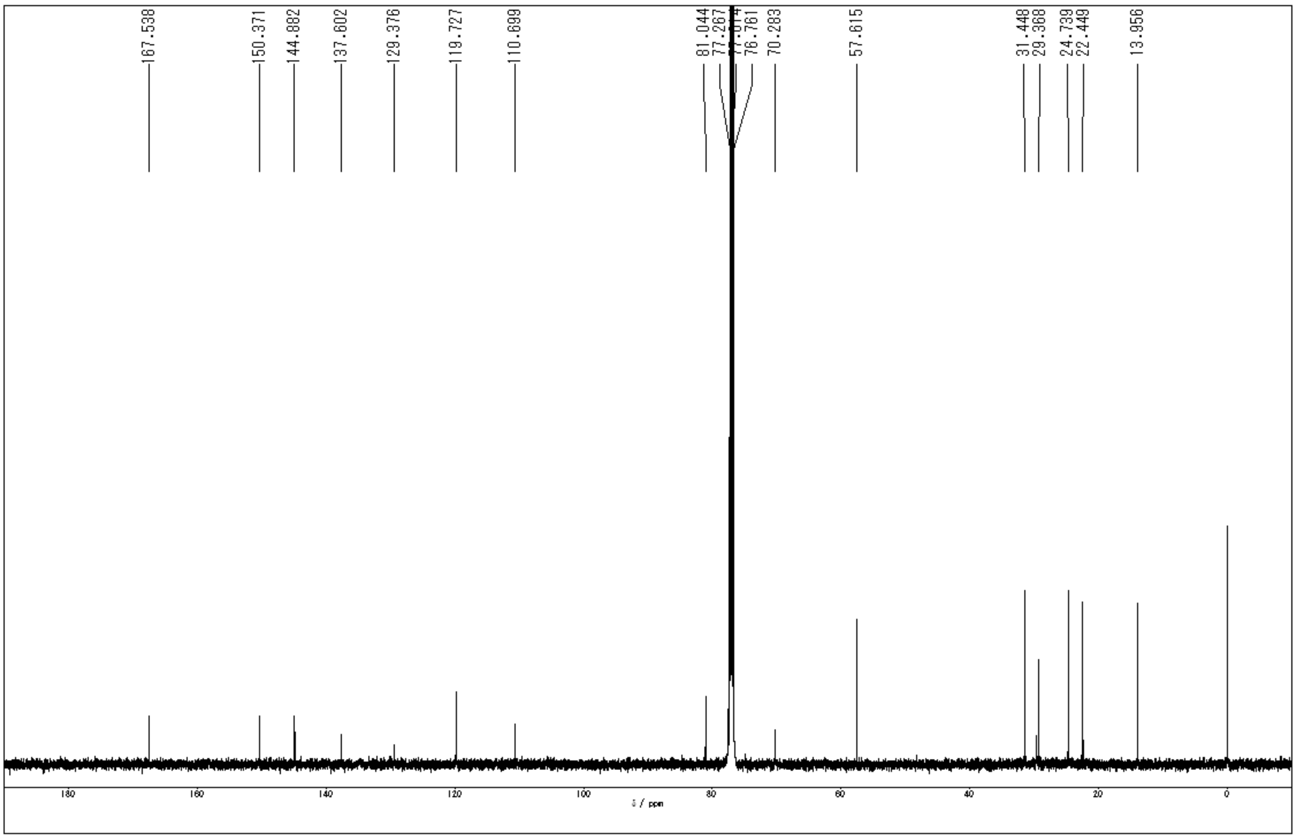

Compound 16

^{1}H-NMR ($CDCl_3$, 500 MHz)

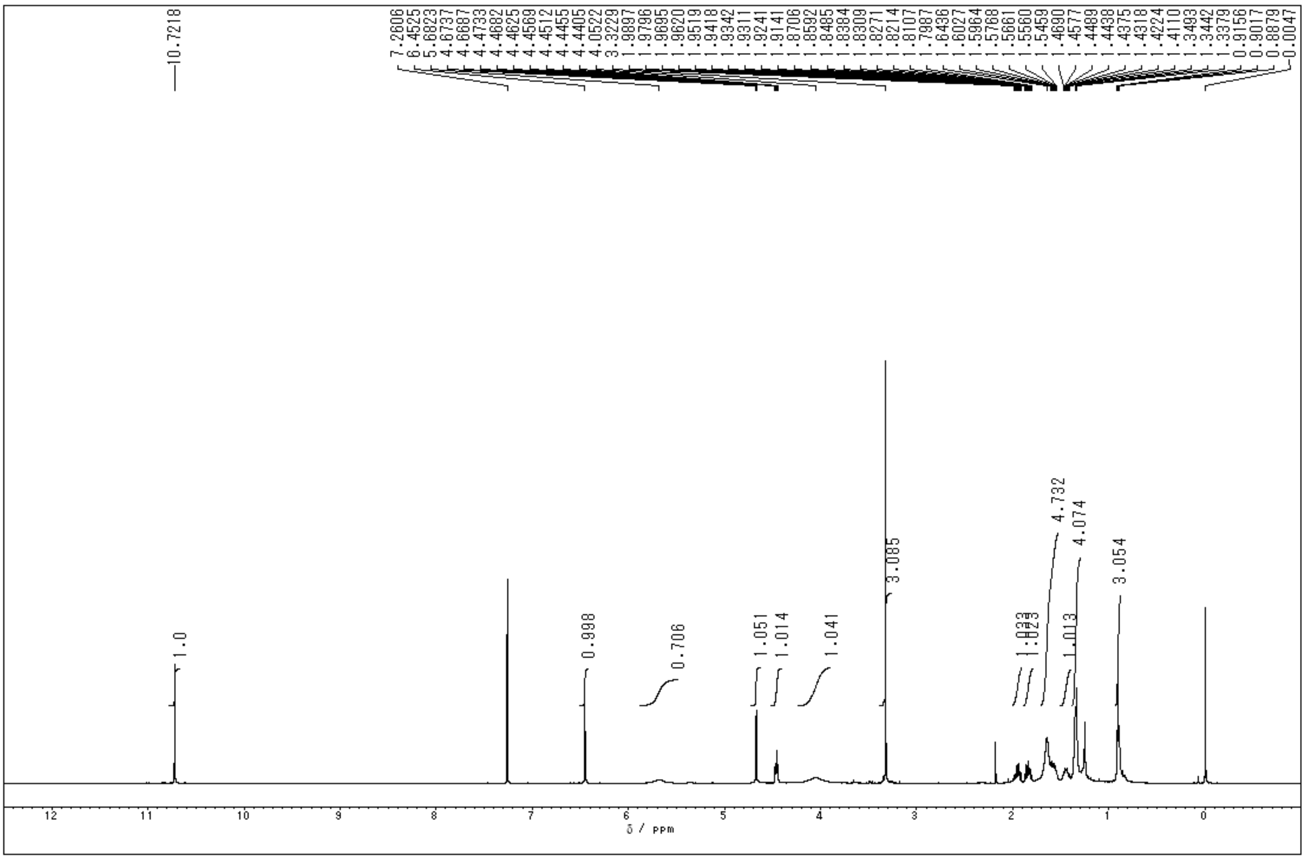

^{13}C-NMR ($CDCl_3$, 125 MHz)

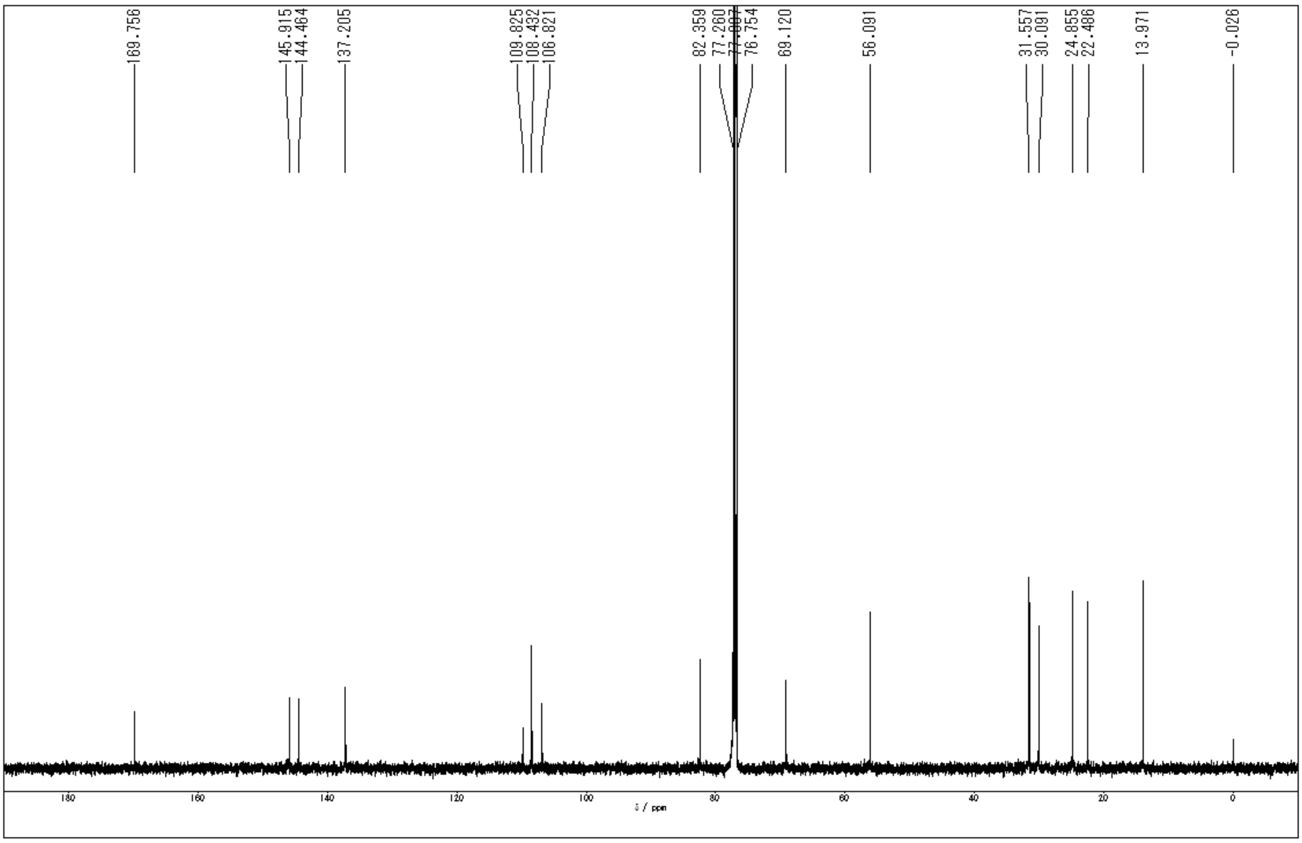

Compound 18a

^{1}H-NMR ($CDCl_3$, 400 MHz)

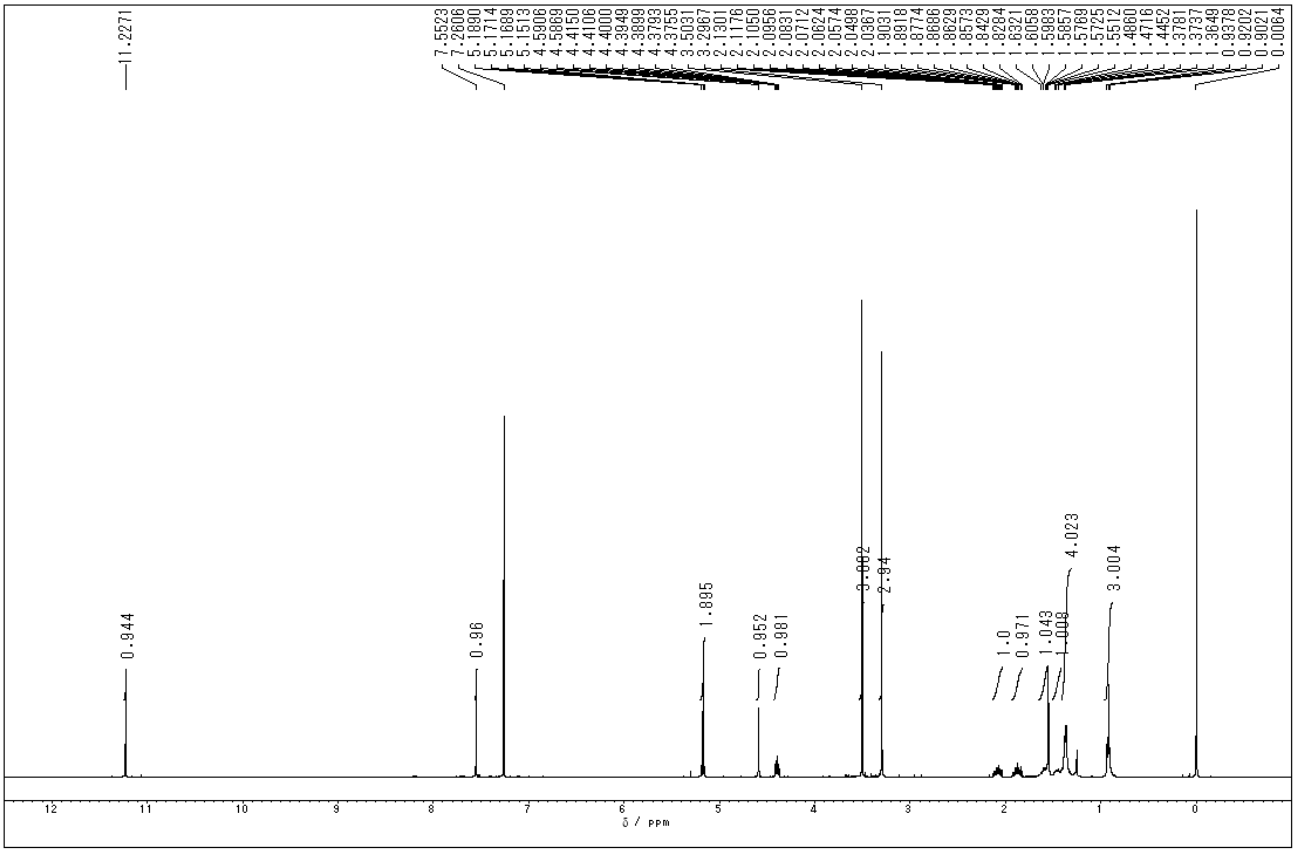

^{13}C-NMR ($CDCl_3$, 125 MHz)

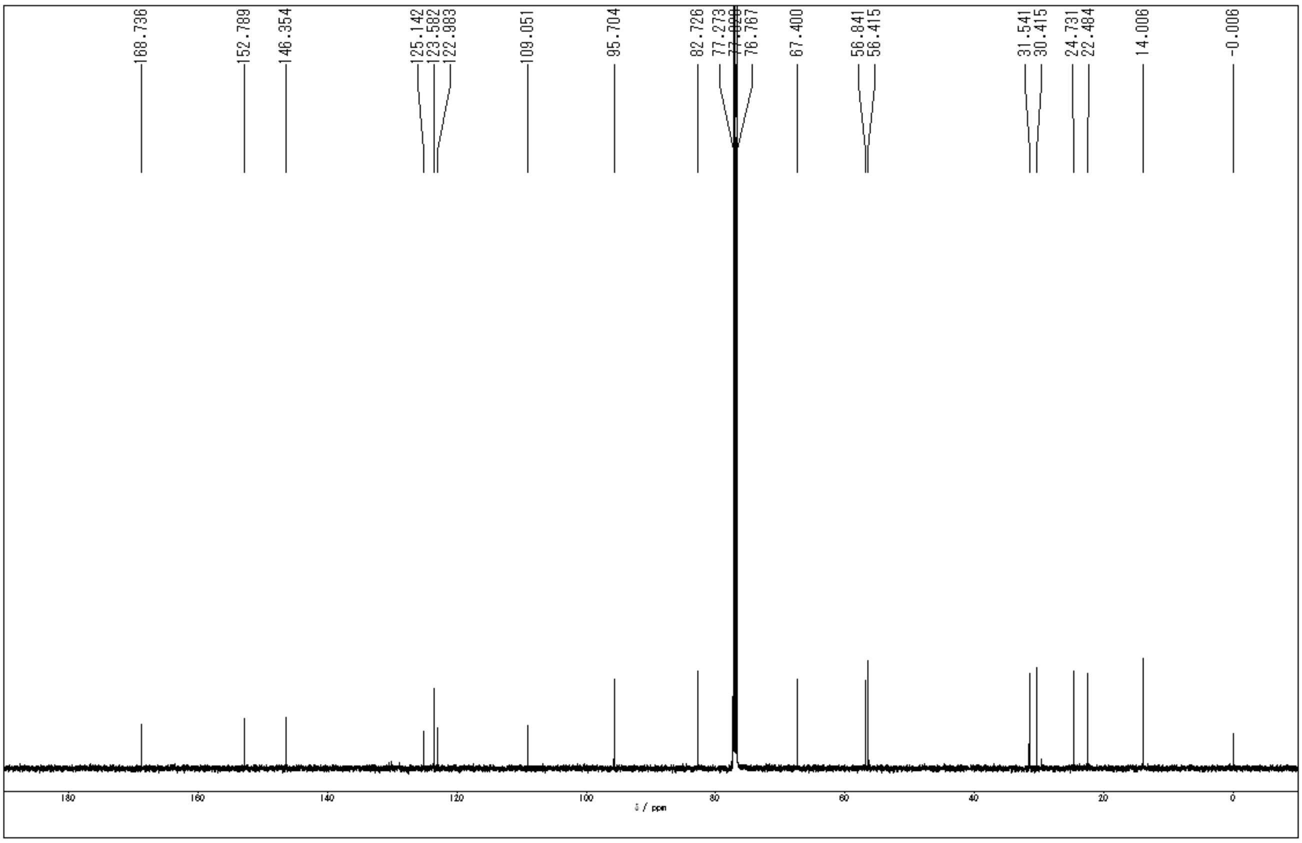

Compound 18

^{1}H-NMR ($CDCl_3$, 400 MHz)

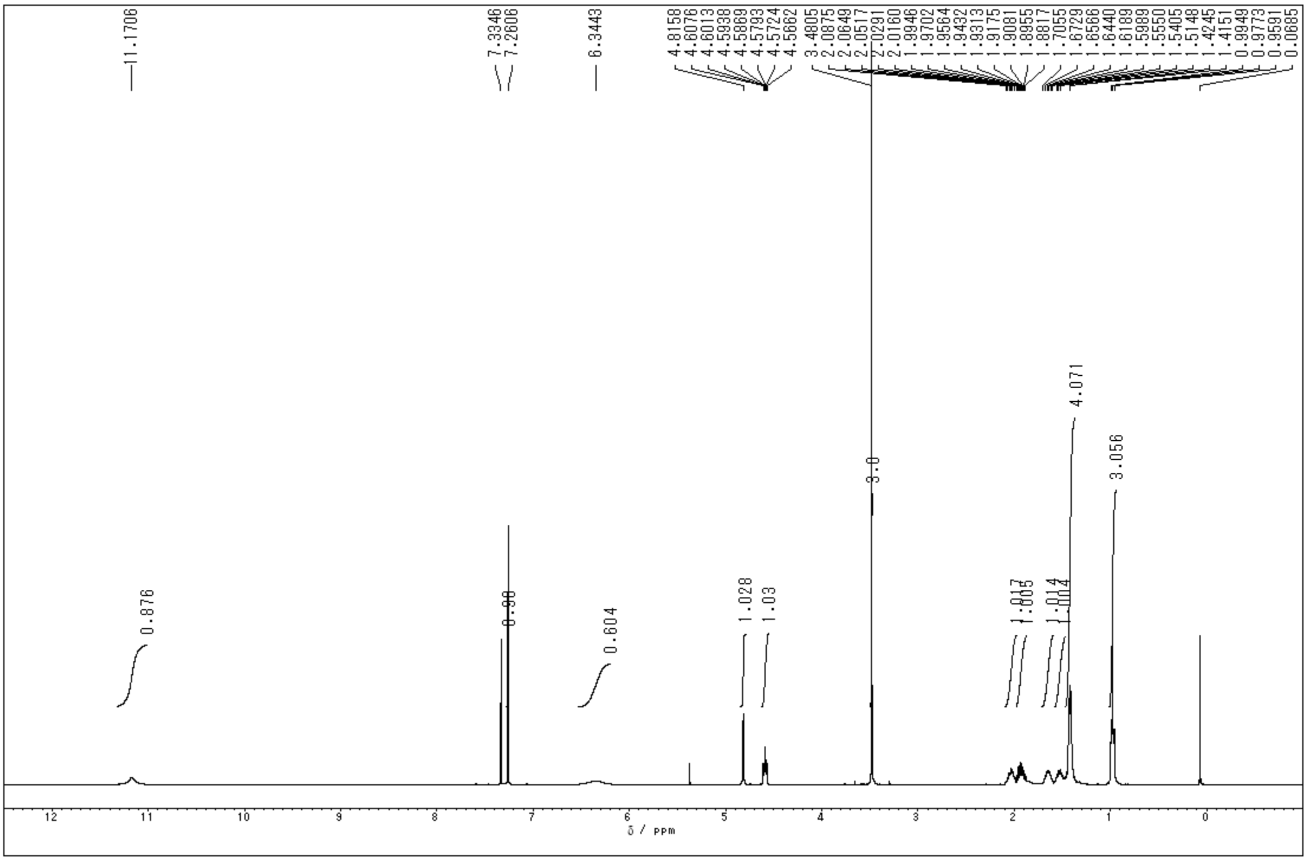

^{13}C-NMR ($CDCl_3$, 100 MHz)

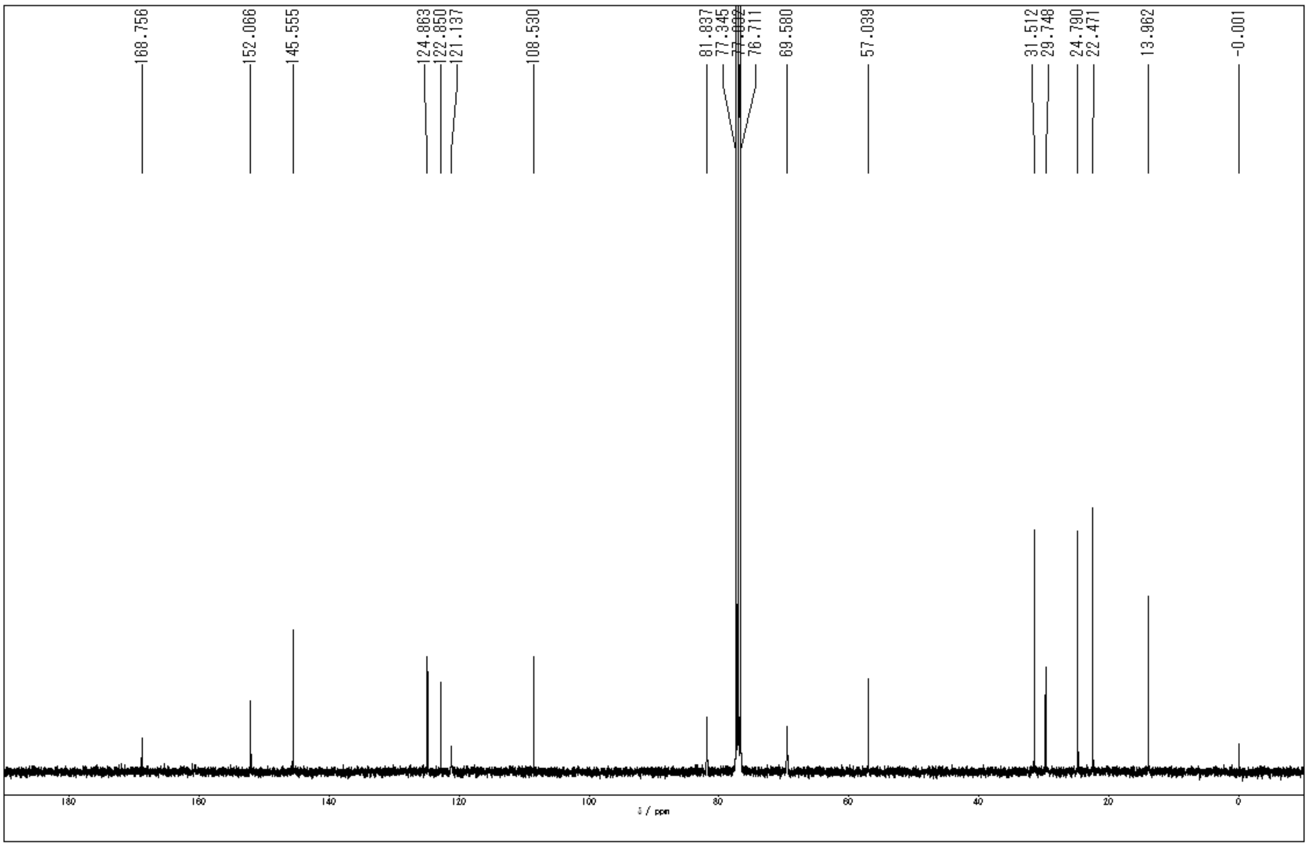

Compound 19

^{1}H-NMR ($CDCl_3$, 400 MHz)

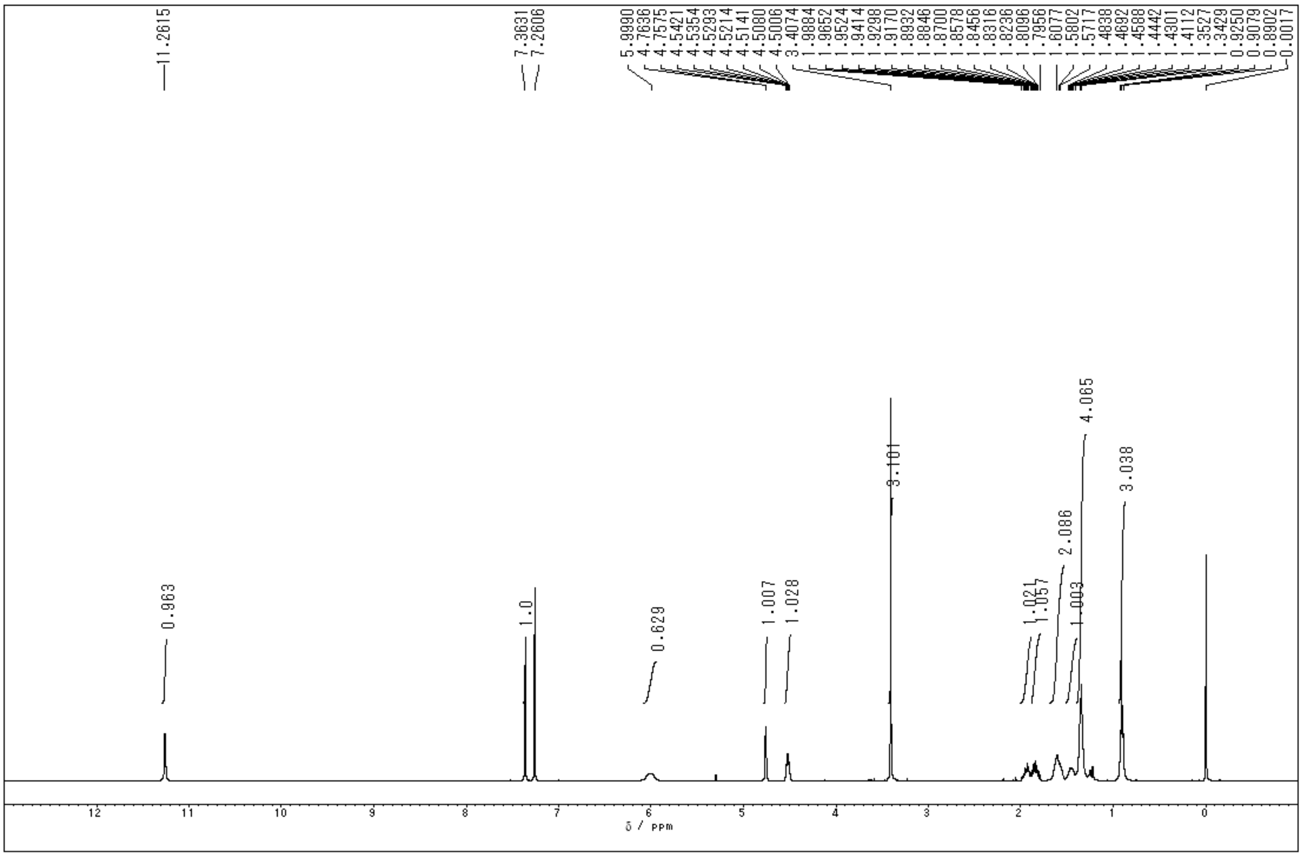

^{13}C-NMR ($CDCl_3$, 125 MHz)

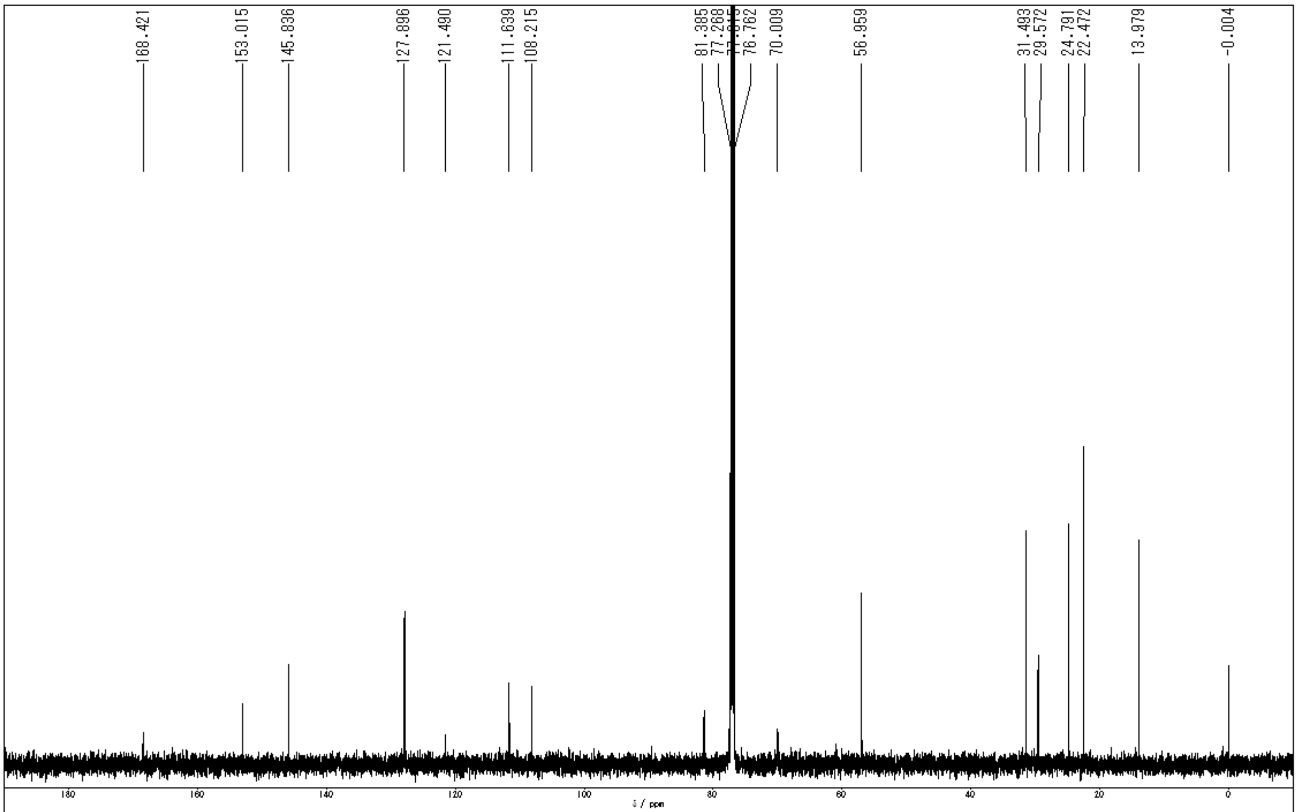

Compound 20

^{1}H-NMR ($CDCl_3$, 500 MHz)

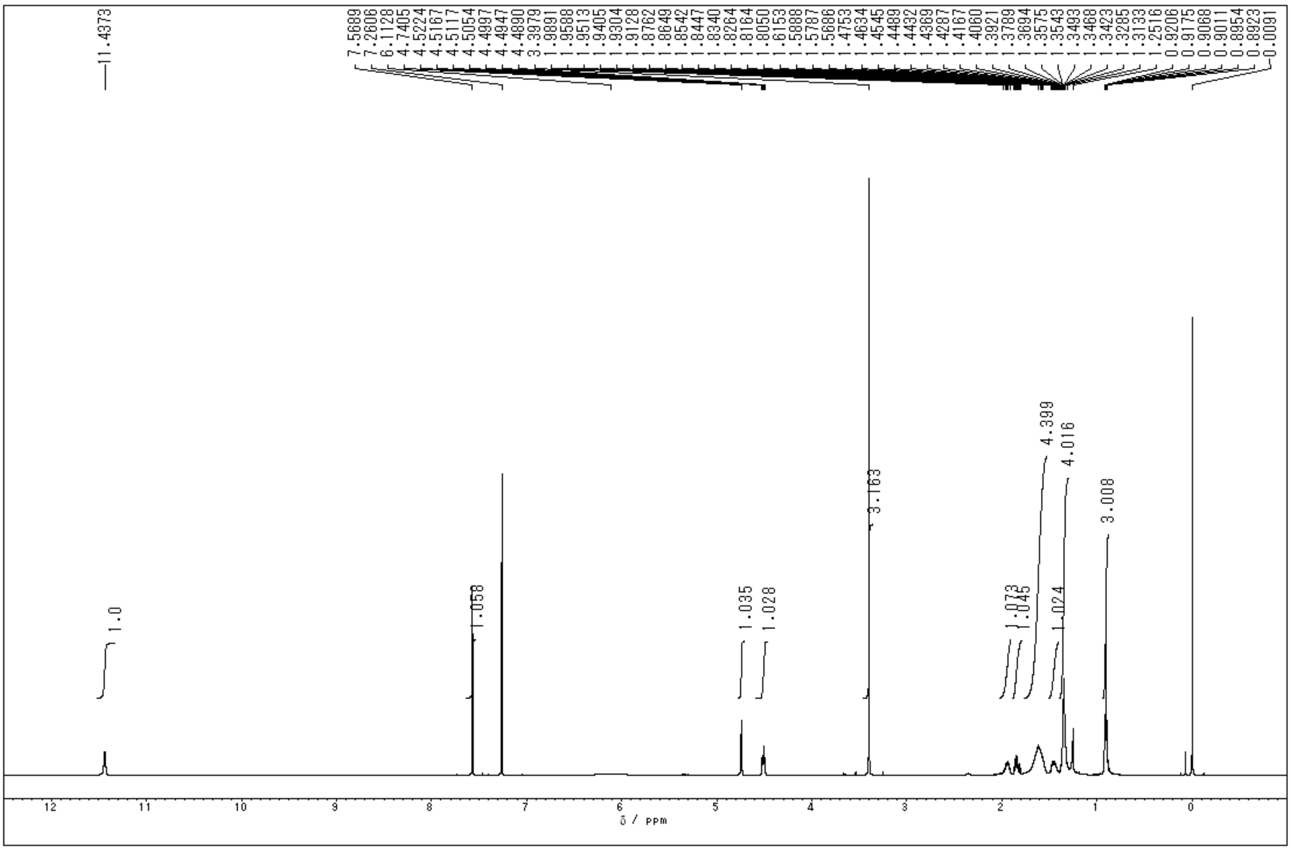

^{13}C-NMR ($CDCl_3$, 125 MHz)

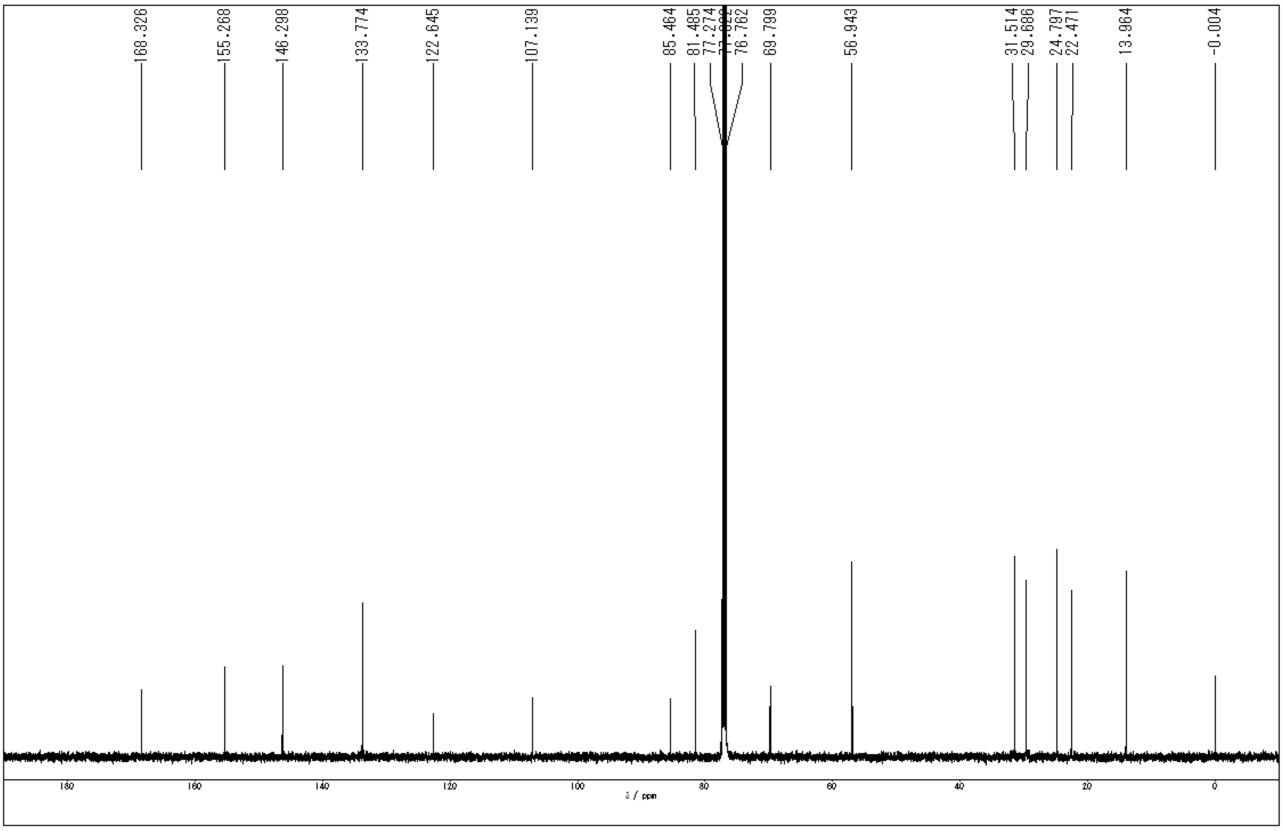

Supplement: Supplementary file 1 [file marinedrugs-18-00092-s001.pdf]
